# High-dimensional Single-cell Analysis Delineates Peripheral Immune Signature of Coronary Atherosclerosis in Human Blood

**DOI:** 10.7150/thno.73336

**Published:** 2022-09-21

**Authors:** Lin Fan, Junwei Liu, Yang Zhang, Chenyun Zhang, Beisheng Shi, Xinyang Hu, Wei Chen, Weiwei Yin, Jian'an Wang

**Affiliations:** 1 Department of Cardiology, The Second Affiliated Hospital, Zhejiang University School of Medicine, Hangzhou 310009, China.; 2 Key Laboratory of Cardiovascular of Zhejiang Province, Hangzhou 310009, China.; 3 Key Laboratory of Integrated Oncology and Intelligent Medicine of Zhejiang Province, Hangzhou 310000, China.; 4 Department of Hepatobiliary and Pancreatic Surgery, Affiliated Hangzhou First People's Hospital, Zhejiang University School of Medicine, Hangzhou 310000, China.; 5 Key Laboratory for Biomedical Engineering of the Ministry of Education, Zhejiang University, Hangzhou 310027, China.; 6 School of Basic Medical Science, Zhejiang University, Hangzhou 310058, China.; 7 Department of Thoracic Surgery, Sir Run Run Shaw Hospital, Zhejiang University School of Medicine, Hangzhou 310016, China.; 8 Present address: Guangzhou Laboratory, Guangzhou, Guangdong 510005, China.

**Keywords:** Atherosclerosis, Cardiovascular disease, CyTOF, Peripheral immune signature, Risk Prediction model

## Abstract

**Rationale:** Pathogenesis of human coronary atherosclerosis is tightly associated with the imbalance of inflammation and resolution in the local immune microenvironment of AS plaques. However, how the peripheral immune system dynamically changes along with disease progression in humans remains unclear. As a result, the minimally-invasive clinical biomarkers that can sensitively distinguish different stages of human coronary atherosclerosis are still lacking.

**Methods:** We performed single-cell Cytometry by Time-Of-Flight (CyTOF) analyses to comprehensively profile the compositions and phenotypes of CD45^+^ cells derived from 83 human peripheral blood samples with two independent antibody-staining panels (T cell panel and myeloid cell panel). Clinical associations between the frequencies of peripheral immune cell subsets with AS plaque burdens of coronary arteries (Gensini score) and serum lipids were also examined. By integrating immune and clinical features, we established novel CVD risk prediction models to stratify patients in different disease stages.

**Results:** We revealed the disease stage-associated peripheral immune features for patients with coronary atherosclerosis (CAS) and atherosclerotic cardiovascular disease (ASCVD), and also identified the specific peripheral immune cell subsets that were tightly associated with the disease severity of coronary arteries (Gensini score). By integrating these peripheral immune signatures with clinical features, we have established a disease progression prediction (DPP) model that could precisely discriminate CAS patients from ASCVD patients with high prediction accuracy (ROC-AUC = 0.88).

**Conclusion:** The progression of coronary atherosclerosis is accompanied by significant alterations of the peripheral immune system, including the changes in the distributions as well as phenotypic functions of specific immune cell subsets. The indicated stage-specific peripheral immune signatures thus become promising minimally-invasive liquid biomarkers that could help to potentially diagnose and monitor the CVD progression in humans.

## Introduction

Cardiovascular disease (CVD) is the leading cause of global deaths, representing over one-third of all deaths worldwide 1. Atherosclerosis (AS), as the primary underlying pathogenesis of CVD, is a chronic inflammatory disorder characterized by endothelial dysfunction, immune cell activations, and the formation of lipid-laden atheroma in the large and medium-sized arteries 2, 3. Immune cells and immune responses are implicated in all stages of atherogenesis, from fatty streaks to mature plaques or even the rupture of vulnerable plaques 4. Coronary artery inflammation is a key modulator in disease initiation and progression, which also influences the high-risk plaques and adverse cardiovascular events, *e.g.*, stable angina pectoris (SAP), unstable angina pectoris (UAP), and acute myocardial infarction (AMI) 5-7. Recent studies have revealed the unprecedented complexity of phenotypes and functionalities of intra-plaque immune cells at the single-cell levels 8-10. Moreover, the elevated serum levels of C-reactive protein (CRP) and interleukin (IL)-6 are detectable in CVD patients 11, 12, suggesting systemic inflammatory responses accompanying the progression of coronary atherosclerosis.

Atherogenesis is initiated by dysfunction of endothelial integrity and retention of cholesterol-carrying low-density lipoprotein (LDL) particles that elicit arterial inflammatory responses, which further recruit an influx of peripheral immune cells into the injured vascular endothelium 13, 14. Monocytes accounting for nearly 5% of peripheral immune cells are playing crucial roles in bridging innate and adaptive immunity and driving inflammatory responses 15. Monocyte-derived macrophage is one of the major immune cell subsets within AS plaques 16. In the initiation phase, circulating monocytes mobilize to oxidized LDL (ox-LDL) or the other athero-antigens, triggering a continuous influx of monocytes towards sub-endothelial space and locally polarizing into diverse phenotypes of tissue-resident macrophages in response to the environmental stimulus. Based on the expressions of CD14 and CD16, peripheral monocyte subsets (*i.e.*, classical-, intermediate-, and non-classical monocytes) are phenotypically and functionally varied, demonstrating diverse pro-inflammatory profiles and migratory potentials in atherogenesis 17. Besides, the distributions and phenotypic shifting of diverse monocyte subsets have been proved to be correlated with CVD prognosis and also could be potential liquid biomarkers and immune targets for treating human atherosclerosis in coronary arteries 18, 19.

Besides monocytes, T cells in peripheral blood are also recruited into the inflamed plaques to closely interact with antigen-presenting cells (*e.g.*, dendritic cells and macrophages) and thereby activate, differentiate, and elicit clonal expansion of antigen-specific TCR repertoires, producing inflammatory cytokines, and thereby deteriorating AS progression 20-23. A previous study has demonstrated that systemic T cell activation also exists in the peripheral blood of patients with stable angina or acute coronary syndrome 24. Further clinical evidence has linked the alterations of specific T cell populations in peripheral blood with CVD risks, including CD4**^+^**CD28^null^, T-helper 1 (Th1), T-helper 17 (Th17), regulatory T (Treg), and CD8**^+^** T cells 25-27. These studies indicate that the distributions and phenotypes of T cells have experienced great changes during atherogenesis, and thus could be served as promising liquid biomarkers to predict CVD risks 28-30.

In the past few decades, multiple CVD risk prediction models have been proposed and used 31-33. Although some of them have achieved good performances in separating non-AS and AS patients 34, 35, the prediction models that could well discriminate patients with coronary atherosclerosis at different stages are still lacking. Despite the increasing of novel biomarkers (*e.g.*, immune-based biomarkers) associated with CVD have been identified, none of these prediction models has considered the immune system (especially peripheral immune signature) as a risk factor in their models yet 36. Given the important roles of immune cells and immunity in CVD pathogenies, in-depth characterization of the disease-specific changes in the distributions and phenotypes of peripheral immune cells could provide additional insight to portray CAS and CVD patients. Therefore, in this study, we comprehensively examined the compositions, phenotypes, and interplays of peripheral immune cells by using single-cell CyTOF analyses, aiming to precisely identify the disease-specific peripheral immune signatures of CAS and ASCVD in humans.

## Methods

### Human Specimens and Ethics Statements

This study was approved by the Ethical Committee and Institutional Review Board of The Second Affiliated Hospital of Zhejiang University School of Medicine (ID: #2017-102). We obtained fresh peripheral blood (PB) samples from The Second Affiliated Hospital of Zhejiang University School of Medicine (Hangzhou, China). All sample donors have provided their informed consent before sample collection.

Inclusion criteria are included: individuals who (1) do not have any AS plaque-induced stenosis in the coronary artery, carotid artery, and lower-limb arteries are enrolled in the non-atherosclerosis healthy control (NC) group; (2) are diagnosed with AS plaque-induced stenosis < 50% in the coronary artery are enrolled in coronary atherosclerosis (CAS) group; (3) are diagnosed with AS plaque-induced stenosis between 50% and 99% in the coronary artery are enrolled in the atherosclerotic coronary vascular disease (ASCVD) group. Exclusion criteria are included: the individual who (1) has tumor diseases, infectious diseases, severe liver/renal damages, or any systemic inflammatory conditions; (2) has received chemotherapy, radiotherapy, or any medications that might impair the systemic immune system; (3) has a history of vasculitis, myocardial infarction, myocarditis, heart failure, or stent/pacemaker implantation; (4) is in pregnancy or perinatal period. In total, we have obtained 83 PB samples, including 13 for NC, 38 for CAS, and 32 for ASCVD. The demographic parameters of sample donors are listed in **Table 1**, and the date of individual recruitment is listed in **Table S1**.

### Single-cell Processing of Peripheral Blood Samples

PB samples were stored in 10 ml EDTA anti-coagulation tubes (BD Biosciences, Cat. No. 366643) at 4 °C after acquisition and processed into single-cell suspensions of whole blood immune cells. Briefly, the samples were centrifuged for 5 min (400 to 500 g, 4 °C) to remove plasma, transferred into a 50 ml tube, and resuspended with a 30 ml ACK Lysis Buffer (Solarbio Life Sciences, Cat. No. R1010) to lyse red blood cells, followed by centrifugation for 5 min (400 to 500 g, 4 °C) to remove supernatant. Afterward, the cells were washed twice with PBS buffer, resuspended, counted, and stored on ice.

### Antibody Labeling, Staining, and Barcoding by CyTOF

Two pre-defined antibody-staining panels, including T cell panel **(Table S2)** and myeloid cell panel **(Table S3)**, were independently used for single-cell CyTOF analyses. Metal-tag-conjugated antibodies were either directly purchased, or in-house made by conjugating the pure antibodies with corresponding metal tags using MAXPAR Antibody Labeling Kit (DVS Sciences) by following the standard protocol as previously described 37. Metal-tag-conjugated antibodies were titrated into the optimized staining concentration and diluted to 0.5 mg/ml in Antibody Stabilization Solution (CANDOR Bioscience, Cat. No.55514) for storage at 4 °C.

A total of 6 × 10^6^ cells from each PB sample were collected and split into two equal aliquots, which (3 × 10^6^ cells per sample) were stained with the indicated conjugated antibodies of the T cell panel and myeloid cell panel, respectively. Briefly, the cells were resuspended and stained with Live/Dead ^194^Pt Cisplatin (Fluidigm, Cat. No. 201194) for 5 min and incubated with Fc Receptors Blocker Mixture (Equitech-Bio; anti-human/mouse/hamster/rat IgG) to block the non-specific Fc binding. Next, the cells were washed twice and incubated with a pre-configurated-antibody cocktail of cell surface markers for 30 min on ice, and then washed twice by Cell Staining Buffer (CSB; BioLegend, Cat. No. 420201), followed by incubation with Fix and Perm Buffer (Fluidigm, Cat. No. 1960962) that supplemented with 250 μM ^191^Ir and ^193^Ir DNA Intercalator (Fluidigm, Cat. No. 201192B) at 4 °C for overnight. On the next day, the stained cells were washed twice by CSB and then incubated with a pre-configurated-antibody cocktail of intracellular markers in Permeabilization Buffer (eBioscience, Cat. No. 00-5523-00) for 30 min on ice. To minimize the batch effects, the stained cells from different PB samples were barcoded with palladium isotopes, *i.e.*, ^104^Pd, ^105^Pd, ^106^Pd, ^108^Pd, and ^110^Pd (Trace Sciences) by following the standard protocol 38, 39. Afterward, the stained cells were washed twice by CSB, counted, and pelleted until loaded to the CyTOF platform (Fluidigm, USA).

### CyTOF Analysis

Single cells of PB samples were analyzed by CyTOF as previously described 40. Before loading the single-cell suspensions of PB samples into CyTOF, we performed a tuning and quality control procedure to calibrate CyTOF with Tuning Solution (Fluidigm, Cat. No. 201072) and EQ Four Element Calibration Beads (Fluidigm, Cat. No. 201078). The cells then were pelleted and resuspended to 1×10^6^ cells/ml in double-distilled water (ddH_2_O; mixed with 20% EQ Beads) and passed through a 35 μm filter cap into a FACS tube (BD Biosciences, Cat. No. 352054). All parameters of CyTOF were set on the default mode, and raw data of PB samples were collected at an average rate of 300 to 500 events/s.

CyTOF raw data (.fcs) was firstly de-barcoded by using a doublet-filtering scheme 41, and EQ Four Element Beads were used as a standard reference to normalize the expression intensities of markers 42. Debris and dead cells were manually gated out with FlowJo (v10.0.7, Tree Star) based on the following parameters: Event length, DNA Intercalator iridium (^191^Ir and ^193^Ir), and Cisplatin (^194^Pt) as previously described 43. Then, the data for each sample was transformed by using the Arcsinh function with a cofactor of 5 and then pooled together for the downstream clustering analyses. Phenotyping by Accelerated Refined Community-partitioning (PARC) algorithm 44 was applied to cluster the targeted cells and partition these immune cells into distinct phenotypes based on the expressions of typical immune lineage markers. Manual gating in FlowJo (v10.0.7, Tree Star) was applied to partition the cells into different immune cell types 39, 40, and major cell types were identified by using the conventional lineage markers (*e.g.*, CD45, CD66b, CD3, CD4, CD8, CD19, CD56, CD14, CD16, CD33). t-distributed stochastic neighbor embedding (t-SNE) 45, a dimensionality reduction algorithm, was used to visualize the distributions of cell types and clusters of immune cells and their marker expressions.

### Quantification of AS Plaque Burden from Coronary Angiography

AS plaque burdens of coronary arteries for individual patients from CAS and ASCVD groups were evaluated and quantified by Gensini Scoring System based on quantitative coronary angiography (QCA) as previously described 46, 47.



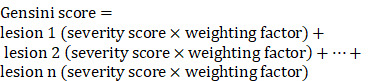



Briefly, the severity scores (1, 2, 4, 8, 16, and 32) indicate the relative reductions of lumen diameters (25%, 50%, 75%, and 100% accordingly) in the coronary artery. And, the weighting factors of different vascular segments are including the main left coronary artery (× 5); the proximal- (× 2.5), middle- (× 1.5), and distal (× 1) segment of the left anterior descending; the first (× 1) and second (× 0.5) diagonal; the proximal- (× 2.5) and distal (× 1) segment of the circumflex branch; the proximal- (× 1), middle- (× 1), and distal (× 1) segment of the right coronary artery. Gensini score evaluation of the patients from CAS and ASCVD groups was performed in a double-blind manner by two investigators.

### Establishment of Disease and Disease Progression Prediction Models

We established two risk prediction models: one (called “Disease Prediction” model; DP model) was used to discriminate non-AS healthy individuals (NC group) from AS patients (CAS and ASCVD groups), and the other (called “Disease Progression Prediction” model; DPP model) was particularly designed to discriminate the low-severity patients (CAS group) from high-severity AS patients (ASCVD group). The model construction mainly consisted of two parts: one was for immune feature selection, and the other was for building up the Random Forest Model.

#### Immune feature selection

To balance the sample sizes across 3 groups, we (i) randomly sampled 25 cases from the pooled CAS and ASCVD groups (n = 70) and used the bootstrap resampling strategy 48 to obtain 25 cases from NC group (n = 13) for DP model; (ii) randomly sampled 15 cases from CAS group (n = 38) and ASCVD group (n = 32) for DPP model. After data normalization, we used Random Forest Model 49 with a 10-fold cross-validation strategy to obtain the average feature importance for each immune cell cluster. We repeated this process for 1,000 times and selected the frequencies of immune cell clusters as the final immune features for modeling as they satisfied the following criteria: (1) when the importance of the feature > 0.04, counted as 1; otherwise, as 0; (2) it had more than 500 times as counted as 1. The finally selected immune features (i) for the DP model are M02, M03, M06, M11, M13, T05, T20, and T23; (ii) for the DPP model are M02, M06, M11, M15, NK04, T05, T15, T17, T20, and T23 **(Figure 5B-C)**. The detailed information and calculated importance of selected immune features, clinical features, and combined features used for model construction are listed in **Table S10**.

#### Modeling process

We randomly selected 70% of samples for training and 30% of samples for testing. Specifically, for the DP model, we randomly sampled 50 cases from AS groups (25 samples each from CAS and ASCVD groups) and 50 cases from NC group (n = 13, using the bootstrap resampling strategy 48) as the training dataset, and the remaining samples (n = 20 for NC group; and n = 20 for CAS and ASCVD groups) were used for testing **(Table S11)**. For the DPP model, we randomly sampled 25 cases respectively from CAS (n = 38) and ASCVD (n = 32) groups as the training dataset, and the remaining samples (n = 13 for CAS group; n = 7 for ASCVD group) were used for testing **(Table S12)**. Using the selected peripheral immune features or clinical features or combined features, the Random Forest Models were trained via 10-fold cross-validation, and the average result of 10 models was considered as the final average model. The testing dataset was used to test the prediction accuracy of the constructed models by receiver operating characteristic curves (ROC). Furthermore, net benefits 50 were also examined to compare the prediction accuracies of DP and DPP models built with different feature sets.

### Statistical Analyses

Clinical variables of three patient groups were either represented as mean ± standard deviation (SD) for continuous variables or as the number (n) and percentage (%) for categorical variables. Chi-squared test was used for statistical analysis of categorical variables and the one-way ANOVA test was used for statistical analysis of continuous variables across 3 groups. All boxplots for comparing the cell frequencies and marker expressions across 3 groups are shown as median ± inter-quartile range (IQR). Linear regression was used for analyzing the Spearman correlation coefficients (*r-value*) and *p*-*values* between the cluster frequencies and clinical characteristics (*e.g*., Gensini scores and serum lipids) across groups. Statistical analysis of each independent experiment was performed with a two-sided Student's t-test with Benjamini-Hochberg adjustment. Adjusted *p* (*p*.*adj*) < 0.05 was considered statistically significant, with *p.*adj* < 0.05, **p.*adj* < 0.01, ***p.*adj* < 0.001, and ****p.*adj* < 0.0001. All statistical analyses were calculated by using R software (version 4.1.1; https://www.r-project.org).

## Results

### Single-cell Immune Atlas of Peripheral Blood in Human Coronary Atherosclerosis

To comprehensively delineate the peripheral immune landscapes and exploit the critical immune biomarkers specifically characterizing the initiation and progression of human CVD, we performed a single-cell CyTOF analysis of CD45^+^ cells derived from PB samples of the enrolled patients and healthy individuals. We obtained 83 PB samples, including 38 for coronary atherosclerosis (CAS) group, 32 for atherosclerotic coronary vascular disease (ASCVD) group, and the other 13 for the non-atherosclerotic healthy control (NC) group. The clinical characteristics of the enrolled individuals were summarized in **Table 1**. Each PB sample was independently stained with two pre-defined antibody-staining panels (T cell panel, **Table S2**; myeloid cell panel, **Table S3**) to interrogate the lymphoid cells and myeloid cells, respectively. And we obtained approximately 8 × 10^7^ CD45**^+^** cells in total, with an average of 1 × 10^6^ cells per sample for each antibody-staining panel, allowing us to implement a thorough characterization of disease-specific immune alternations at the different disease stages.

**Figure 1:**
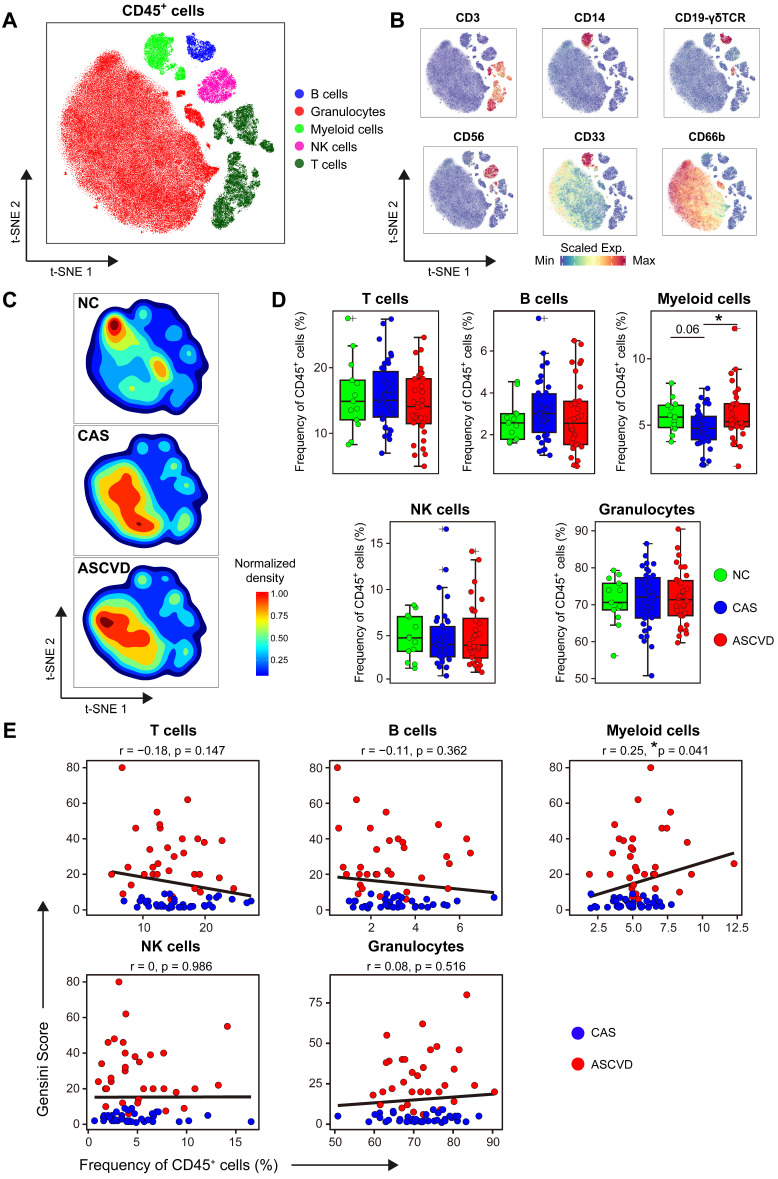
** Immune Landscapes of Peripheral CD45^+^ Cells. (A and B)** t-SNE plots of major immune cell types in CD45^+^ cells from 83 PB samples derived from NC, CAS, and ASCVD groups, analyzed with T cell panel (A) and the normalized expressions of major immune lineage markers (B). **(C)** t-SNE plots of density distributions of CD45^+^ cells across groups, with an equal number (3 × 10^4^) of cells from each group. **(D)** Comparisons of frequencies of major immune cell types in (A) across groups. **(E)** Scatter plots of Pearson's correlation coefficients (*r-value*) between the frequencies of 5 major immune cell types (T cells, B cells, myeloid cells, NK cells, and granulocytes) with Gensini scores in the diseased (CAS and ASCVD) groups. Unpaired Student's t-test with Benjamini-Hochberg adjustment was used in (D), with *p.adj < 0.05. Pearson's correlation coefficients (*r-value*) and *p-value* were labeled in (E).

After data pre-processing, we applied the Phenotyping by Accelerated Refined Community-partitioning (PARC) algorithm 44 to partition CD45^+^ cells into diverse cell clusters for the two antibody-staining panels, respectively (**Figure S1A, S1D; Table S4, S5**). And heterogeneous marker expressions across these identified immune cell clusters were displayed accordingly in t-SNE plots for T cell panel **(Figure S1B-C)** and myeloid cell panel **(Figure S1E-F)**, respectively. We annotated 5 major immune cell types based on the expressions of typical immune lineage markers, including CD3^+^ T cells, CD19^+^ B cells, CD14^+/-^CD33^+^ myeloid cells, CD56^+^ NK cells, and CD45^low^ CD66b^+^ granulocytes **(Figure 1A-B)**. And the frequencies of these major immune cell types for two parallel experiments using two independent antibody-staining panels were highly correlated (*r-values* range from 0.97 to 0.99), demonstrating the high quality and consistency of our CyTOF data **(Figure S1G)**.

The overall distribution patterns of 5 major immune cell types are similar between CAS and ASCVD groups but distinct from NC group as displayed in their density t-SNE plots **(Figure 1C)**. Frequency comparisons of major immune cell types across 3 groups revealed a distinct reduction of myeloid cells in CAS group as compared to NC (*p.adj* = 0.06) and ASCVD (*p.adj* < 0.05) groups **(Figure 1D)**. Moreover, the frequency of myeloid cells shows a significantly positive correlation (*r* = 0.25, *p* = 0.041) with AS plaque burdens of coronary artery (quantified by Gensini score 47, **see Methods**) in diseased (CAS and ASCVD) groups **(Figure 1E)**. These results are well consistent with previous studies 19, 51 and further indicate the critical roles of peripheral myeloid cells in responding to or regulating the initiation and progression of atherogenesis in the human coronary artery. However, we did not observe any significant alternations in the frequencies of other major immune cell types (T cells, B cells, NK cells, and granulocytes) or their clinical associations with the disease conditions, or AS plaque burdens **(Figure 1D-E)**, which may require further in-depth profiling.

### Functional Shifting of Peripheral Myeloid Cells during CVD Development

**Figure 2:**
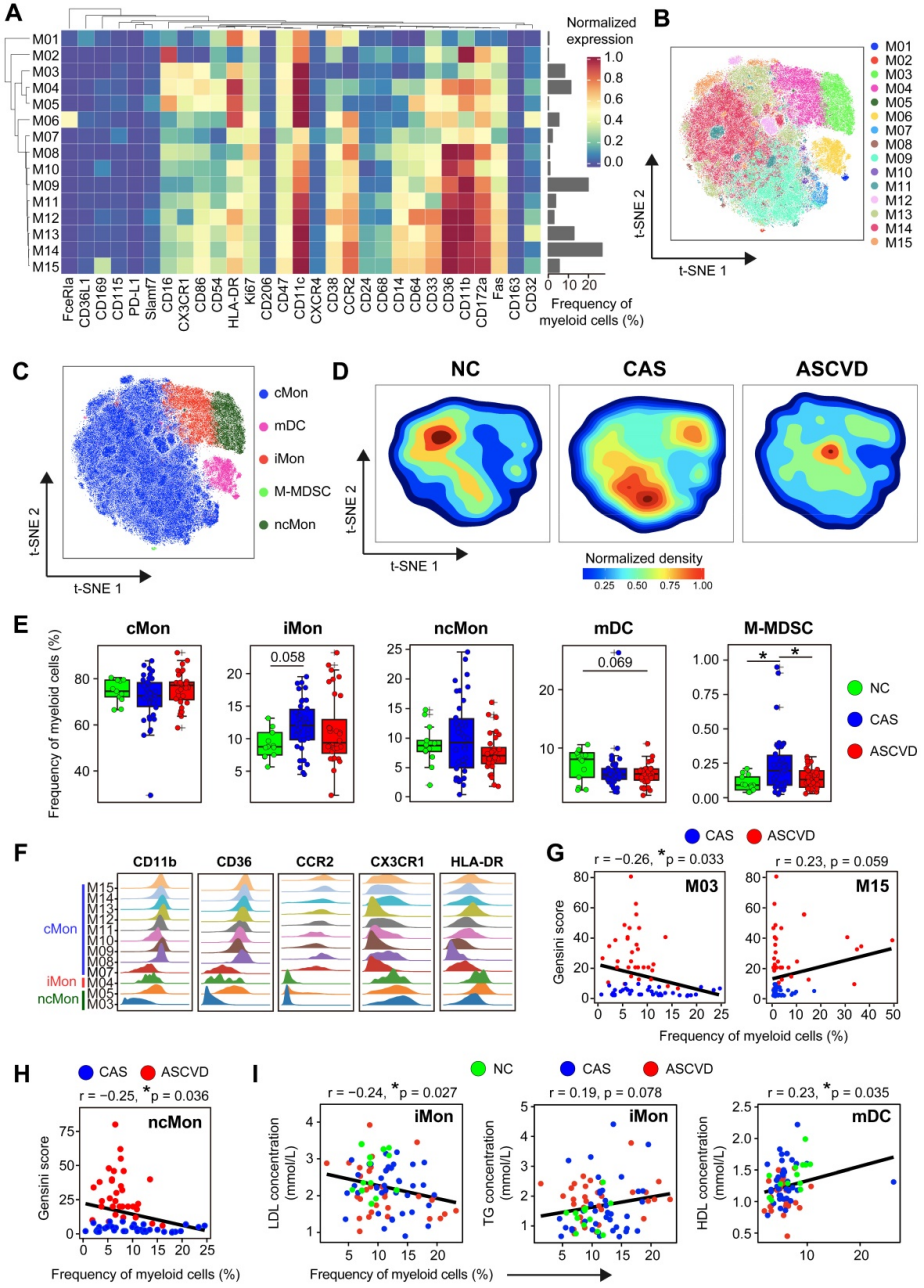
** Heterogeneous Cell Composition and Phenotypes in Peripheral Myeloid Cells. (A)** Heatmap showing the normalized expressions of indicated markers for 15 myeloid cell clusters identified by PARC algorithm by staining with myeloid cell panel. Cluster IDs are labeled on the left and relative frequencies are displayed as a bar graph on the right. **(B and C)** t-SNE plots of myeloid cells, colored by cell clusters (M01-M15) in (B) and major myeloid cell subsets (cMon, iMon, ncMon, mDC, and M-MDSC) in (C). **(D)** t-SNE plots of the density distributions of myeloid cells across groups, with an equal number (3 × 10^4^) of cells from each group.** (E)** Comparisons of frequencies of major myeloid cell subsets across groups. **(F)** Density plots of functional marker expressions on different monocyte clusters (cMon, iMon, and ncMon). **(G and H)** Scatter plots of Pearson's correlation coefficients (*r-value*) between the frequencies of myeloid cell clusters (M03 and M15) (G) and ncMon subset (H) with Gensini scores in the diseased (CAS and ASCVD) groups, respectively. **(I)** Scatter plots of Pearson's correlation coefficient (*r-value*) between the frequencies of major myeloid cell subsets (iMon and mDCs) with serum lipids (*e.g.*, LDL, TG, and HDL) across groups. Unpaired Student's t-test with Benjamini-Hochberg adjustment was used in (E), with *p.adj < 0.05. Pearson's correlation coefficient (*r-value*) and *p-value* were labeled in (G), (H), and (I).

Myeloid cells are the key drivers in innate immunity that initiate and aggravate AS progression 2, 22. To interrogate how their phenotypes and distributions are altered along with AS development in peripheral blood, we grouped myeloid cells and partitioned them into 15 cell clusters with distinct phenotypes by using the PARC algorithm 44 **(Figure 2A-B; Figure S2A-B; Table S6)**. We annotated 9 clusters of classical monocytes (cMon; M07-M15), 1 cluster of intermediate monocytes (iMon; M04), 2 clusters of non-classical monocytes (ncMon; M03 and M05), 2 clusters of myeloid-derived dendric cells (mDC; M01 and M06), and 1 cluster of monocytic myeloid-derived suppressor cells (M-MDSC; M02) **(Figure 2B-C)**.

Distribution comparisons of major myeloid cell subsets revealed their distinct distributions across 3 groups, particularly in cMon and ncMon subsets **(Figure 2C-D)**. cMon subset, the dominant phenotype of monocytes in peripheral blood (~75%), shows no significant frequency changes across groups, whereas the iMon subset slightly increases (by comparing CAS group vs NC group, *p.adj* = 0.058) and then decreases (by comparing ASCVD group vs CAS group) along with AS progression **(Figure 2E)**. Moreover, we also observed a similar alteration pattern (firstly increased and then decreased; *p.adj* < 0.05) along with the disease development in the M-MDSC subset **(Figure 2E)**, which happened to be opposite to the changing trend of overall myeloid cells **(Figure 1D)**, indicating the necessity of detailed characterization of myeloid cell subsets to reveal their heterogeneous alteration patterns. Besides, distinct from other myeloid cell subsets, the mDC subset shows a slightly decreasing trend (by comparing ASCVD group vs NC group, *p.adj* = 0.069) along with AS progression **(Figure 2E)**, again demonstrating the heterogenous phenotypical shifting of myeloid cell subsets as the disease condition progresses from NC to CAS or even ASCVD.

We further compared the frequencies of diverse myeloid cell clusters and found M03 (CCR2^-^CD64^-^ ncMon) slightly decreased (*p.adj* = 0.09) in the advanced stage of disease (lower in ASCVD group than in the other two groups) **(Figure S2C)**. M06 (FceRIa^+^ mDCs) demonstrates a slightly declining trend in the diseased groups (especially ASCVD group; *p.adj* = 0.063) as compared to NC group **(Figure S2C)**, which might be the result of recruiting circulating DC precursors into the inflamed plaques 52. Despite the lower abundance in PB samples, M15 (CD169^+^ cMon) significantly increases in ASCVD group as compared with CAS group **(Figure S2C)**.

Classical monocytes could undergo lineage transitions into non-classical monocytes via intermediate monocytes in multiple inflammatory conditions 53. To explore the dynamic continuum of monocytes, we next compared the expressions of functional molecules on major subsets of monocytes **(Figure 2F)**. Most cMon clusters (M08-M15) highly express molecules related to cell migration (CD11b, CCR2, and CX3CR1) and antigen presentation (CD36 and HLA-DR), except for M07 cluster (CD54^-^ cMon). In contrast, ncMon cells, patrolling along the endothelium during early atherogenesis with anti-inflammatory effects 54, only express high levels of CXC3R1 and HLA-DR but relatively low levels of CCR2, CD36, and CD11b. Besides, the phenotype of iMon subset is somewhere in between cMon and ncMon subsets.

We next analyzed the expressions of functional molecules on myeloid cells across 3 groups to reveal their phenotypic alterations in different disease conditions. We identified significant down-regulation of CD14 expression on mDC subset and CXCR4 on M-MDSC subset in the diseased groups in contrast with NC group, suggesting that these cells could be recruited to pro-inflammatory sites through engaging with macrophage migration-inhibitory factor (MIF) 55 **(Figure S2D)**. CD36L1 (SR-B1), which could inhibit AS progression by mediating cholesterol trafficking and limiting inflammation and oxidation (56), is decreased significantly on cMon subsets in CAS group (*p.adj* < 0.01) and slightly in ASCVD group (*p.adj* = 0.057) in contrast with NC group **(Figure S2E)**. We also noticed that the expressions of CD68 (which can bind and internalize ox-LDL and apoptotic cells 57, 58) and CD32 (FcγRIIA, significantly decreased on peripheral monocytes in AS patients 59) were both significantly (*p.adj* < 0.05) downregulated on ncMon in the diseased groups rather than NC group, accompanied by upregulated CD11b expression in CAS (*p.adj* < 0.05) and ASCVD (*p.adj* = 0.062) groups **(Figure S2F)**. The changes in these critical marker expressions are crucial for regulating monocytes' adhesion to and transmigration across the endothelium into the vascular wall 60. Taken together, peripheral monocytes show the heterogeneous expression patterns of surface and intracellular molecules in different disease conditions, which might enable them to perform distinct pro- or anti-inflammatory functions along with CVD progression.

To delineate the clinical association of myeloid cells in different disease stages, we next examined the correlations between myeloid cell clusters and the Gensini scores. M03 cluster (CCR2^-^CD64^-^ ncMon) is negatively correlated (*r* = -0.26, *p* = 0.033) and M15 cluster (CD169^+^ cMon) is positively correlated (*r* = 0.23, *p* = 0.059) with Gensini scores in the diseased individuals **(Figure 2G)**. Among the major myeloid cell subsets, the ncMon subset is the only subset significantly correlated (*r* = -0.25, *p* = 0.036) with Gensini scores **(Figure 2H and S2G)**. Moreover, we also identified the significant associations between the frequencies of 5 major myeloid cell subsets and serum lipid profiles. iMon subset is negatively correlated with LDL levels (*r* = -0.24, *p* = 0.027) but positively with triglyceride (TG) levels (*r* = 0.19, *p* = 0.078), whereas mDC subset is positively correlated with high-density lipoprotein (HDL) levels (*r* = 0.23, *p* = 0.035) **(Figure 2I)**, indicating the tight relationships between serum lipids and the modulated peripheral myeloid cells 61.

### Phenotypic Alterations of Peripheral T Cells during CVD Development

**Figure 3:**
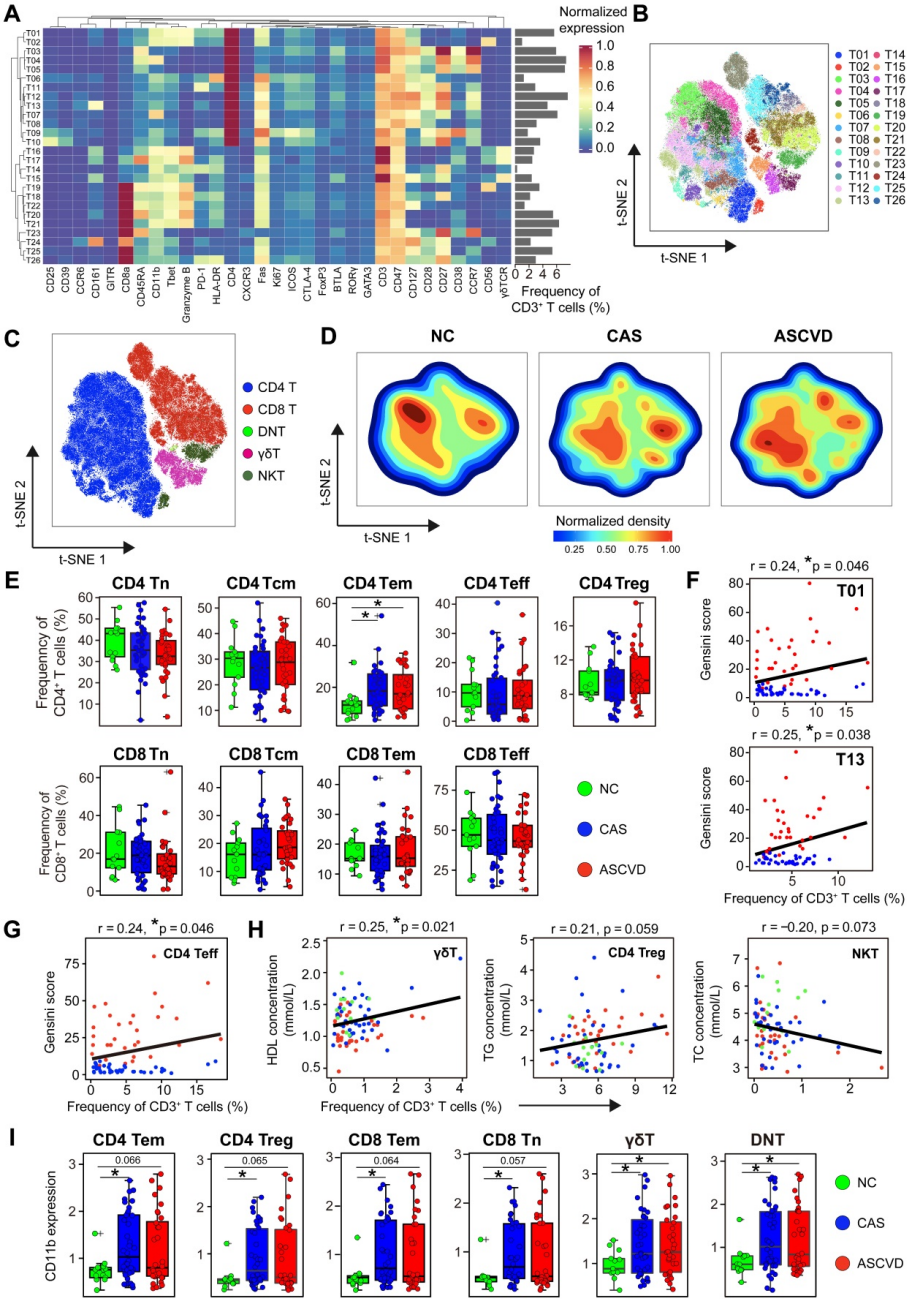
** Heterogeneous Cell Composition and Phenotypes in Peripheral T Cells. (A)** Heatmap showing the normalized expressions of indicated markers for 26 T cell clusters identified by PARC algorithm by staining with T cell panel. Cluster IDs are labeled on the left and relative frequencies are displayed as a bar graph on the right. **(B and C)** t-SNE plots of T cells, colored by cell clusters (B) and major T cell subsets (C).** (D)** t-SNE plots of the density distributions of T cells across groups, with an equal number (3 × 10^4^) of cells from each group.** (E)** Comparisons of frequencies of major subsets of CD4^+^ and CD8^+^ T cells (Tn, Tcm, Tem, Treg, and Teff) across groups. **(F and G)** Scatter plots of Pearson's correlation coefficients (*r-value*) between the frequencies of T cell clusters (T01 and T13) (F) and CD4^+^ Teff (G) with Gensini scores in the diseased (CAS and ASCVD) groups. **(H)** Scatter plots of Pearson's correlations (*r-value*) between the frequencies of major T cell subsets (γδT, CD4^+^ Treg, and NKT) with serum lipids (*e.g.*, HDL, TG, and TC) across groups. **(I)** Comparisons of expression intensities of CD11b on major T cell subsets across groups. Unpaired Student's t-test with Benjamini-Hochberg adjustment was used in (E) and (I), with *p.adj < 0.05. Pearson's correlation coefficient (*r-value*) and *p-value* were labeled in (F), (G), and (H).

T cells are critical participants of adaptive immunity in maintaining the homeostasis of arterial inflammations, and distinct T cell populations in AS plaques have been identified 8, 9. To explore the disease-specific changes of peripheral T cells along with CVD development, we re-clustered CD3^+^ T cells and partitioned them into 26 distinct cell clusters, which included 13 CD4^+^ T (T01-T13), 9 CD8^+^ T (T18-T26), 3 γδT (T15-T17), and 1 double-negative T (DNT; T14) cell clusters **(Figure 3A-B; Figure S3A-B; Table S7)**. We noticed a similar distribution pattern (although with subtle differences) of T cells between CAS and ASCVD groups, both of which were distinct from NC group **(Figure 3C-D)**. Despite this, we did not identify any significant changes in the frequencies of major T cell subsets (*e.g.*, CD4^+^ T, CD8^+^ T, γδT, and NKT) across 3 groups **(Figure S3C)**.

We next compared the distributions of major functional T cell subsets annotated by typical lineage markers across groups and found a significant increase of CD4^+^ effector memory T (Tem) cells in the diseased (CAS and ASCVD) groups compared to NC group (**Figure 3E**). However, we did not identify any significant frequency changes in individual T cell clusters (T01-T26) **(Data not shown)**. By examining how these periphery T cell clusters were relevant to AS plaque burdens of coronary arteries (Gensini scores), we found that T01 (co-expressed Granzyme B and T-bet; Th1), T13 (co-expressed CD161; Th17), and CD4^+^ Teff were positively correlated with Gensini scores in the diseased individuals (T01: *r* = 0.24, *p* = 0.046; T13: *r* = 0.25, *p* = 0.038; CD4^+^ Teff: *r* = 0.24, *p* = 0.046) **(Figure 3F-G)**. Notably, we also identified the clinical associations between the frequencies of peripheral T cell subsets and serum lipid profiles, including the positive correlation between γδT and HDL levels (*r* = 0.25, *p* = 0.021), the relatively weaker positive correlation between CD4^+^ Treg and total cholesterol (TC) levels (*r* = 0.21, *p* = 0.059), and the negative correlation between NKT and TG levels (*r* = -0.20, *p* = 0.073) **(Figure 3H)**. Herein, these results together indicate that serum lipids are also closely related to the alterations of peripheral T-cell distribution in both disease initiation and progression, suggesting the potential bridging of lipid metabolism and peripheral immune system to coordinately regulate the atherogenesis of human coronary arteries.

To reveal the functional modulations of T cells during human coronary atherogenesis, we then analyzed the expressions of functional molecules on T cells. CD11b expression is significantly upregulated on CD4^+^ and CD8^+^ T cell subsets (*e.g.*, Tn, Tem, and Treg) as well as γδT and DNT cells in the diseased groups **(Figure 3I)**. Moreover, we noticed that PD-1 (programmed cell death protein 1) expression on CD4^+^ Tem cells but not on CD8^+^ Tem cells was significantly declined in the diseased groups compared to NC group **(Figure S3D-E)**. Besides, the expression of HLA-DR declines on CD4^+^ Teff in CAS (*p.adj* < 0.05) and ASCVD (*p.adj* = 0.068) groups in comparison to NC group, whereas significantly increases on CD4^+^ Treg in ASCVD group rather than CAS group **(Figure S3D)**. Similar expression patterns exist for HLA-DR on CD8^+^ Tem and CD8^+^ Teff as well. As the minor T cell populations in peripheral blood, DNT, γδT, and NKT cells experience a similar phenotypical remodeling along with disease progression, with the significantly decreased expression of HLA-DR in the diseased groups as compared to NC group **(Figure S3F)**.

Taken together, we have not only identified subtle but significant changes in peripheral T-cell composition and the functional molecule expressions on T cells in the diseased groups but also demonstrated some of these frequency or phenotypic changes are closely associated with different disease conditions or clinical characteristics.

### Distinct Modulation in Circulating B and NK Cells during CVD Development

**Figure 4:**
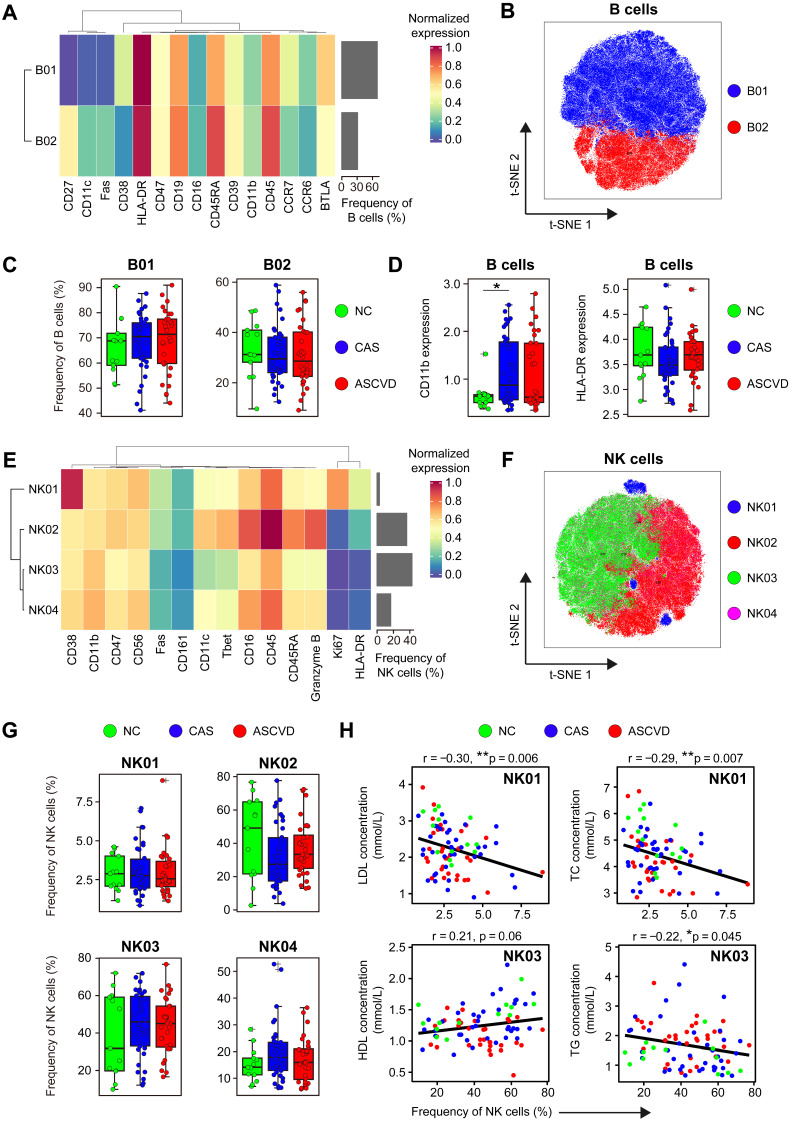
** Immune Profiling of Peripheral B and NK Cells. (A and E)** Heatmaps showing the normalized expressions of indicated markers on 2 B cell clusters (A) and 4 NK cell clusters (E) by staining with T cell panel by the PARC algorithm. Cluster IDs are labeled on the left and relative frequencies are displayed as a bar graph on the right. **(B and F)** t-SNE plots of B cells (B) and NK cells (F), colored by cell clusters. **(C and G)** Comparisons of the frequencies of B cell clusters (C) and NK cell clusters (G) across groups. **(D)** Comparisons of expression intensities of the functional markers (CD11b and HLA-DR) on B cells. **(H)** Scatter plots of Pearson's correlation coefficient (*r-value*) between the frequencies of NK cell clusters (NK01 and NK03) with serum lipids (*e.g.*, HDL, LDL, TG, and TC) across groups. Unpaired Student's t-test with Benjamini-Hochberg adjustment was used in (C), (D), and (G), with *p.adj < 0.05. Pearson's correlation coefficient (*r-value*) and *p-value* were labeled in (H).

To explore the disease-specific changes in the compositions and phenotypes of B cells during disease development, we further analyzed two B cell clusters (C34 and C35) identified by clustering CD45^+^ cells with T cell panel **(Figure S1A-B)**. By examining the distributions of B01 (CD27^-^ CD38^+^; C34 in **Figure S1A**) and B02 (CD27^+^ CD38^-^; C35 in **Figure S1A**) across 3 groups **(Figure 4A-B; Figure S4A-B; Table S8)**, we neither found significant changes in their frequencies nor their clinical correlations with AS plaque burdens **(Figure 4C and S4C)**. We only identified the significantly higher expression of CD11b in the initiation phase of the disease (by comparing NC group with CAS group), and no other significant changes exist for the functional molecule expressions (*e.g.*, HLA-DR) across 3 groups **(Figure 4D)**.

NK cells are important cytotoxic lymphocytes in innate immunity, which promote the development of AS plaque lesions by secreting Granzyme B and perforin in the local inflamed lesions 62. However, the phenotypes and roles of peripheral NK cells in human coronary atherogenesis are unclear. We then analyzed NK cells (C25, C26, C27, and C28) by using the CD45^+^ cell clustering results with T cell panel **(Figure S1A-B)**. Four distinct NK cell clusters (NK01-NK04) were identified, among which NK02 (Granzyme B^++^ CD45RARA^++^) and NK03 (CD11c^dim^ T-bet^dim^) were the dominant phenotypes in peripheral blood, whereas NK01 (CD38^++^) and NK04 (CD11^+^ T-bet^+^ HLA-DR^-^) were the minor subtypes **(Figure 4E-F; Figure S4D-E; Table S9)**. Although the frequencies of NK cell clusters do not statistically vary along with the disease progression **(Figure 4G)**, they show significant associations with serum lipid profiles, including the negative correlations of NK01 with LDL levels (*r* = -0.30, *p* = 0.006) and TC levels (*r* = -0.29, *p* = 0.007), the positive correlation between NK03 and HDL levels (*r* = 0.21, *p* = 0.06), and the negative correlation between NK03 and TG levels (*r* = -0.22, *p* = 0.045) **(Figure 4H)**. However, neither NK cell clusters exhibit any clinical correlations with Gensini scores **(Figure S4F)**, nor do the expressions of cytotoxic-related molecules (*e.g.*, Fas and Granzyme B) are significantly changed across 3 groups **(Figure S4G)**.

### Distinct Immune Cell Interactions Among CAS and ASCVD Groups

**Figure 5:**
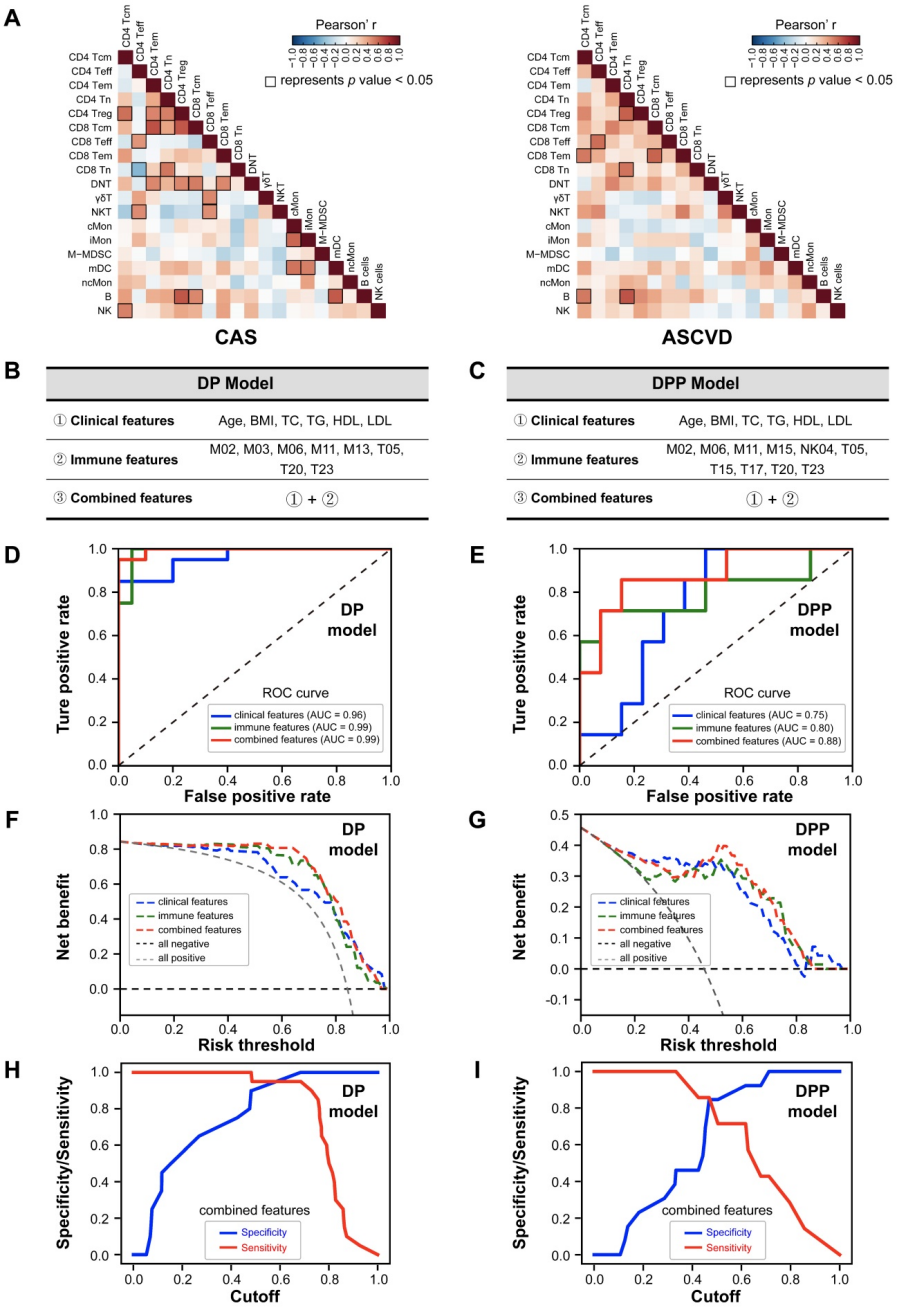
** Immune-signature-based CVD Risk Prediction Model. (A)** Heatmap showing the Pearson's correlation coefficients for relationships between major immune cell subsets in CAS (left) and ASCVD (right) groups. **(B and C)** The selected variables of clinical features, immune features, and combined features are used for building the disease prediction (DP) model (B) and disease progression prediction (DPP) model (C).** (D and E)** The receiver operating characteristic curves (ROC) of DP model (D) and DPP model (E), colored by the testing datasets with different feature sets. **(F and G)** The decision curve analysis (DCA) for comparing the net benefits of DP model (F) and DPP (G) model, colored by the testing datasets with different feature sets, and the additional “all negative” and “all positive” lines represent the net benefit of none of diseased AS patients or all diseased AS patients, respectively. **(H and I)** The sensitivity and specificity comparisons of DP model (H) and DPP model (I) that both built with combined features at different cutoffs. Pearson's correlation analysis was used in (A), and the black square box represents *p* < 0.05. AUC scores were labeled in (D) and (E).

To comprehensively explore the potential immune cell interactions between major peripheral immune cell subsets (including T cells, B cells, myeloid cells, and NK cells) among the diseased (CAS and ASCVD) groups, we calculated the Spearman correlations of cell frequencies between the identified immune cell subsets and revealed 22 pairs of the significant correlations in CAS group **(Figure 5A**, left panel; **Figure S5A)**, and 7 pairs in ASCVD group **(Figure 5A**, right panel; **Figure S5B)**. Specifically, we identified significant and positive correlations between CD4^+^ Tn and CD8^+^ Tn cells, CD4^+^ Tn and CD4^+^ Treg cells, and CD4^+^ Teff and CD8^+^ Teff cells both existed in CAS and ASCVD groups. Comparatively, the correlations we found in ASCVD groups mainly existed between the major subsets of lymphoid cells, *e.g.*, CD4^+^ T, CD8^+^ T, and B cells **(Figure S5B)**. Whereas the immune cell interactions found in CAS group are more complex and diverse, including the interactions between the functional subsets of myeloid cells (*e.g.*, cMon, iMon, and mDC) as well as the cross-talks between the myeloid cell subsets and lymphoid cell subsets **(Figure S5A)**. Taken together, these results revealed the distinct but complex immune cell interactions during the early stage (CAS) and advanced stage (ASCVD) of atherogenesis in human coronary arteries, suggesting heterogeneous immune regulations and disease-related signatures can be fully captured in peripheral blood.

### Establishment of Immune-signature-based CVD Risk Prediction Models

The peripheral immune atlas of human coronary atherosclerosis reveals the complex and distinct immune alternations at different disease stages, which enable us to utilize these peripheral immune features for discriminating individuals in different groups. We built two risk prediction models by using the Random Forest algorithm 49 (**see Methods**), including the disease prediction (DP) model and the disease progression prediction (DPP) model. DP model is used to discriminate non-AS individuals (NC group) from AS patients (CAS and ASCVD groups), and the DPP model is designed to particularly separate the low-severity patients (CAS group) from the high-severity AS patients (ASCVD group). As the sample size (n = 13) in NC group was much fewer than the other two groups, we adopted a bootstrap resampling strategy 63 to boost the sample size of NC group to a similar level as the total size of CAS and ASCVD groups (**see Methods**).

We used the selected immune features (represented by the relative frequencies of immune cell clusters) for the DP model (*i.e.,* M02, M03, M06, M11, M13, T05, T20, and T23) and DPP model (*i.e.,* M02, M06, M11, M15, NK04, T05, T15, T17, T20, and T23), and integrated them with the clinical features (age, BMI, TC, TG, HDL, and LDL) as the combined features **(Figure 5B-C; Table S10)**. We randomly selected 70% of the samples as the training dataset, and the left 30% of samples as the testing dataset to evaluate the performance of DP model **(Table S11)** and DPP model **(Table S12)**. We then applied 10-fold cross-validation to train the DP and DPP models that were built with either immune features, clinical features, or combined features **(Figure S6A-F)**.

The averaged receiver operating characteristic curves (ROC) for prediction models with different feature sets were generated using the testing datasets **(Figure 5D-E)**. Comparing these averaged ROC curves, we observed the superior performance of using the combined features than using one kind of feature set (clinical or immune features) alone. On the testing dataset (including 40 samples for DP model, and 20 samples for DPP model), the predictions combined features can achieve high accuracy for both DP model (AUC = 0.99) and DPP model (AUC = 0.88) **(Figure 5D-E)**. The predictions using immune features alone also exhibited superior performances (AUC = 0.99 for DP model; AUC = 0.80 for DPP model) than the one using clinical features alone (AUC = 0.96 for DP model; AUC = 0.75 for DPP model), strongly indicating that peripheral immune features were valuable traits for CVD risk prediction. Further, we compared the net benefits 50 of our prediction models built with different feature sets and confirmed that the models built with combined features also achieved the highest net benefits, followed by the models built with immune features alone **(Figure 5F-G)**, consistent with the ROC analysis. Of note, the specificity and sensitivity analysis of DP and DPP models that are built with combined features showed varied cutoffs, among which 0.6 for the DP model and 0.5 for the DPP model appeared as the best cutoff, respectively **(Figure 5H-I)**.

## Discussion

Atherosclerosis is a chronic and lipid-driven inflammatory disease of the arterial intima, and atherosclerotic cardiovascular diseases (ASCVD) have become a global concern. Recently, the Canakinumab Anti-inflammatory Thrombosis Outcomes Study (CANTOS) has confirmed that anti-inflammatory therapies targeting the NLRP3 inflammasome to IL-1 to IL-6 to CRP signaling pathway could benefit AS patients for the lower CVD risks 64-66, indicating the feasibility of anti-inflammatory therapy as a novel therapeutic approach to combat atherosclerosis 67, 68. Here, we comprehensively delineated the peripheral immune atlas of human CAS and ASCVD, and utilized the periphery immune features as liquid indicators for CVD risk prediction models, providing a deep insight into the early detection and long-term management of disease progression at the molecule level.

Our study has revealed the significant changes in peripheral myeloid cells in different stages of human coronary atherosclerosis. These disease-specific changes are reflected not only in the composition of total myeloid cells in CD45^+^ cells **(Figure 1D)** but also in the frequencies of myeloid cell subsets (M-MDSC and CD169^+^ cMon) **(Figure 2E and S2C)**. iMon subset is functionally linked with neo-vascularization in the advanced AS plaques by producing inflammatory cytokines, *i.e.*, IL-1β, IL-6, and TNF-α 69, and previous studies also have confirmed the important roles of serum LDL and TC-rich lipoproteins in driving human atherosclerosis 70, 71. Our findings reveal that iMon subset negatively correlates with serum LDL levels, suggesting the potential transmigration of iMon subsets from peripheral blood into the inflamed plaques after exposure to athero-antigens (*e.g.*, ox-LDL). M-MDSC subset is a distinct population of myeloid cells that performs immunosuppressive functions by inhibiting the activation of T and myeloid cells 72. The significant increase of M-MDSC particularly in CAS group indicates they might swiftly be recruited from bone marrow and accumulated in peripheral blood in response to the artery inflammations once the disease initiates 73. Moreover, we also found some of these changes significantly correlated with clinical characteristics, *e.g.*, Gensini scores and serum lipids **(Figure 2G-I; Figure S2G)**. These results together demonstrate the heterogenous alternations of peripheral myeloid cells along with disease development, which can be precisely detected by single-cell analyses once the disease initiates.

T cells, as the dominant immune cell type infiltrating human AS plaques 9, are the key modulators in the formation and maturation of AS plaque lesions. In this study, we revealed subtle but significant changes in peripheral T cells in different disease conditions, including the continuous increase of CD4^+^ Tem cells from NC group to the diseased groups **(Figure 3E)**. In atherosclerosis, diverse CD4^+^ T cell subsets (*e.g.*, Th1, Th2, and Th17) influence AS progression either by activating or suppressing the immune system or by interacting with B cells to secret antibodies 74. Beyond the distinct changes in the frequencies, we also observed significant alterations in the expressions of functional molecules (*e.g.*, CD11b, HLA-DR, and PD-1) on major T cell subsets along with the initiation and progression of disease **(Figure 3I; Figure S3D-F)**, reflecting T-cell activation and remodeling in periphery blood during human coronary atherogenesis. Positive correlations between the frequencies of T cell clusters (T01, T13, and CD4^+^ Teff) and Gensini scores **(Figure 3F-G)** demonstrate their tight connections to clinical characteristics, suggesting the pro-atherogenic roles of these T cells in AS progression are not only restricted within AS plaques 23, 75 but also reflected in peripheral blood. Further, our findings also reveal the close associations between serum lipids (*e.g.*, HDL, TC, and TG) and specific T cell subsets (*e.g.*, γδT, Treg, and NKT) **(Figure 3H)**. These results together have demonstrated that once atherogenesis initiates, peripheral T cells are tightly involved and continuously changed along with the disease development, and more importantly, these disease-specific changes can be fully captured via single-cell analyses of peripheral immune cells.

Coronary computed tomography angiography (CCTA) is becoming the first-line and non-invasive test for examining and diagnosing patients with CAS and ASCVD. It could characterize coronary AS plaques, evaluate patients' risks of future clinical events, assess coronary artery stenosis, and infer the presence of ischemia from functional modeling 76. However, patients who are allergic to contrast agents or with severe renal function damage are the contraindications for CCTA, and its evaluation efficacy is significantly restrained in patients with severe coronary calcification and arrhythmia. To enhance the prediction accuracy of CVD risk, CCTA is still required to identify biological procedures that drive AS progression, such as inflammation. Therefore, more molecular biomarkers and their underlying biological process are critical for predicting the progression of AS and the risk of CVD events, including systemic inflammatory responses. However, compared with CCTA, there is no absolute contraindication for individuals to detect their periphery immune features in blood samples by high-dimensional CyTOF analysis, and it comprehensively reflects the disease-specific inflammatory responses which could commendably fill up the gap in CCTA. Although the immune systems and inflammations are tightly involved in the pathogenesis of CVD, peripheral immune features have not yet been considered in the existing CVD risk prediction models 36. Here, we have established two disease prediction models (DP and DPP models) for discriminating the patients in different disease conditions and revealed that integrating the immune and clinical features (as the combined features) could achieve superior prediction accuracy **(Figure 5D-G)** with AUC = 0.99 for DP model and AUC = 0.88 for DPP model, demonstrating the crucial necessity of using peripheral immune features as valuable traits for CVD risk prediction.

Our study still has some limitations. First, the relatively small cohort sample size in the current study might lead to a slight overestimation of the performance of the constructed DP model. Larger independent human cohorts are required in the future to further validate the prediction performance of our proposed models. Second, our study is focusing on the disease-specific changes in the compositions and phenotypes of peripheral immune cells at the single-cell protein level, whereas the exact biological functionalities of these immune cells and their contributions to disease development are not determined yet, and thus future studies on their regulation mechanisms are worthy of further exploration.

## Conclusion

In conclusion, we have systematically characterized the peripheral immune atlas of patients with CAS and ASCVD and identified the significant changes of peripheral immune cell subsets on both cell distributions and the functional marker expressions for individuals in different disease conditions. With these newly identified immune features in peripheral blood, we established CVD risk prediction models that could effectively predict the presence and severity of coronary atherosclerosis in humans. Therefore, our findings together have pointed out an applicable and minimally invasive liquid immune biomarker that could potentially serve as a novel and potent assessment tool for the early detection and long-term monitoring of coronary AS development and also provided further insights into the immune-targeted therapies for the management of CVD patients.

## Supplementary Material

Supplementary figures and tables.

## Figures and Tables

**Table 1:** Demographic and Clinical Features of the Cohorts

Parameters	NC (n=13)	CAS (n=38)	ASCVD (n=32)	*p*-value
**Basic characteristics**				
Age (years)	56.38 ± 5.04	56.50 ± 7.94	60.03 ± 7.59	0.111
Male, n (%)	6 (46.2)	20 (52.6)	23 (71.9)	0.156
BMI (kg/m^2^)	23.78 ± 2.98	23.48 ± 3.21	25.35 ± 3.63	0.053
Ever smoker, n (%)	3 (23.1)	13 (34.2)	18 (56.3)	0.063
Hypertension, n (%)	2 (15.4)	15 (39.5)	22 (68.8)	0.002
Systolic pressure (mmHg)	118.80 ± 11.74	127.00 ± 18.20	133.10 ± 20.31	0.058
Diastolic pressure (mmHg)	74.77 ± 7.80	76.65 ± 10.20	80.83 ± 8.20	0.069
Hyperlipidemia, n (%)	2 (15.4)	14 (36.8)	21 (65.6)	0.004
Diabetes, n (%)	0 (0)	5 (13.2)	4 (12.5)	0.390
**Laboratory examinations**				
CK (U/L)	94.56 ± 38.85	72.87 ± 28.82	103.5 ± 42.72	0.003
CK-MB (U/L)	16.14 ± 8.47	9.66 ± 5.14	13.13 ± 6.71	0.004
LDH (U/L)	185.80 ± 28.34	177.70 ± 43.76	176.40 ± 29.23	0.724
FBG (mmol/L)	6.41 ± 1.06	5.09 ± 0.94	5.96 ± 2.05	0.009
UA (μmol/L)	324.10 ± 75.19	379.30 ± 121.40	384.40 ± 79.04	0.168
AST (U/L)	19.81 ± 10.23	22.34 ± 17.72	28.01 ± 18.53	0.244
ALT (U/L)	28.17 ± 6.11	27.84 ± 12.22	30.06 ± 10.19	0.675
Cre (mg/dL)	62.77 ± 15.25	61.97 ± 13.10	74.28 ± 17.55	0.003
WBC (×10^9^/L)	5.94 ± 1.73	6.27 ± 1.54	6.48 ± 1.63	0.587
Neutrophils (× 10^9^/L)	3.67 ± 1.23	3.87 ± 1.18	4.10 ± 1.30	0.535
Lymphocytes (× 10^9^/L)	1.66 ± 0.41	1.76 ± 0.49	1.74 ± 0.54	0.796
Monocytes (× 10^9^/L)	0.46 ± 0.21	0.45 ± 0.15	0.45 ± 0.14	0.954
Eosinophil (× 10^9^/L)	0.13 ± 0.14	0.16 ± 0.17	0.15 ± 0.11	0.828
Basophil (× 10^9^/L)	0.04 ± 0.03	0.03 ± 0.01	0.03 ± 0.01	0.131
TC (mmol/L)	5.02 ± 0.90	4.40 ± 0.77	4.22 ± 0.96	0.022
TG (mmol/L)	1.35 ± 0.48	1.62 ± 0.91	1.87 ± 0.65	0.110
HDL-C (mmol/L)	1.40 ± 0.26	1.31 ± 0.30	1.10 ± 0.24	<0.001
LDL-C (mmol/L)	2.60 ± 0.57	2.19 ± 0.61	2.14 ± 0.67	0.074
FFA (μmol/L)	238.00 ± 90.68	315.50 ±167.30	445.10 ± 169.50	<0.001
**Medications**				
Statins, n (%)	0 (0)	12 (31.6)	19 (59.4)	<0.001
Aspirin, n (%)	0 (0)	7 (18.4)	18 (56.3)	<0.001
β-blocker, n (%)	0 (0)	5 (13.2)	14 (43.8)	0.001
Ca^2+^ blocker, n (%)	0 (0)	7 (18.4)	13 (40.6)	0.008
Clopidrogrel/Plasugrel, n (%)	2 (15.4)	9 (23.7)	10 (31.3)	0.515
Gensini Score	0	3.71 ± 2.30	29.05 ± 17.10	<0.001

Categorical variables are shown as number (n) and frequency (%), and continuous variables are shown as mean ± SD. Chi-squared test and one-way ANOVA test are used for statistical analysis.

## Abbreviations

CVD: cardiovascular disease
AS: atherosclerosis
CyTOF: cytometry by time-of-flight
PB: peripheral blood
NC: non-atherosclerotic healthy control
CAS: coronary atherosclerosis
ASCVD: atherosclerotic cardiovascular disease
CRP: C-reactive protein
IL: interleukin
Tn: naïve T
Tcm: central memory T
Tem: effector memory T
Teff: effector T
Treg: regulatory T
NK: natural killer
DNT: double negative T
NKT: natural killer T
CSB: cell staining buffer
PARC: phenotyping by accelerated refined community-partitioning
t-SNE: t-distributed stochastic neighbor embedding
QCA: quantitative coronary angiography
mDC: myeloid-derived dendric cell
M-MDSC: monocytic myeloid-derived suppressor cells
PD-1: programmed cell death protein 1
BMI: body mass index
CK: creatine kinase
CK-MB: creatine kinase isoenzyme
LDH: lactate dehydrogenase
FBG: fasting blood glucose
UA: uric acid
AST: aspartate aminotransferase
ALT: alanine aminotransferase
Cre: creatinine
WBC: white blood cell
TC: total cholesterol
TG: triglyceride
HDL: high-density lipoprotein
LDL: low-density lipoprotein
FFA: free fatty acids
ROC: receiver operating characteristic curves
MACE: major adverse cardiovascular events
CCTA: coronary computed tomography angiography
