# Supplementary material for: High-dimensional Single-cell Analysis Delineates Peripheral Immune Signature of Coronary Atherosclerosis in Human Blood
Source: Theranostics. 2022 Sep 21;12(15):6809–25. doi: 10.7150/thno.73336 (PMC9516242; doi:10.7150/thno.73336)
Supplement: Supplementary file 1 — Supplementary figures and tables. [file thnov12p6809s1.pdf]

Figure S1. Two Independent Panels for Immune Profiling of Peripheral CD45$^+$ Cells. (A and D) Heatmaps showing the normalized expressions of indicated markers on CD45$^+$ cell clusters by staining with T cell panel (A) and myeloid cell panel (D) with PARC algorithm, respectively. **(B and E)** t-SNE plots of CD45$^+$ cells, colored by cell clusters identified by T cell panel (B) and myeloid cell panel (E), as in (A) and (D). **(C and F)** t-SNE plots of the normalized expressions of markers on CD45$^+$ cells with T cell panel (C) and myeloid cell panel (F). **(G)** Scatter plots of Pearson's correlation coefficient (*r-value*) between the frequencies of 5 major immune cell types identified by T cell panel and myeloid cell panel, respectively. Pearson's correlation coefficient (*r-value*) and *p-value* were labeled in (G).

Figure S2. Compositions and Phenotypes of Peripheral Myeloid Cells. (A) t-SNE plots of the normalized expressions of indicated markers on myeloid cells by staining with myeloid cell panel. **(B)** t-SNE plots of the cells in the designated myeloid cell clusters (M01-M15). **(C)** Comparisons of the frequencies of myeloid cell clusters (M03, M06, and M15) across groups. **(D, E, and F)** Comparisons of the expression intensities of functional molecules on mDC and M-MDSC subsets (D), cMon subset (E), and ncMon subset (F) across groups. **(G)** Scatter plots of Pearson's correlations (*r-value*) between the frequencies of major myeloid cell subsets (cMon, iMon, mDC, and M-MDSC) with Gensini scores in the diseased (CAS and ASCVD) groups. Unpaired Student's t-test with Benjamini-Hochberg adjustment was used in (C), (D), (E), and (F), with **p.adj* < 0.05 and ***p.adj* < 0.01. Pearson's correlation coefficient (*r-value*) and *p-value* were labeled in (G).

Figure S3. Compositions and Phenotypes of Peripheral T Cells. (A) t-SNE plots of the normalized expression of indicated markers on T cells by staining with T cell panel. **(B)** t-SNE plots of the cells in the designated T cell clusters (T01-T26). **(C)** Comparisons of the frequencies of major T cell subsets (CD4$^+$ T, CD8$^+$ T, DNT, γδT, and NKT) across groups. **(D, E, and F)** Comparisons of the expression intensities of functional molecules on major subsets of CD4$^+$ T (D), CD8$^+$ T (E), and DNT, γδT, and NKT cells (F) across groups. Unpaired Student's t-test with Benjamini-Hochberg adjustment was used in (C), (D), (E), and (F), with **p.adj* < 0.05, ***p.adj* < 0.01, and ****p.adj* < 0.001.

Figure S4. Compositions and Phenotypes of Peripheral B and NK Cells. (A and D) t-SNE plots of the normalized expressions of indicated markers on B cells (A) and NK cells (D), as in (Figure 4B and 4F). **(B and E)** t-SNE plots of cells in the designated B cell clusters (B) and NK cell clusters (E). **(C and F)** Scatter plots of Pearson's correlation coefficients (*r-value*) between the frequencies of B cell clusters (C) and NK cell clusters (F) with Gensini scores in the diseased (CAS and ASCVD) groups. **(G)** Comparisons of expression intensities of functional markers (Fas and Granzyme B) on NK cells. Unpaired student's t-test with Benjamini-Hochberg

adjustment was used in (G), and *$p.adj < 0.05$. Pearson's correlation coefficient (*r-value*) and *p-value* were labeled in (C) and (F).

Figure S5. Interactions of Peripheral Immune Cells in the Diseased Patients. (A and B) Scatter plots of Pearson's correlation coefficients (*r-value*) between the frequencies of major immune cell subsets in CAS patients (A) and ASCVD patients (B). Pearson's correlation coefficients (*r-value*) and *p-value* were labeled in (A) and (B).

Figure S6. Training and Testing Datasets of CVD Risk Prediction Models. (A and B) The classification results of the training dataset (left), testing dataset (middle), and all sample datasets (right) for DP (A) and DPP (B) models were built with combined features. **(C and D)** The classification results of the training dataset (left), testing dataset (middle), and all sample datasets (right) for DP (C) and DPP (D) models were built with immune features alone. **(E and F)** The classification results of the training dataset (left), testing dataset (middle), and all sample datasets (right) for DP (E) and DPP (F) models were built with clinical features alone. Specificity and sensitivity scores of each trained DP and DPP model were labeled in (A to F).

Figure S1

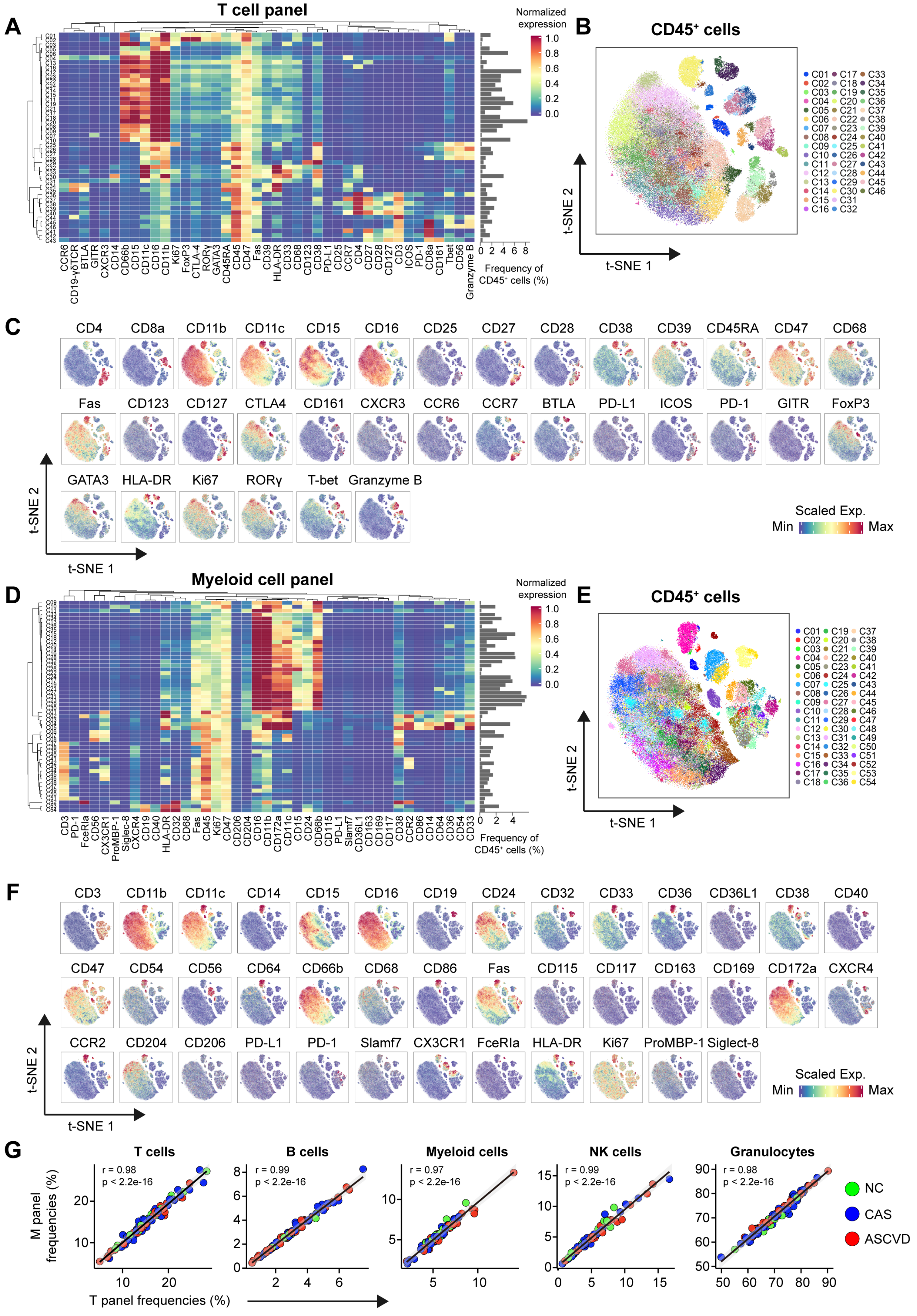
A
T cell panel
Normalized expression
1.0
0.8
0.6
0.4
0.2
0.0
CCR6
CD19-γδTCR
BTLA
GITR
CXCR3
CD14
CD66b
CD15
CD11c
CD16
CD11b
Ki67
FoxP3
CTLA-4
RORγ
GATA3
CD45RA
CD45
CD47
Fas
CD39
HLA-DR
CD33
CD68
CD123
CD38
PD-L1
CD25
CCR7
CD4
CD27
CD28
CD127
CD3
ICOS
PD-1
CD8a
CD161
Tbet
CD56
Granzyme B
0 2 4 6 8
Frequency of CD45⁺ cells (%)
B
CD45⁺ cells
t-SNE 2
t-SNE 1
C01 C02 C03 C04 C05 C06 C07 C08 C09 C10 C11 C12 C13 C14 C15 C16
C17 C18 C19 C20 C21 C22 C23 C24 C25 C26 C27 C28 C29 C30 C31 C32
C33 C34 C35 C36 C37 C38 C39 C40 C41 C42 C43 C44 C45 C46
C
CD4 CD8a CD11b CD11c CD15 CD16 CD25 CD27 CD28 CD38 CD39 CD45RA CD47 CD68
Fas CD123 CD127 CTLA4 CD161 CXCR3 CCR6 CCR7 BTLA PD-L1 ICOS PD-1 GITR FoxP3
GATA3 HLA-DR Ki67 RORγ T-bet Granzyme B
t-SNE 2
t-SNE 1
Scaled Exp.
Min
Max
D
Myeloid cell panel
Normalized expression
1.0
0.8
0.6
0.4
0.2
0.0
CD3
PD-1
FceRIa
CD56
CX3CR1
ProMBP-1
Siglec-8
CXCR4
CD19
CD40
HLA-DR
CD32
CD68
Fas
CD45
Ki67
CD47
CD206
CD204
CD16
CD11b
CD172a
CD11c
CD15
CD24
CD66b
CD115
PD-L1
Slamf7
CD36L1
CD163
CD169
CD117
CD38
CCR2
CD86
CD14
CD64
CD36
CD54
CD33
0 2 4
Frequency of CD45⁺ cells (%)
E
CD45⁺ cells
t-SNE 2
t-SNE 1
C01 C02 C03 C04 C05 C06 C07 C08 C09 C10 C11 C12 C13 C14 C15 C16 C17 C18
C19 C20 C21 C22 C23 C24 C25 C26 C27 C28 C29 C30 C31 C32 C33 C34 C35 C36
C37 C38 C39 C40 C41 C42 C43 C44 C45 C46 C47 C48 C49 C50 C51 C52 C53 C54
F
CD3 CD11b CD11c CD14 CD15 CD16 CD19 CD24 CD32 CD33 CD36 CD36L1 CD38 CD40
CD47 CD54 CD56 CD64 CD66b CD68 CD86 Fas CD115 CD117 CD163 CD169 CD172a CXCR4
CCR2 CD204 CD206 PD-L1 PD-1 Slamf7 CX3CR1 FceRIa HLA-DR Ki67 ProMBP-1 Siglect-8
t-SNE 2
t-SNE 1
Scaled Exp.
Min
Max
G
T cells
r = 0.98
p < 2.2e-16
B cells
r = 0.99
p < 2.2e-16
Myeloid cells
r = 0.97
p < 2.2e-16
NK cells
r = 0.99
p < 2.2e-16
Granulocytes
r = 0.98
p < 2.2e-16
M panel frequencies (%)
T panel frequencies (%)
NC
CAS
ASCVD

Figure S2

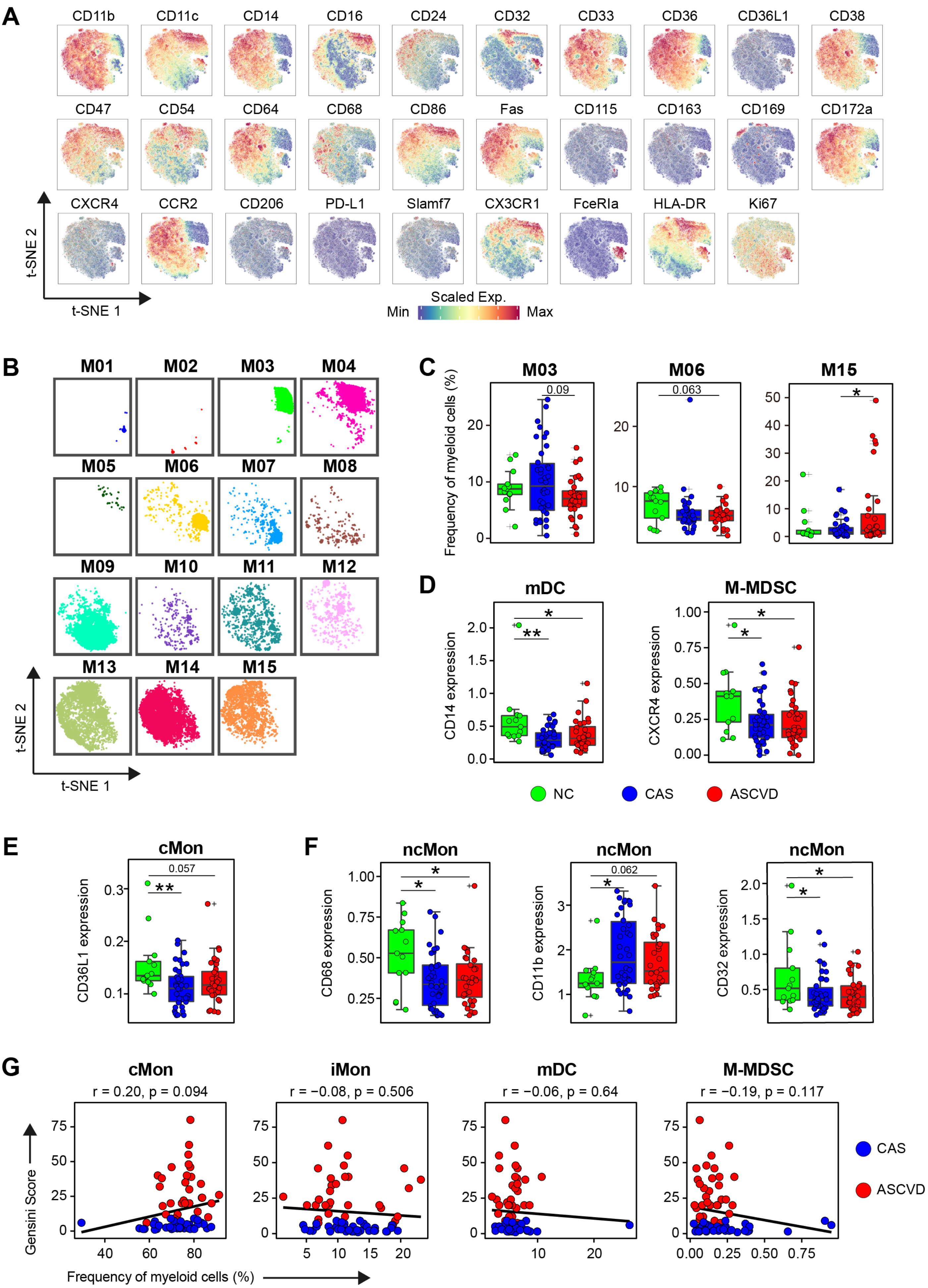
A
CD11b
CD11c
CD14
CD16
CD24
CD32
CD33
CD36
CD36L1
CD38
CD47
CD54
CD64
CD68
CD86
Fas
CD115
CD163
CD169
CD172a
CXCR4
CCR2
CD206
PD-L1
Slamf7
CX3CR1
FceRIa
HLA-DR
Ki67
t-SNE 2
t-SNE 1
Scaled Exp.
Min
Max
B
M01
M02
M03
M04
M05
M06
M07
M08
M09
M10
M11
M12
M13
M14
M15
t-SNE 2
t-SNE 1
C
Frequency of myeloid cells (%)
M03
0.09
M06
0.063
M15
*
D
mDC
CD14 expression
*
**
M-MDSC
CXCR4 expression
*
*
NC
CAS
ASCVD
E
cMon
CD36L1 expression
0.057
**
F
ncMon
CD68 expression
*
*
ncMon
CD11b expression
0.062
*
ncMon
CD32 expression
*
*
G
cMon
r = 0.20, p = 0.094
iMon
r = −0.08, p = 0.506
mDC
r = −0.06, p = 0.64
M-MDSC
r = −0.19, p = 0.117
Gensini Score
Frequency of myeloid cells (%)
CAS
ASCVD

Figure S3

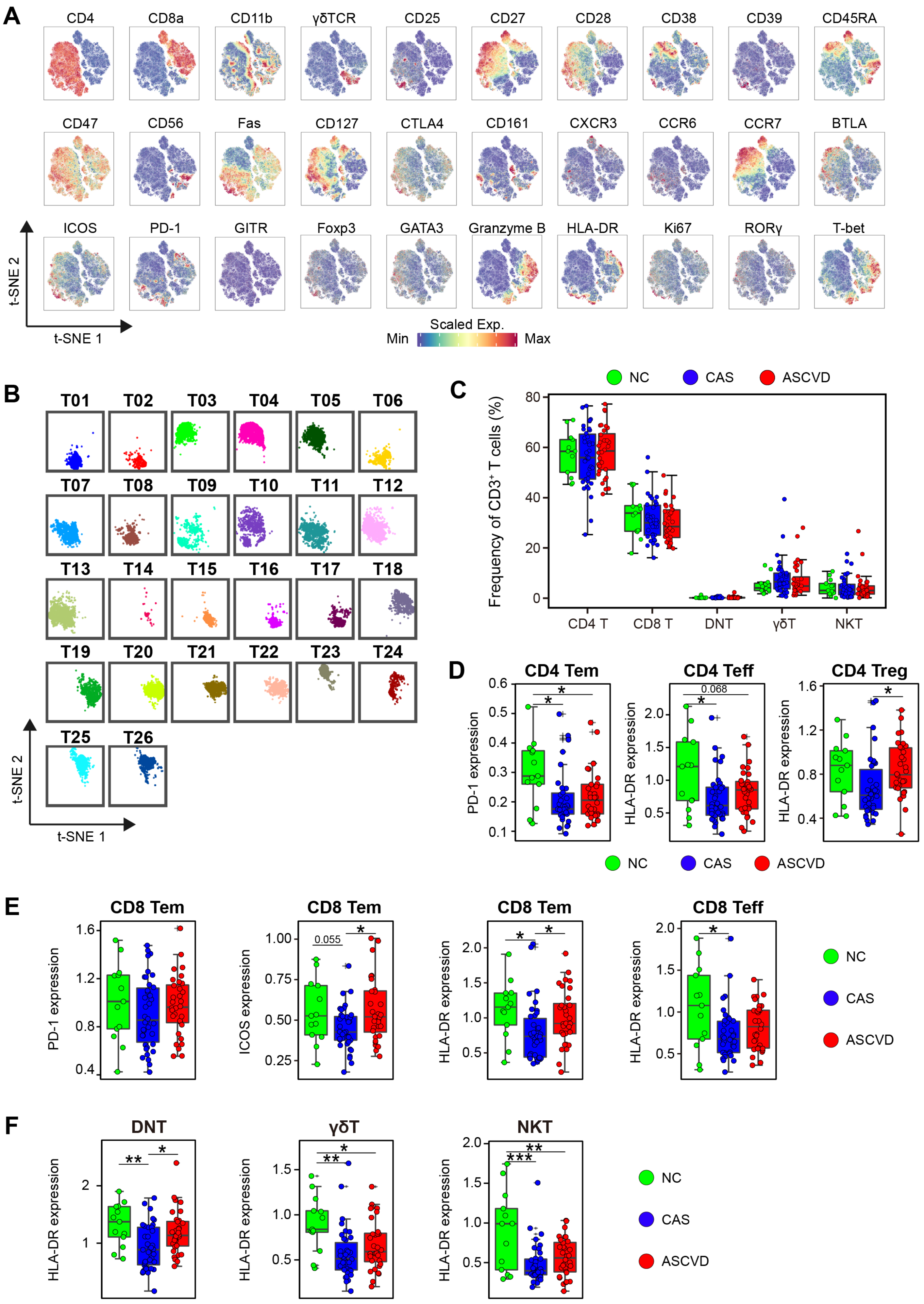
A
CD4
CD8a
CD11b
γδTCR
CD25
CD27
CD28
CD38
CD39
CD45RA
CD47
CD56
Fas
CD127
CTLA4
CD161
CXCR3
CCR6
CCR7
BTLA
ICOS
PD-1
GITR
Foxp3
GATA3
Granzyme B
HLA-DR
Ki67
RORγ
T-bet
t-SNE 2
t-SNE 1
Scaled Exp.
Min
Max
B
T01 T02 T03 T04 T05 T06
T07 T08 T09 T10 T11 T12
T13 T14 T15 T16 T17 T18
T19 T20 T21 T22 T23 T24
T25 T26
t-SNE 2
t-SNE 1
C
NC CAS ASCVD
Frequency of CD3+ T cells (%)
CD4 T
CD8 T
DNT
γδT
NKT
D
CD4 Tem
CD4 Teff
CD4 Treg
PD-1 expression
HLA-DR expression
0.068
E
CD8 Tem
CD8 Teff
PD-1 expression
ICOS expression
HLA-DR expression
0.055
F
DNT
γδT
NKT
HLA-DR expression

Figure S4

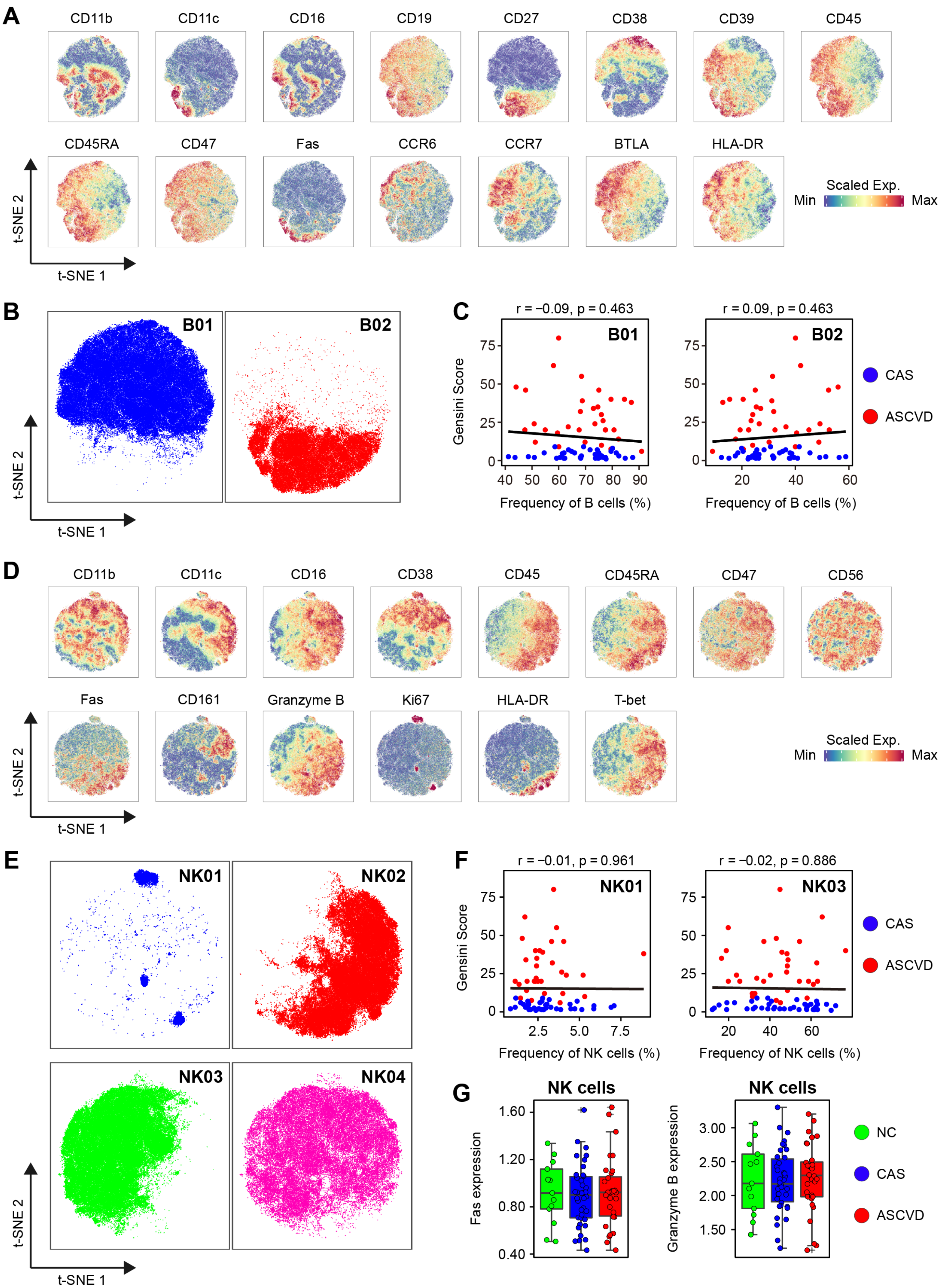
A
CD11b
CD11c
CD16
CD19
CD27
CD38
CD39
CD45
CD45RA
CD47
Fas
CCR6
CCR7
BTLA
HLA-DR
Scaled Exp.
Min
Max
t-SNE 1
t-SNE 2
B
B01
B02
C
r = −0.09, p = 0.463
r = 0.09, p = 0.463
Gensini Score
Frequency of B cells (%)
CAS
ASCVD
D
CD56
CD161
Granzyme B
Ki67
T-bet
E
NK01
NK02
NK03
NK04
F
r = −0.01, p = 0.961
r = −0.02, p = 0.886
Frequency of NK cells (%)
G
NK cells
Fas expression
Granzyme B expression
NC

Figure S5

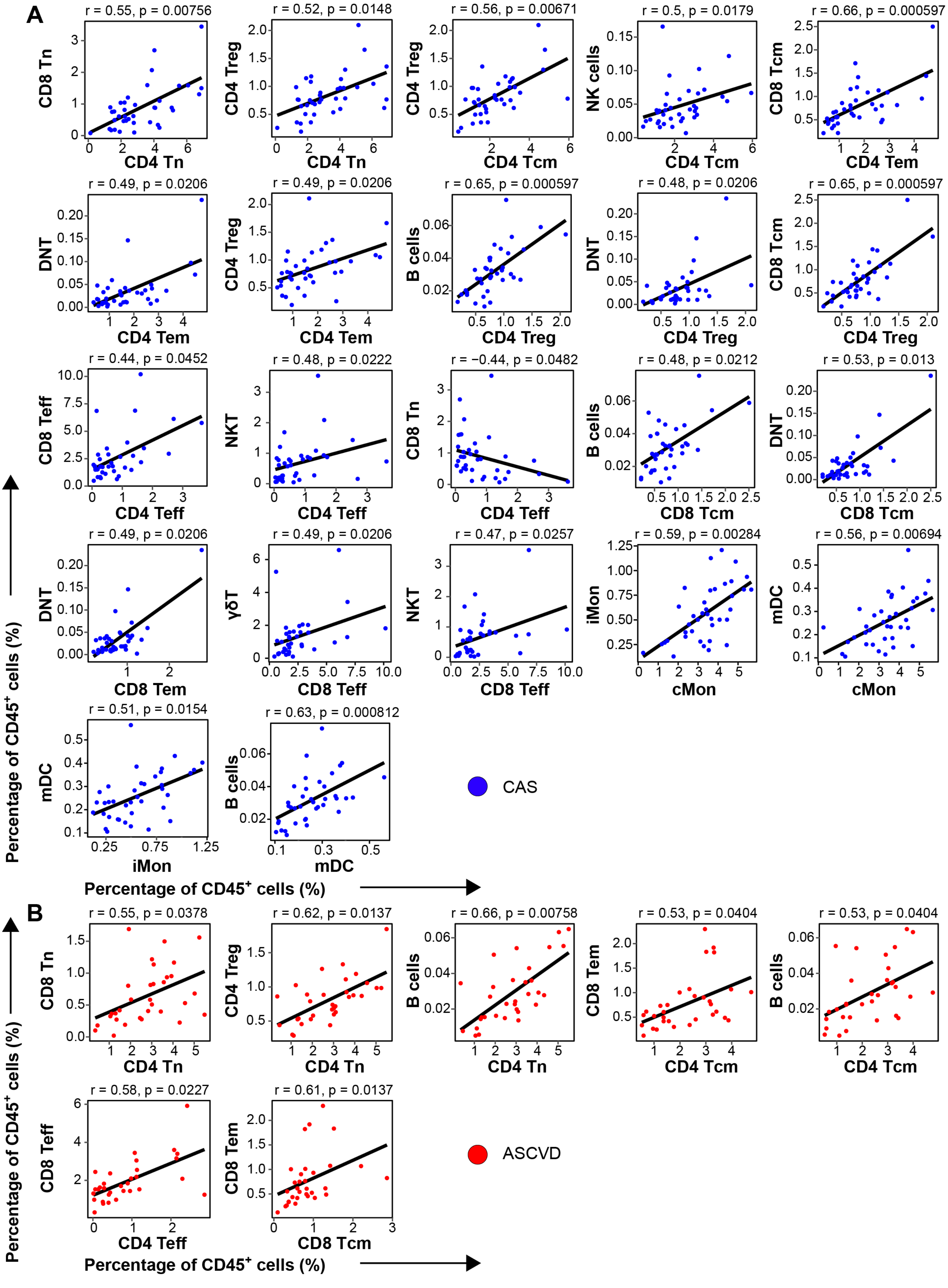
A
r = 0.55, p = 0.00756
CD8 Tn
CD4 Tn
r = 0.52, p = 0.0148
CD4 Treg
CD4 Tn
r = 0.56, p = 0.00671
CD4 Treg
CD4 Tcm
r = 0.5, p = 0.0179
NK cells
CD4 Tcm
r = 0.66, p = 0.000597
CD8 Tcm
CD4 Tem
r = 0.49, p = 0.0206
DNT
CD4 Tem
r = 0.49, p = 0.0206
CD4 Treg
CD4 Tem
r = 0.65, p = 0.000597
B cells
CD4 Treg
r = 0.48, p = 0.0206
DNT
CD4 Treg
r = 0.65, p = 0.000597
CD8 Tcm
CD4 Treg
r = 0.44, p = 0.0452
CD8 Teff
CD4 Teff
r = 0.48, p = 0.0222
NKT
CD4 Teff
r = −0.44, p = 0.0482
CD8 Tn
CD4 Teff
r = 0.48, p = 0.0212
B cells
CD8 Tcm
r = 0.53, p = 0.013
DNT
CD8 Tcm
r = 0.49, p = 0.0206
DNT
CD8 Tem
r = 0.49, p = 0.0206
γδT
CD8 Teff
r = 0.47, p = 0.0257
NKT
CD8 Teff
r = 0.59, p = 0.00284
iMon
cMon
r = 0.56, p = 0.00694
mDC
cMon
r = 0.51, p = 0.0154
mDC
iMon
r = 0.63, p = 0.000812
B cells
mDC
CAS
Percentage of CD45+ cells (%)
Percentage of CD45+ cells (%)
B
r = 0.55, p = 0.0378
CD8 Tn
CD4 Tn
r = 0.62, p = 0.0137
CD4 Treg
CD4 Tn
r = 0.66, p = 0.00758
B cells
CD4 Tn
r = 0.53, p = 0.0404
CD8 Tem
CD4 Tcm
r = 0.53, p = 0.0404
B cells
CD4 Tcm
r = 0.58, p = 0.0227
CD8 Teff
CD4 Teff
r = 0.61, p = 0.0137
CD8 Tem
CD8 Tcm
ASCVD
Percentage of CD45+ cells (%)
Percentage of CD45+ cells (%)

Figure S6

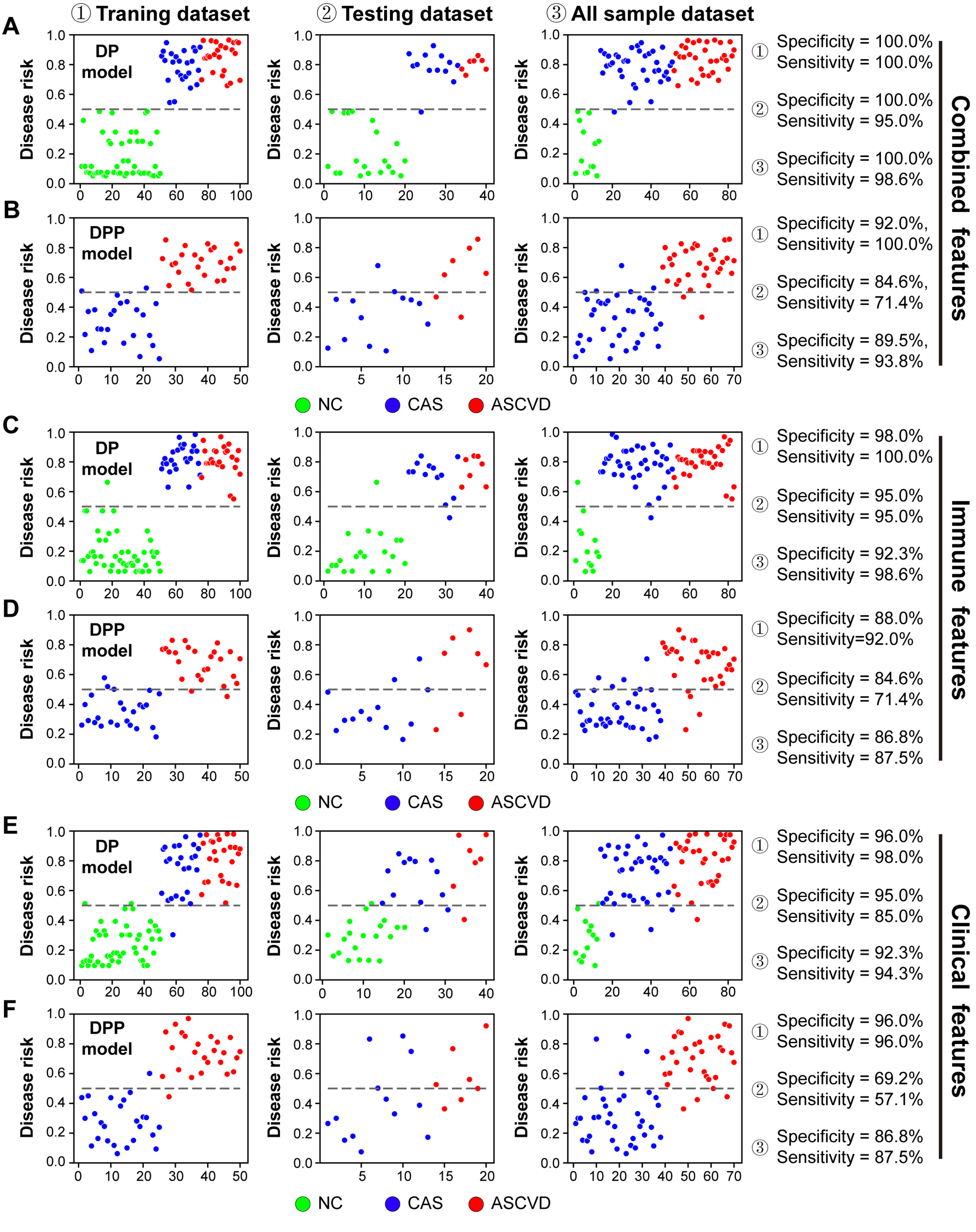

① Traning dataset
② Testing dataset
③ All sample dataset
A
B
C
D
E
F
DP model
DPP model
Disease risk
NC
CAS
ASCVD
① Specificity = 100.0%
Sensitivity = 100.0%
② Specificity = 100.0%
Sensitivity = 95.0%
③ Specificity = 100.0%
Sensitivity = 98.6%
① Specificity = 92.0%,
Sensitivity = 100.0%
② Specificity = 84.6%,
Sensitivity = 71.4%
③ Specificity = 89.5%,
Sensitivity = 93.8%
Combined features
① Specificity = 98.0%
Sensitivity = 100.0%
② Specificity = 95.0%
Sensitivity = 95.0%
③ Specificity = 92.3%
Sensitivity = 98.6%
① Specificity = 88.0%
Sensitivity=92.0%
② Specificity = 84.6%
Sensitivity = 71.4%
③ Specificity = 86.8%
Sensitivity = 87.5%
Immune features
① Specificity = 96.0%
Sensitivity = 98.0%
② Specificity = 95.0%
Sensitivity = 85.0%
③ Specificity = 92.3%
Sensitivity = 94.3%
① Specificity = 96.0%
Sensitivity = 96.0%
② Specificity = 69.2%
Sensitivity = 57.1%
③ Specificity = 86.8%
Sensitivity = 87.5%
Clinical features

Table S1. The date of patient recruitment in our CyTOF analyses.

Group-1	Sample ID	Date	Group-2	Sample ID	Date	Group-3	Sample ID	Date
**NC**	24_PB	2018.5.25	**CAS**	1_PB	2018.5.17	**ASCVD**	4_PB	2018.5.18
**NC**	56_PB	2018.6.5	**CAS**	3_PB	2018.5.17	**ASCVD**	6_PB	2018.5.18
**NC**	65_PB	2018.6.9	**CAS**	9_PB	2018.5.22	**ASCVD**	11_PB	2018.5.22
**NC**	73_PB	2018.6.15	**CAS**	10_PB	2018.5.22	**ASCVD**	12_PB	2018.5.22
**NC**	74_PB	2018.6.15	**CAS**	19_PB	2018.5.24	**ASCVD**	13_PB	2018.5.22
**NC**	75_PB	2018.6.15	**CAS**	20_PB	2018.5.24	**ASCVD**	14_PB	2018.5.22
**NC**	86_PB	2018.6.23	**CAS**	23_PB	2018.5.25	**ASCVD**	15_PB	2018.5.22
**NC**	107_PB	2018.7.21	**CAS**	28_PB	2018.5.25	**ASCVD**	21_PB	2018.5.24
**NC**	117_PB	2018.7.27	**CAS**	29_PB	2018.5.26	**ASCVD**	22_PB	2018.5.24
**NC**	119_PB	2018.7.31	**CAS**	30_PB	2018.5.26	**ASCVD**	25_PB	2018.5.25
**NC**	130_PB	2018.8.31	**CAS**	31_PB	2018.5.26	**ASCVD**	34_PB	2018.5.26
**NC**	131_PB	2018.8.31	**CAS**	32_PB	2018.5.26	**ASCVD**	36_PB	2018.5.29
**NC**	132_PB	2018.9.5	**CAS**	35_PB	2018.5.29	**ASCVD**	54_PB	2018.6.2
NA	NA	NA	**CAS**	37_PB	2018.5.29	**ASCVD**	59_PB	2018.6.6
NA	NA	NA	**CAS**	46_PB	2018.6.1	**ASCVD**	62_PB	2018.6.7
NA	NA	NA	**CAS**	47_PB	2018.6.1	**ASCVD**	69_PB	2018.6.13
NA	NA	NA	**CAS**	48_PB	2018.6.1	**ASCVD**	70_PB	2018.6.13
NA	NA	NA	**CAS**	49_PB	2018.6.1	**ASCVD**	71_PB	2018.6.13
NA	NA	NA	**CAS**	52_PB	2018.6.2	**ASCVD**	72_PB	2018.6.14
NA	NA	NA	**CAS**	57_PB	2018.6.5	**ASCVD**	81_PB	2018.6.20
NA	NA	NA	**CAS**	58_PB	2018.6.6	**ASCVD**	84_PB	2018.6.21
NA	NA	NA	**CAS**	63_PB	2018.6.7	**ASCVD**	88_PB	2018.6.23
NA	NA	NA	**CAS**	77_PB	2018.6.15	**ASCVD**	91_PB	2018.7.4
NA	NA	NA	**CAS**	78_PB	2018.6.15	**ASCVD**	99_PB	2018.7.17
NA	NA	NA	**CAS**	79_PB	2018.6.20	**ASCVD**	101_PB	2018.7.18
NA	NA	NA	**CAS**	83_PB	2018.6.21	**ASCVD**	111_PB	2018.7.25
NA	NA	NA	**CAS**	87_PB	2018.6.23	**ASCVD**	112_PB	2018.7.25
NA	NA	NA	**CAS**	90_PB	2018.6.26	**ASCVD**	114_PB	2018.7.27
NA	NA	NA	**CAS**	102_PB	2018.7.18	**ASCVD**	118_PB	2018.7.31
NA	NA	NA	**CAS**	103_PB	2018.7.19	**ASCVD**	127_PB	2018.8.16
NA	NA	NA	**CAS**	104_PB	2018.7.19	**ASCVD**	129_PB	2018.8.17
NA	NA	NA	**CAS**	115_PB	2018.7.27	**ASCVD**	133_PB	2018.9.5
NA	NA	NA	**CAS**	116_PB	2018.7.27	NA	NA	NA
NA	NA	NA	**CAS**	120_PB	2018.7.31	NA	NA	NA
NA	NA	NA	**CAS**	121_PB	2018.8.2	NA	NA	NA
NA	NA	NA	**CAS**	124_PB	2018.8.15	NA	NA	NA
NA	NA	NA	**CAS**	126_PB	2018.8.16	NA	NA	NA
NA	NA	NA	**CAS**	128_PB	2018.8.17	NA	NA	NA

Table S2. CyTOF antibody staining panel (T cell panel).

Mass Tag	Antigen	Clone	Supplier	Cat. no.
89Y	CD45	HI30	BioLegend	304002
115ln	CD3	UCHT1	BioXcell	BE0231
139La	CD47	CC2C6	BioLegend	323102
141Pr	CD56	NCAM16.2	BD Biosciences	559043
142Nd	TCRγδ	5A6.E9	In-house	N/A
142Nd	CD19	HIB19	BioLegend	302202
143Nd	CD196 (CCR6)	G034E3	BioLegend	353402
145Nd	CD95 (Fas)	DX2	BioLegend	305614
146Nd	CD123 (IL-3Rα)	6H6	BioLegend	306002
147Sm	CD66b	G10F5	BioLegend	305102
148Nd	CD33	WM53	BioLegend	303410
149Sm	CD25	24212	R&D Systems	MAB1020
150Nd	CD14	M5E2	BioLegend	301810
151Eu	CD38	HIT2	BioLegend	303502
152Sm	CD39	A1	BioLegend	328202
153Eu	CD274 (PD-L1)	29E.2A3	BioLegend	329710
154Sm	Ki67	SoIA15	eBioscience	14-5698-2
155Gd	CD45RA	HI100	BioLegend	304102
156Gd	CD11c	BU15	BioLegend	337202
157Gd	CD68	Y1/82A	BioLegend	333802
158Gd	CD197 (CCR7)	G043H7	BioLegend	353222
159Tb	CD357 (GITR)	621	BioLegend	311606
160Gd	CD28	CD28.2	BioLegend	302914
161Dy	CD152 (CTLA-4)	14D3	eBioscience	14-1529-82
162Dy	FoxP3	PCH101	eBioscience	14-4776-82
163Dy	CD183 (CXCR3)	G025H7	BioLegend	353718
164Dy	RORγ	600214	R&D Systems	MAB6109
165Ho	CD161	HP-3G10	BioLegend	339902
166Er	CD27	O323	BioLegend	302802
167Er	CD278 (ICOS)	C398.4A	BioLegend	313502
168Er	T-bet	4B10	BioLegend	644802
169Tm	CD15 (SSEA-1)	W6D3	BioLegend	323002
170Er	CD127 (IL-7Rα)	A019D5	BioLegend	351302
171Yb	GATA3	TWAJ	eBioscience	14-9966-82
172Yb	CD272 (BTLA)	MIH26	BioLegend	344504
173Yb	Granzyme B	GB11	Fluidigm	3173006B
174Yb	CD279 (PD-1)	EH12.2H7	BioLegend	329926
175Lu	CD16	3G8	BioLegend	302014
176Yb	HLA-DR	L243	BioLegend	307612
197Au	CD4	RPA-T4	BioLegend	300516
198Pt	CD8a	RPA-T8	BioLegend	301018
209Bi	CD11b	M1/70	BioLegend	101202

Table S3. CyTOF antibody staining panel (Myeloid cell panel).

Mass Tag	Antigen	Clone	Supplier	Cat. no.
89Y	CD45	HI30	BioLegend	304002
115ln	CD3	UCHT1	BioXcell	BE0231
139La	CD47	CC2C6	BioLegend	323102
141Pr	CD56	NCAM16.2	BD Biosciences	559043
142Nd	CD19	HIB19	BioLegend	302202
143Nd	CD184 (CXCR4)	12G5	BioLegend	306512
144Nd	CD38	HIT2	BioLegend	303502
145Nd	CD115 (CSF-1R)	9-4D2-1E4	BioLegend	347302
146Nd	CD54 (ICAM-1)	HA58	BioLegend	353104
147Sm	CD15 (SSEA-1)	W6D3	BioLegend	323002
148Nd	CD33	WM53	BioLegend	303410
149Sm	CD169 (Siglec-1)	7-239	BioLegend	346002
150Nd	CD14	M5E2	BD Biosciences	301810
151Eu	CD36L1 (SR-BI)	m1B9	BioLegend	363201
152Sm	FcεRIα	AER-37[CRA-1]	BioLegend	334602
153Eu	CD274 (PD-L1)	29E.2A3	BioLegend	329710
154Sm	CD163	GHI/61	BioLegend	333602
155Gd	CD206 (MMR)	15-2	BioLegend	321112
156Gd	CD24	ML5	BioLegend	311102
157Gd	CD172a (SIRPα)	SE5A5	BioLegend	323802
158Gd	CD204 (SR-AI)	351615	R&D Systems	MAB2708
159Tb	CD11c	BU15	BioLegend	337202
160Gd	CD319 (Slamf7)	162.1	BioLegend	331802
161Dy	CD66b	G10F5	BioLegend	305102
162Dy	CD32 (FcγRII)	Fun-2	BioLegend	303202
163Dy	CD68	Y1/82A	BioLegend	333802
164Dy	CD192 (CCR2)	K036C2	BioLegend	357202
165Ho	ProMBP-1	J175-7D4	BioLegend	346802
166Er	CX3CR1	K0124E1	BioLegend	355702
167Er	CD36	5-271	BioLegend	336202
168Er	CD95 (Fas)	DX2	BioLegend	305614
169Tm	CD40	82111	R&D Systems	MAB6321
170Er	CD86	Fun-1	BD Biosciences	555655
171Yb	CD64 (FcγRI)	10.1	BioLegend	305016
172Yb	CD117 (C-kit)	104D2	BioLegend	313202
173Yb	Siglec-8	7C9	BioLegend	347102
174Yb	CD279 (PD-1)	EH12.2H7	BioLegend	329926
175Lu	CD16 (FcγRIII)	3G8	BioLegend	302014
176Yb	HLA-DR	L243	BioLegend	307612
198Pt	Ki67	SolA15	eBioscience	14-5698-82
209Bi	CD11b	M1/70	BioLegend	101202

Table S4. Frequencies of CD45+ immune cell clusters identified by T cell panel.

Group	Sample	C01	C02	C03	C04	C05	C06	C07	C08	C09	C10	C11	C12	C13	C14	C15	C16
NC	107_PB_T	0.48725373	0	0.11197756	0.07061648	0.70818243	0.12408325	0.04943154	0.18864689	0.28145712	0.30869491	1.87436319	30.569875	0.08877501	2.94168087	0.10390711	0.53164123
NC	117_PB_T	3.46809753	0.0010067	0.20536775	0.18523366	4.25131375	0.59194234	0.04127489	1.1456299	1.39831276	0.43791652	2.25300501	8.88316186	0.02315421	0.86878612	0.07852296	0.34429299
NC	119_PB_T	1.58709352	0.00100832	0.45071843	0.54247542	4.8116965	1.2775397	0.04134106	0.7794303	1.47920343	2.91000756	2.87874968	3.26896899	0.03327451	2.91807411	0.55457525	0.92261155
NC	130_PB_T	0.28819324	0.00101835	0.02953217	0.11812868	0.528524	0.05091753	0.01731196	0.00712845	0.05295424	0.27902809	0.25356932	9.24153241	50.5774048	0.72506568	0.13442229	0.35336769
NC	131_PB_T	0.30882264	0	0.04440587	0.13422683	0.11908847	0.02523061	0.02220293	0.0040369	0.02623983	0.17762348	0.805361	24.2728539	30.3837071	1.51080879	0.01009224	0.61764528
NC	132_PB_T	0.90160879	0	0.01813291	0.23875004	0.70919842	1.05876072	0.06145043	2.19911955	0.9771626	3.24780642	11.5274965	7.0819104	0.13599686	8.79244865	0.5963714	0.41101272
NC	24_PB_T	1.5978078	0.00503723	0.03727547	0.08966261	4.16377026	17.49932	4.16477771	9.11636997	2.98908937	7.06420447	2.64051339	0.02417868	0	1.0316237	0.85834316	0.3646951
NC	56_PB_T	0.22416566	0.00201045	0.13872135	0.24728589	1.56312827	2.07076799	0.07941295	2.43566546	1.94611982	4.53558504	10.517692	6.5681544	0.04925613	7.91616405	0.36590269	0.16887817
NC	65_PB_T	3.27253273	0.02624486	0.05955565	0.11507364	1.87852665	2.24999243	0.06964983	2.35497189	3.49763292	7.19311173	13.3273441	3.9488427	0.04239555	1.43034512	0.22711902	0.28566526
NC	73_PB_T	1.13084159	0	0.02112782	0.04728608	0.37828865	6.63715479	0.05030434	10.1514161	4.53040897	6.07575834	4.29297248	1.1298355	0.04124956	1.69626239	1.57251371	0.60667036
NC	74_PB_T	3.31354994	0.00804748	0.08651041	0.07645106	4.4210844	8.50015089	0.03420179	4.43315562	8.08168192	3.12141636	4.59410522	4.86268987	0.05834423	2.81762398	1.20611608	0.35006539
NC	75_PB_T	0.39689318	0	1.97240839	0.12760869	6.04081469	8.28552194	0.05124444	3.05959426	6.34426213	6.8416346	4.76271817	6.44976538	0.12559911	5.36559388	1.29618279	0.3516775
NC	86_PB_T	0.83959837	0.0060548	0.06357536	0.16751602	0.22806398	0.53685857	0.02018265	0.26842929	1.30985418	3.07280892	2.98703265	42.4249458	0.09183107	1.46626974	0.33705031	0.06761189
CAS	102_PB_T	0.79813434	0.00301562	0.12163004	0.65338453	0.52170242	1.60531553	0.0281458	1.5862166	2.25668965	5.05920669	2.17124706	7.90193201	0.03317183	4.08315072	1.64853943	0.56090549
CAS	103_PB_T	0.26827161	0	0.01507144	0.23410968	0.71740048	0.54056227	0.0482286	0.75256717	0.41697647	3.20921166	2.81534473	18.0676406	0.11454293	15.1096196	1.17155316	0.38683359
CAS	104_PB_T	4.88876793	0.01310391	0.01108793	0.17539085	1.23075993	0.4415011	0.02620783	0.55137238	0.33162982	2.65505458	2.37584041	7.29384015	0.03729576	5.03291098	1.32248732	0.26107029
CAS	10_PB_T	3.21553255	0.00714563	0.0500194	0.15209979	0.47467385	2.55711398	0.02347849	8.35630142	2.77250362	9.44141606	7.53353341	0.16741185	0.00408322	2.82558543	1.4097303	0.53490129
CAS	115_PB_T	0.30253219	0	0.03025322	0.13210572	0.56472676	4.91614816	0.07664149	8.707885	1.93620604	4.30301625	1.82023537	2.39000434	0.00907597	2.46160362	1.91200347	0.8037272
CAS	116_PB_T	0.33559414	0.00100779	0.07759985	0.10984913	0.90096446	1.04608625	0.08767775	1.91077025	1.11058482	1.74448487	1.26880788	21.0819636	0.07356869	2.62630131	0.49583279	0.68832072
CAS	120_PB_T	0.25355684	0	0.02314209	0.34813756	1.00215322	1.66522448	0.04829654	0.39341557	0.7023122	5.58730606	0.68218864	11.9704989	0.11067957	4.1263357	2.12806633	1.49618659
CAS	121_PB_T	1.5451648	0.02723202	0.01613749	0.20171864	3.20127486	10.403639	0.09783354	6.08080849	3.300117	4.20885948	2.36515109	6.56695042	0.05849841	2.64856578	1.2627587	0.25618268
CAS	124_PB_T	0.49674202	0.00100555	0.3911592	0.19708793	1.04376156	0.63651355	0.03619982	0.45853109	1.03973936	3.12223474	5.78191618	9.88959054	0.19608237	3.64310997	0.23730995	0.56411391
CAS	126_PB_T	3.15132226	0.02027226	0.15305554	0.39733623	0.57269124	0.12974244	0.03344922	0.15102831	0.10845657	0.25948488	0.30002939	13.3533353	0.49464306	1.63394387	0.25137598	0.19461366
CAS	128_PB_T	2.08962435	0.01006563	0.30096227	0.22345694	0.36135604	0.14494504	0.0593872	0.38853324	0.30901478	0.49522889	1.47260136	18.3818497	0.22245038	3.13141684	1.04179249	0.51435359
CAS	19_PB_T	0.69392065	20.0472671	0.38718761	0.29164781	0.84175592	2.47900639	0.02715342	0.86287525	1.46628451	4.21280233	1.4612561	10.4289234	0.03017046	0.96344346	0.61648313	0.28360235
CAS	1_PB_T	1.64943452	0.00505341	0.04447005	1.0531316	0.93387102	4.10135128	0.05356619	1.1360076	1.52714189	7.90455111	8.03189715	0.83785614	0.01516024	44.0900316	1.61608199	0.29815146
CAS	20_PB_T	0.14543193	0.3510426	0.01604766	0.10531278	4.23758563	19.913142	0.03209532	1.87055555	10.0448332	4.57258056	0.56367411	0.66798391	0.00902681	1.09424992	1.31691123	0.18153917
CAS	23_PB_T	1.83003638	0.01310048	0.02015459	0.12193524	2.07592232	11.3278849	1.53174851	10.0631846	1.58918908	11.2261042	9.38296736	1.14377274	0.01612367	3.73262927	0.80819889	0.42425403

CAS	28_PB_T	0.1910527	0	0.32378405	0.07843216	1.48920552	21.7659303	2.04627498	12.9161681	3.19460226	9.26907259	4.01110117	0.9824131	0.01206649	3.78284347	1.54551579	1.16944363
CAS	29_PB_T	3.02783066	0.02014525	0.00906536	0.08158825	1.25303438	12.4819952	0.0241743	9.38869247	3.40656131	24.1803403	5.94687698	0.00302179	0.00100726	0.3747016	0.74335962	0.20245973
CAS	30_PB_T	2.63512623	0	0.02331073	0.07398624	0.74999747	5.4648464	0.01418914	2.47296462	1.65303496	15.3891372	4.59626826	0.06385114	0.01317563	0.15810757	0.81486211	0.20878308
CAS	31_PB_T	0.62615769	0.0130869	0.03422727	0.10368849	2.40899573	16.553918	0.02315374	14.1237819	9.45175968	7.08907143	3.6683579	0.02214706	0.00503342	0.33019248	0.56978336	0.23556415
CAS	32_PB_T	0.47581684	0.00301787	0.03219057	0.07242878	2.73116852	17.6011991	0.03722034	6.87067439	10.2909223	9.48314019	6.48639948	0.15391115	0.00502978	1.33792049	1.12465798	0.38427491
CAS	35_PB_T	1.78465019	0.00201998	0.1353385	0.3242064	0.37773581	0.6847724	0.02019978	0.61912313	0.73426185	6.44877842	4.21266324	0.51913424	0.03534961	0.20401774	0.26663704	0.57771359
CAS	37_PB_T	1.22589643	0	0.27084934	0.28095565	0.83680317	0.51744351	0.03132959	0.54169867	0.28803008	4.50034361	1.92121114	1.50483082	0.03739338	0.71552735	0.48712455	0.42951853
CAS	3_PB_T	0.38473934	0.00201434	0.00604303	0.16114737	0.48948513	0.94371928	0.0453227	1.11594553	0.34646684	3.48581903	2.33361534	2.76871324	0.03021513	51.3989606	2.68209653	0.24675691
CAS	46_PB_T	2.2518211	0.00503763	0.04836126	0.12392573	0.78284787	7.51916819	0.019143	23.0804107	1.7752612	5.92626923	7.39826504	0.06951931	0.0040301	1.15462505	0.46043948	0.17228698
CAS	47_PB_T	2.39896065	0.0050356	0.31220731	0.12186156	0.9899993	4.17350669	0.02316377	14.7261136	2.79274471	3.27716959	4.52499169	0.20948103	0.00604272	0.68484183	0.53377378	0.21451663
CAS	48_PB_T	0.89910291	0.00302389	0.10281222	0.10885999	0.28827739	7.18375164	0.00907167	5.69700635	2.52595504	2.76282633	1.48069751	0.41628868	0.00201593	0.15321036	0.24795888	0.10482814
CAS	49_PB_T	2.765353	0.00100741	0.40195841	0.10779336	1.86875403	6.8101225	0.01309639	17.8453417	2.60315925	0.87342843	2.44298034	0.30020954	0.00100741	0.38483237	0.33345422	0.06346712
CAS	52_PB_T	1.56865504	0.00904414	0.07536779	0.24921617	1.13855615	1.09032077	0.01808827	0.22409358	0.70242785	7.70861806	3.5282177	18.4168743	0.04421577	5.55209422	0.45622638	0.20198569
CAS	57_PB_T	0.9326404	0.00402868	0.02014342	0.18632665	0.57207316	2.18253968	0.08057368	2.7596487	0.99609218	5.59684957	3.64092337	3.1806462	0.03323664	5.55958424	2.84525824	0.14906132
CAS	58_PB_T	3.21358208	0.00904668	0.04623859	0.10252905	0.53073861	9.44775039	0.07639419	10.2780347	4.31426159	9.56334687	14.9129508	5.9537212	0.06332677	8.66571509	0.24023964	0.52470749
CAS	63_PB_T	0.40508676	0.00100768	0.04030714	0.05340696	0.74467442	1.22332171	0.0513916	0.78195852	1.28781314	5.75787501	5.93119571	17.784518	0.21060481	7.24319313	0.7174671	0.19750499
CAS	77_PB_T	0.8165158	0.00100804	0.03729764	0.07056309	0.51107841	6.16519828	0.03024133	3.11183242	7.54924296	5.71460253	4.49083688	5.32549747	0.11592508	1.1199371	0.93244088	0.8326445
CAS	78_PB_T	1.60163486	0.01107353	0.06946122	0.05939438	0.68152533	8.04139486	0.04429411	8.06555529	3.99351695	7.03672385	6.89276798	6.24949666	0.07248128	3.83446082	0.69964565	0.60803737
CAS	79_PB_T	3.72938929	0.00404379	0.02021897	0.22240869	0.64599614	1.84498115	0.06166786	4.05996947	1.78836803	4.27125772	7.69534054	2.89940051	0.02527371	3.4736193	1.03521134	0.41044512
CAS	83_PB_T	4.10493102	0.00510564	0.03573945	0.15725358	3.59640972	1.29989482	0.01531691	0.58816922	1.94728942	2.41700789	2.82137423	13.7076105	0.09598595	0.73316927	0.91697215	0.32880293
CAS	87_PB_T	1.45411276	0.0040336	0.23899079	0.070588	0.19562959	0.47697319	0.020168	0.37714159	0.60806518	2.42419353	1.87965755	39.0825577	0.1321004	1.16873557	0.28235199	0.1623524
CAS	90_PB_T	1.4841253	0.01411532	0.04839539	0.08166722	1.5002571	2.03663934	0.02722241	0.70879082	2.13343012	6.16436284	5.15410907	10.1116119	0.08065898	2.59520281	1.64745975	0.47689624
CAS	9_PB_T	1.96762293	0.01209604	0.17236861	0.10684838	1.96560692	2.34764026	0.01411205	9.08513598	10.0437474	2.22163982	2.86172207	0.05443219	0.00302401	0.283249	0.98280346	0.33465718
ASCVD	101_PB_T	3.24061463	0.00100891	0.03833853	0.23003118	0.69009353	1.18647659	0.0232049	1.45383738	1.02202448	3.37479948	1.72523381	4.38976159	0.01816036	0.72136969	0.70522716	0.3611893
ASCVD	111_PB_T	1.32935568	0.00100785	0.02519628	0.15016982	3.10821298	1.11367553	0.03023553	0.7800768	0.63293053	5.59558964	5.58147973	1.60147549	0.04232975	1.36563832	1.04917306	0.39104625
ASCVD	112_PB_T	1.56246854	0.00503373	0.12785664	0.23457163	0.76915333	0.63324273	0.03724957	1.19802678	0.47921071	3.9675828	2.78063022	0.57887849	0.01006745	0.83962549	0.68055975	0.37148898
ASCVD	114_PB_T	0.44443104	0	0.07340151	0.08546751	1.00248359	1.07186309	0.06736851	1.55751963	4.44833238	1.1814626	1.81291665	7.51309664	0.044242	0.73401506	0.20411652	0.53392055
ASCVD	118_PB_T	0.81592821	0	0.02615799	0.48090466	0.47788643	0.40243068	0.07646183	0.25252525	0.3370357	3.95689967	2.64497565	24.6518975	0.34106	5.96502877	0.40444283	1.76566461
ASCVD	11_PB_T	0.67925745	0.005039	0.04535102	0.22070828	1.50061476	3.4920283	0.010078	8.29419709	4.23477718	3.66637776	1.79791587	0.22070828	0.0060468	0.87678633	3.7590954	0.5421966
ASCVD	127_PB_T	1.82585549	0.00303635	0.00506058	0.34310699	0.76515895	0.38055525	0.03542403	0.45646387	0.22671376	1.6173598	1.33396759	2.89262472	0.14270822	1.76108013	0.3501918	0.27934374
ASCVD	129_PB_T	1.0374235	0.00601987	0.03912913	0.20768536	0.85080767	0.93508578	0.08427812	1.93639009	1.62937694	3.20156517	3.45038627	1.31433731	0.0371225	5.44898164	0.71034414	0.25283435

ASCVD	12_PB_T	11.5925106	0.03321925	0.16005637	0.25770083	0.26273404	2.76122408	0.01207973	11.1143547	7.80853634	3.10046306	3.83128649	0.22347493	0	0.62311254	1.00865714	0.64425206
ASCVD	133_PB_T	0.23398183	0	0.02823919	0.13514468	0.34693857	0.41753653	0.03630752	1.23647293	0.70497111	1.81840186	3.02461852	6.1531169	0.05849546	1.74982098	0.51233952	0.50830535
ASCVD	13_PB_T	1.90097929	0.00808497	0.1728163	0.26680411	0.37696187	1.85954381	0.01920181	5.21177576	3.15617136	7.6989156	6.95307684	1.26934077	0.0141487	3.04702422	1.82114018	0.52855511
ASCVD	14_PB_T	1.43099	0.00403096	0.04837149	0.10682038	0.5391406	6.05953725	0.01209287	8.58190906	10.6749839	8.87616898	6.39209126	0.11689777	0.00302322	1.2264189	1.76757498	0.78603676
ASCVD	15_PB_T	1.32949258	0.02417259	0.05237395	0.50762444	0.55798401	6.93551961	0.01812944	8.6124932	6.26976613	6.93451242	1.14215499	0.72316339	0.00302157	2.58848176	8.95191669	0.5469049
ASCVD	21_PB_T	0.59045037	3.32178541	0.03414169	0.12652508	1.03730481	12.4707536	0.02108751	1.07747151	1.19295075	7.61660893	0.32936687	2.30858061	0.01104584	0.47396696	2.27745142	0.16669177
ASCVD	22_PB_T	0.11466044	0.39225941	0.03922594	0.02313325	2.38373029	11.8140489	0.01408111	0.98366592	2.66535243	5.79638719	0.8187158	3.81598005	0.01005793	0.35303347	0.99372385	0.3037496
ASCVD	25_PB_T	1.76240275	0.01308466	0.26672572	0.15600938	1.71308365	21.8986845	1.59934778	5.30129941	3.33860075	13.2165108	11.5990458	0.50325607	0.01006512	3.17453927	0.15198333	0.38851368
ASCVD	34_PB_T	0.20602107	0.00100991	0.12926812	0.06665387	11.9411426	9.41738454	0.00908916	1.18361123	10.9726416	3.38116927	3.81239964	0.0424161	0.00302972	0.01514861	0.0696836	0.15552571
ASCVD	36_PB_T	1.04045306	0.0010131	0.13676842	0.1620959	0.69295997	0.74361494	0.01114409	1.97655688	1.16405118	3.33208384	7.9447253	0.98979809	0.04255017	1.3038589	0.21072467	1.43758801
ASCVD	4_PB_T	1.32354274	0.05137038	0.17627091	0.16821281	1.84731917	7.97550338	0.0241743	1.46556674	1.5521913	5.81391835	0.4442027	0.57514681	0.00302179	3.39245963	26.7176341	0.37067255
ASCVD	54_PB_T	2.16255326	0.01607846	0.02110298	0.20299059	0.83407026	1.13554144	0.04723048	1.60583648	1.90027333	7.55989227	7.24435244	1.00791864	0.01004904	6.29371332	1.00188922	0.3075006
ASCVD	59_PB_T	2.66362329	0.00402208	0.13976732	0.0693809	1.4459382	8.1306372	0.0492705	3.18448281	9.35133885	8.93203688	8.49865763	7.05774703	0.12066244	3.25989683	0.98339886	0.43036269
ASCVD	62_PB_T	1.0867489	0.0110585	0.05026591	0.11058499	2.49922088	0.83541937	0.06735631	0.78615878	0.4714942	4.03434167	4.26053825	20.897548	0.25535081	11.3872385	0.94902032	0.28048376
ASCVD	69_PB_T	1.82664973	0.00403234	0.06854977	0.17238251	1.06151334	7.80156858	0.02116978	5.54849896	2.10588923	18.536664	2.96880986	0.18347144	0.00806468	0.93953507	2.38109639	0.20363314
ASCVD	6_PB_T	1.5611492	0.08343553	0.04322564	0.05026237	1.40433061	7.78162006	0.0211102	1.20629687	1.21232835	3.70735238	0.25633808	0.0572991	0.00301574	0.8846177	45.8443073	0.36088381
ASCVD	70_PB_T	2.53805064	0.01005965	0.0784653	0.09355478	2.42940638	5.99857153	0.02414317	5.14551289	6.58001952	4.10534469	10.1491847	1.80973171	0.029173	1.57534178	0.60760309	0.28971803
ASCVD	71_PB_T	0.76394271	0.00201302	0.03724095	0.56666633	0.64014172	3.81166145	0.04931909	2.59982084	3.42717381	10.7143217	9.11195434	5.52877115	0.06039073	5.27412358	0.60390728	0.30396666
ASCVD	72_PB_T	1.95627273	0	0.22305729	0.09746197	2.85453047	22.09071	0.03014288	3.54781665	3.55786428	7.62413842	2.98514961	5.17553202	0.11152865	2.65558748	2.54606836	0.26023351
ASCVD	81_PB_T	4.94155746	0.00303598	0.02428781	0.25502201	1.6788949	0.80959369	0.01416789	0.51915195	1.16986288	1.86206548	6.90887011	4.73005111	0.04250367	0.80250974	0.3208015	0.20138643
ASCVD	84_PB_T	0.68152163	0.007026	0.0732711	0.37840008	0.85516411	2.20214795	0.01706313	0.70460705	2.72909766	4.77366255	2.16902539	24.8539597	0.08230453	2.59158888	0.93144635	0.47877145
ASCVD	88_PB_T	6.64416577	0.00909468	0.08690468	0.14955689	0.42643923	0.31528209	0.04850494	0.35469235	0.7457634	1.21262341	3.05682151	35.5581605	0.17684091	0.91350964	0.15056741	0.07174689
ASCVD	91_PB_T	2.94209576	0.0070578	0.18047812	0.05041288	0.53941783	1.38534598	0.01814864	2.01046571	1.31275143	2.35730634	11.0152146	21.9568264	0.33877456	2.82614614	0.27626259	0.57672336
ASCVD	99_PB_T	2.26224335	0.00100499	0.06130468	0.14974423	5.09833874	2.62906646	0.14170427	3.56371165	1.6703014	2.86926022	5.03401907	2.03712451	0.00100499	0.96580003	0.81304081	0.99795986
**Group**	**Sample**	**C17**	**C18**	**C19**	**C20**	**C21**	**C22**	**C23**	**C24**	**C25**	**C26**	**C27**	**C28**	**C29**	**C30**	**C31**	**C32**
NC	107_PB_T	0.46304236	2.84887064	5.40518728	5.28413046	0.54979975	9.08834122	1.53036004	0.52457958	0.09180143	1.17122479	0.43378696	0.34702957	0.35913525	5.61401031	0.04640512	1.10565234
NC	117_PB_T	0.58288199	3.72682063	10.4445608	1.24831377	0.99059738	34.6064792	0.76308213	0.30100469	0.17818672	3.86876598	1.50301005	0.41476232	0.31912537	5.38083637	0.09161012	0.96643647
NC	119_PB_T	0.29947063	4.24602975	4.27829594	4.06453239	2.04184522	27.1762037	5.79984875	0.88026216	0.13208974	1.86337283	1.04663474	0.25107134	0.72195614	4.43559365	0.34686161	0.33778674
NC	130_PB_T	0.27800974	1.14462616	0.18330312	4.84734923	0.1547893	2.40025255	3.27603414	0.63137742	0.09266991	3.2974195	0.42465223	0.48473492	0.23320231	3.85751237	0.04073403	0.72404733
NC	131_PB_T	0.14028218	0.5853501	0.66911572	6.02405991	0.03633208	0.64489433	1.37052661	0.05954423	0.08679329	3.48787922	0.60351614	0.67315261	0.61360838	5.1975052	0.11000545	0.79930565
NC	132_PB_T	1.3136289	1.4405593	15.5036417	4.7850746	2.69374515	1.20785357	3.00603423	0.4885813	0.14304855	3.11886125	0.94190416	0.55003173	0.72733134	4.25619793	0.11081225	0.48757392

NC	24_PB_T	0.01611912	0.35663554	0.62360847	0.55207987	0.99233334	1.06688427	0.27704738	0.28006972	0.09268494	0.21660068	5.79482375	1.93832422	0.37678444	2.60323793	0.06951371	0.71629341
NC	56_PB_T	0.67551267	6.06554081	6.58725372	17.358263	1.28568556	2.62866908	2.93727382	1.22235625	0.02814636	0.53277041	0.77603538	0.12967431	0.5991154	3.72135103	0.11660635	0.8283072
NC	65_PB_T	0.98014475	1.72509514	5.39130084	12.1745889	3.52387778	2.12583403	2.55281779	0.768167	0.07974401	0.40982365	1.13761394	0.26850515	0.48552999	6.13019472	0.11911131	1.34050693
NC	73_PB_T	0.84813119	4.09577947	4.71351678	6.33231048	9.06283012	0.45173298	0.98294683	0.92358771	0.14688868	0.90950249	4.61793853	1.3823633	0.82599728	3.08667438	0.13481563	0.66099904
NC	74_PB_T	1.15078966	2.49270697	4.82949401	1.50186098	4.16155316	11.2956443	1.04013681	1.037119	0.17905643	1.9132884	4.74901921	1.45759984	0.46474198	3.2059149	0.11065285	1.037119
NC	75_PB_T	0.53354501	3.60821117	3.17815982	2.66370588	2.09499312	10.2097003	3.64739809	1.5343187	0.16076686	0.7314892	2.06384454	0.53254022	0.35268229	4.87525497	0.14167579	1.30221155
NC	86_PB_T	0.40768959	1.97386346	1.70341591	1.24426056	0.80125132	2.59347091	4.63898279	3.05262627	0.20687219	3.52994601	1.41278571	2.03340229	0.31182199	4.4290832	0.10898633	0.90317372
CAS	102_PB_T	0.20405702	11.9589473	5.16877425	8.34623349	3.27898514	1.24746185	11.6191874	3.94945819	0.03015621	0.20606743	0.20405702	0.19802577	0.43525462	3.34231318	0.09448946	0.21812991
CAS	103_PB_T	0.3275526	5.08510339	8.79368205	9.94112091	1.55838675	1.16753411	10.9810502	1.09619597	0.08339529	2.17832526	0.77266242	1.02083878	0.43506219	1.56943914	0.0401905	0.69830999
CAS	104_PB_T	1.16624835	3.75578336	8.7503906	17.8747467	3.7658633	1.51602205	7.75146915	1.32349532	0.06854355	2.63287873	0.9001381	1.09568881	0.59572409	3.69227978	0.09575937	0.41327729
CAS	10_PB_T	0.09187236	0.5134644	18.1948102	5.47763418	2.94808191	0.80031032	1.12084278	0.23478492	0.10820522	0.84012168	1.34031563	0.70027153	0.44200813	4.54972336	0.04797779	0.72477083
CAS	115_PB_T	0.33278541	11.5920253	11.9379204	1.76779646	9.51766284	3.51744098	4.66000424	2.18226556	0.19059528	3.40147031	2.16814739	1.1859262	0.33883606	2.49286528	0.06050644	0.89650374
CAS	116_PB_T	0.48071593	19.0008768	6.15759823	4.36675502	3.89007024	3.76409647	4.83336189	2.70188558	0.03930382	0.88080865	0.9584085	0.54118335	0.38598365	3.20779627	0.06046741	0.30838381
CAS	120_PB_T	0.21230354	5.00875375	1.15911698	3.96434105	2.79717465	8.27178878	24.3575554	4.54490572	0.04024712	0.4648542	0.90455396	0.15595758	0.23745799	1.45090858	0.29481013	0.31996458
CAS	121_PB_T	1.4271594	6.27849276	0.5799411	0.49521927	2.44482995	9.58970428	1.7085569	4.82914431	0.06051559	0.70500666	2.14326058	0.53758018	0.18053819	4.0273127	0.09884213	1.04288538
CAS	124_PB_T	0.44143673	3.55361596	7.81312847	8.47075859	1.4952538	8.76538492	8.71209074	1.22878288	0.19708793	4.26956802	1.01962835	1.14934438	0.31272625	5.47019548	0.0754163	1.09906685
CAS	126_PB_T	0.15710999	3.73719047	0.75615516	33.255623	0.60208602	0.93049657	5.80394701	1.26296157	0.25441682	5.09745887	0.79568606	0.41862209	0.53113312	3.72908157	0.23414456	1.85997953
CAS	128_PB_T	3.94069332	3.77964327	3.84909611	8.13705359	2.54157104	2.06043403	4.38458751	2.93614366	0.13487941	3.10524621	2.90695334	0.98139872	0.61903612	4.97040705	0.07951846	1.27732818
CAS	19_PB_T	0.44451149	2.05561422	0.5450797	2.22155177	1.35767084	2.11997787	2.97883039	16.2176296	0.23331825	1.05797757	1.66641525	1.01875597	0.64866496	5.83094484	0.07442048	0.4606024
CAS	1_PB_T	0.03032049	0.27389507	0.70646736	0.62662341	0.19000839	1.74747077	1.84449633	0.41134795	0.08995078	0.26479892	0.89445438	0.28096985	0.51342692	3.92144972	0.17484814	0.60640975
CAS	20_PB_T	0.04112213	7.79214266	0.44833154	0.75624605	0.8344784	1.80435895	1.21059547	28.2689588	0.03109234	0.27882812	0.96185671	0.33399196	0.24372386	2.54355436	0.14142002	0.78031754
CAS	23_PB_T	0.18441446	0.60766076	2.16661796	6.69736882	1.57205768	1.24353794	1.46322292	0.61572259	0.16224441	1.14780365	3.59154717	0.7880443	0.34363569	3.54922254	0.09170336	0.95129644
CAS	28_PB_T	0.20915243	1.19960985	2.76322537	4.04830617	2.62043862	0.70790053	1.73857957	0.80946013	0.11563716	0.69281742	3.39470482	0.66365675	0.23529648	1.18754336	0.0693823	0.61740188
CAS	29_PB_T	0.0161162	0.5459362	1.9027186	8.97873669	3.94242488	0.29915692	0.43010103	0.14202399	0.02719608	0.09166087	1.01834225	1.26814331	0.52377643	4.83083029	0.1470603	0.36160719
CAS	30_PB_T	0.39628244	0.36689065	0.85742954	7.82936544	4.14728329	0.53918737	1.04695592	0.46824166	0.23614785	3.14289479	6.92632795	1.84458836	0.79763244	2.17904669	0.15506704	1.21215807
CAS	31_PB_T	0.03422727	1.36909076	7.30752195	3.10461464	10.8228638	1.31472981	0.57683015	0.51844246	0.08858823	0.29797858	1.34795039	0.26274462	0.25066441	4.66799549	0.16006282	1.02379802
CAS	32_PB_T	0.37522131	2.32476259	3.19491389	3.33876549	9.67427169	2.68489458	1.14779495	1.1870272	0.10361339	0.4315548	2.14872042	0.71925801	0.21728634	3.64256398	0.11166103	0.63375181
CAS	35_PB_T	0.4959045	1.51094323	2.20177556	40.3924816	4.91460545	1.08775793	1.87958914	0.90393997	0.10907879	1.03927846	0.70396219	0.57973356	0.72820192	4.01470544	0.089889	0.55549383
CAS	37_PB_T	0.15159478	0.88329223	2.02732749	36.4322675	1.07430165	2.30727251	2.45381412	0.66196386	0.17079678	4.04758055	1.48866071	0.80143105	0.57403889	3.64029591	0.10914824	0.43760359
CAS	3_PB_T	0.11179599	0.85810974	3.69027476	1.84614455	0.28502941	0.47538474	2.84626541	0.68789783	0.12287487	1.39795343	1.48960599	1.40097494	0.13294658	1.38788172	0.07150915	0.40790428
CAS	46_PB_T	0.08765478	0.82516397	9.26722618	6.05221001	4.18526392	0.33349118	0.29419766	0.16019667	0.06750426	0.20553535	1.03875953	1.34907761	0.48865022	1.96568366	0.26598692	1.59894411

CAS	47_PB_T	0.1883315	1.69498353	13.7572639	5.65800207	6.95416595	0.34947076	0.39479117	0.16416062	0.13898261	0.29307202	1.79166709	0.63448581	0.69189167	2.95690532	0.12286868	0.7180768
CAS	48_PB_T	0.65517589	1.25188993	3.94214293	5.26862211	26.3773813	0.21771999	0.54833182	0.87390384	0.15119444	0.27819776	1.53411954	0.16933777	0.32254813	4.40882976	0.21872795	1.42828344
CAS	49_PB_T	0.10577853	0.51075919	14.3073017	0.51579626	3.84530142	1.46175854	0.17831238	0.12391199	0.32438749	3.62870729	10.2101467	2.3855577	0.76261283	4.40139426	0.18838652	0.46844778
CAS	52_PB_T	0.24620146	1.37068896	2.47608329	4.24571911	0.60193745	2.32132808	19.2419005	2.97552054	0.04321087	1.96358228	1.44806656	0.25725541	0.49039312	4.11809631	0.1537503	0.54566283
CAS	57_PB_T	0.83494481	3.48783337	7.94657965	10.0837966	4.69744581	1.0998308	4.55744904	1.2700427	0.25985013	3.16654581	5.0076545	0.80875836	0.22157763	3.24309081	0.11280316	1.4442833
CAS	58_PB_T	0.30256122	0.99010896	3.51513811	0.94789112	0.92276145	0.43424068	1.82039323	0.64030397	0.03518154	0.63728841	1.959109	1.53693056	0.42418881	2.86679265	0.20907885	0.84033613
CAS	63_PB_T	1.19510671	4.55067615	3.26185534	12.8610008	1.16789939	0.93512566	7.02452689	1.97706524	0.08363732	1.14371511	1.01070155	0.56228461	0.54313872	2.72476269	0.18642052	0.8625728
CAS	77_PB_T	1.67234532	8.35265418	1.99794359	7.61274974	4.17632709	1.11792101	2.14310195	2.14612609	0.3739844	1.16832322	2.79127437	1.07457511	0.4768049	3.46464789	0.14112619	0.96772242
CAS	78_PB_T	0.6704518	2.81670291	6.84545381	5.27603286	3.28581783	1.02681807	2.69086736	0.7479665	0.11878876	1.02279133	2.67375372	0.56474994	0.52146251	2.47040348	0.14294918	0.39059354
CAS	79_PB_T	1.40521852	5.07293994	10.6190038	11.8493282	5.1346078	1.89148478	2.35753207	0.89671138	0.20218971	1.02004711	2.27463429	0.43066409	0.83605447	2.33630215	0.14254375	1.17371129
CAS	83_PB_T	1.86968376	2.50993046	2.7825714	4.3826776	3.17264196	12.4199692	2.55894456	3.11954335	0.20218317	3.75979006	5.09848771	1.15285252	0.81179606	5.26799481	0.06943664	1.40609204
CAS	87_PB_T	0.39730959	1.91495155	2.31730314	6.32065102	1.70117075	0.55159478	6.81174181	1.75360755	0.110924	1.94016155	1.11226517	0.21882279	0.21277239	5.56939304	0.0615124	1.83327115
CAS	90_PB_T	2.2029985	2.18585846	1.80474476	1.39237571	2.39557182	6.42650454	4.97968402	6.84996421	0.13812851	4.01883387	0.79348275	1.12620106	1.12216811	3.8141617	0.08570017	0.68358489
CAS	9_PB_T	0.01310405	0.70761849	23.8937161	2.54117694	2.31236014	2.00391105	0.12600044	0.14414451	0.12902445	0.70358648	2.15410358	1.06949176	0.59371409	4.90091325	0.13708848	0.43243352
ASCVD	101_PB_T	0.49032961	4.04370592	5.79517136	13.3075053	8.02788624	2.22060797	6.05849652	1.84428504	0.10089087	1.67882402	1.14208461	0.56397994	0.54077504	3.73195315	0.05548998	0.97157904
ASCVD	111_PB_T	1.97639613	1.18220941	4.58370708	19.400127	7.38250975	2.83407746	4.93343143	0.9987805	0.09473801	0.57044376	0.5613731	0.55331029	0.92722307	7.26963042	0.20257808	0.56439665
ASCVD	112_PB_T	1.047015	3.5487768	4.52229941	30.7329105	8.88251284	1.33494413	3.88100272	0.83358502	0.20034229	6.24081345	2.75848183	1.64502165	0.51344005	4.90184234	0.05033726	0.99969798
ASCVD	114_PB_T	0.38510653	35.606769	4.57301439	4.49056338	2.44738721	5.63683348	1.88330166	1.92452716	0.12166551	1.1714076	0.55503605	0.48062904	0.17797352	5.66498748	0.08546751	0.76518557
ASCVD	118_PB_T	0.6177311	3.55949938	1.53527305	10.7308141	1.44875045	2.92667713	14.8154855	1.6067045	0.02414584	0.41349753	0.66401062	0.25856171	0.41047929	4.08567749	0.1941728	0.4708439
ASCVD	11_PB_T	0.03124181	0.84957572	19.3931026	2.700905	3.90724205	1.86745409	0.59863342	0.41118255	0.12194385	1.97327313	5.46127023	1.38068651	0.59359442	3.20278959	0.13302965	1.10858041
ASCVD	127_PB_T	0.37448256	1.74387417	2.61732943	40.4582857	2.58392964	0.3572766	4.88345496	0.82993431	0.08805401	2.58190541	0.7074684	0.3704341	0.81880105	2.2711861	0.37954313	0.94430331
ASCVD	129_PB_T	0.36018862	9.50837765	5.3075148	39.6368014	6.97301094	1.32437042	3.73231664	2.29858533	0.04715561	1.25012541	0.34714558	0.10334103	0.19865556	1.49894652	0.04013244	0.25082773
ASCVD	12_PB_T	0.0573787	1.70626132	26.396215	2.56492853	6.09925508	0.33118583	0.34829877	0.32313268	0.08153815	0.67243809	1.09422186	0.16206966	0.24964767	5.57278035	0.03825247	1.25327159
ASCVD	133_PB_T	1.97371739	6.67150767	11.6849717	9.62754531	8.1510393	1.50171956	4.70081591	1.38674574	0.51233952	7.1485482	2.80173066	3.67714542	0.52242494	6.09865561	0.06353817	1.3121136
ASCVD	13_PB_T	0.10611527	0.72259447	24.8036867	5.58065266	2.24863313	0.99546231	2.14453911	0.39616368	0.04244611	0.44669476	1.01062163	0.36079192	0.44871601	5.04098071	0.09600906	0.46993906
ASCVD	14_PB_T	0.04030958	1.34230893	18.4718639	2.34097872	3.38600451	0.57945018	0.89084166	0.37891003	0.11790551	1.27882135	1.31610771	0.83440826	0.19550145	5.88519832	0.05844889	1.07223476
ASCVD	15_PB_T	0.02014383	1.89855569	17.7205245	1.30129122	3.27941502	0.43208509	1.92272828	1.35668674	0.06345305	1.22373749	3.55135669	0.78359487	0.52776827	5.06415809	0.09165441	1.06460125
ASCVD	21_PB_T	0.26510017	1.2773008	0.4267711	1.78239695	2.4561932	1.0714465	3.99056083	32.2498368	0.14158759	3.23542702	5.65948687	0.70894211	0.22392931	3.82487322	0.06727921	1.11261736
ASCVD	22_PB_T	0.40231735	1.29345027	0.4697055	0.91929514	2.10411973	3.05358867	2.54968619	29.1157065	0.0905214	1.09128581	1.81545703	0.74730447	0.55218056	3.71841809	0.1056083	0.74026392
ASCVD	25_PB_T	0.03522792	0.22747174	1.47152074	3.22989744	0.43481324	0.17312009	1.19573641	0.13789216	0.15701589	0.59786821	1.84795628	1.49064447	0.4066309	3.70396465	0.10467726	1.30142019
ASCVD	34_PB_T	0.16360496	1.21693816	0.95032267	2.45306456	2.74896737	16.5230915	0.30297216	0.60089478	0.09089165	0.30802169	1.11291772	0.79883659	1.0816106	11.1433159	0.14845636	0.67562791

ASCVD	36_PB_T	0.22389496	1.1356844	10.4511332	25.6709251	2.67660855	0.8033878	1.16607738	0.1681745	0.17627929	1.88132554	2.90252971	1.36768416	0.29987741	3.34728033	0.13879461	0.60684653
ASCVD	4_PB_T	0.05237764	0.14504578	1.16238077	0.05036312	1.10194502	5.01918835	1.17446791	3.04394686	0.31628038	5.78873679	4.44907785	2.66521621	0.86826016	3.82457519	0.10072624	1.12611932
ASCVD	54_PB_T	0.08642174	6.18216898	8.78085055	29.7481711	2.10426883	1.30838492	4.40348903	1.02701182	0.04321087	0.31654474	0.41301552	0.25323579	0.43311359	4.56628346	0.09144626	0.34669186
ASCVD	59_PB_T	2.12265337	4.24731777	1.50325286	2.98438427	3.05376517	2.76115876	2.35291752	2.6143528	0.18099366	2.50374556	4.5208193	0.48566631	0.46756694	2.60228655	0.13775628	0.50577671
ASCVD	62_PB_T	1.06161595	1.61454092	2.77065677	8.86187934	0.81129173	4.91399503	5.89820149	1.65877492	0.04121804	1.03849363	1.14304672	0.42726021	0.29757417	2.80785355	0.22820722	0.45239316
ASCVD	69_PB_T	0.20867356	1.93451481	1.79439102	9.76027742	5.73801891	0.42037138	2.68957035	1.12804694	0.1996008	0.98993931	5.88217504	0.5917458	0.26411823	3.71277647	0.20060888	0.75203129
ASCVD	6_PB_T	0.01407346	0.37294678	0.95699552	0.17692354	0.60214319	0.92281711	0.49357647	1.19121816	0.16687107	3.06902029	3.39974668	1.24851726	0.7529303	3.55354953	0.06735158	1.06958322
ASCVD	70_PB_T	2.15377187	2.50183589	5.81146197	5.44126671	6.74097398	3.3760198	0.86211233	0.89128532	0.12675164	0.95164324	2.36100073	0.32190892	0.37723702	2.26442806	0.10361443	0.92548814
ASCVD	71_PB_T	0.5062756	3.3426268	2.03114149	13.3805723	2.42066168	0.57975099	4.72658098	1.48863145	0.06542329	0.65020684	2.49615009	0.60491379	0.28282991	4.1146216	0.19526335	0.79816412
ASCVD	72_PB_T	0.83696722	8.11647208	0.73850049	6.72688544	1.31221992	1.5141772	3.44633563	1.59053916	0.09243816	2.20746338	2.46970641	0.31750497	0.22506682	4.17378373	0.11655246	0.62596708
ASCVD	81_PB_T	13.2682285	1.40363305	2.37818145	5.57000455	3.22218287	10.1178971	0.97758437	1.19921065	0.42200071	0.98264434	2.29722208	1.05247179	0.61326722	6.7054597	0.12245104	1.67383494
ASCVD	84_PB_T	0.56107598	5.16812205	3.32731105	4.0981632	3.2359731	6.74997491	10.48379	5.90484794	0.10739737	1.2636756	1.39917695	0.34427381	0.38843722	5.19522232	0.04617083	0.78791529
ASCVD	88_PB_T	0.89430976	2.52831981	2.67484514	8.40145919	1.16816055	2.25547954	2.6728241	1.24496003	0.12631494	3.76216413	0.86096262	0.4456391	0.67300599	3.85311088	0.04244182	0.72959508
ASCVD	91_PB_T	1.63640213	0.81265565	3.65493391	3.32321715	1.4135772	0.92053922	1.35409	0.68359867	0.16535425	2.61844507	2.44300824	1.37123038	0.57974814	7.60427905	0.0927597	1.09093476
ASCVD	99_PB_T	1.18991387	1.44216757	8.737425	5.07723385	8.38567681	11.6941198	1.98285479	0.94469513	0.20099896	2.55067686	1.58085686	0.68942645	0.65425163	4.95261449	0.05225973	1.30950826
**Group**	**Sample**	**C33**	**C34**	**C35**	**C36**	**C37**	**C38**	**C39**	**C40**	**C41**	**C42**	**C43**	**C44**	**C45**	**C46**		
NC	107_PB_T	0.36922332	1.84813421	1.18534809	1.40829441	7.02331353	1.35281003	1.7583504	3.6347312	0.69305033	1.92883876	0.04943154	3.06576412	1.33666912	1.04209751		
NC	117_PB_T	0.14899229	1.104355	0.50234562	0.85771236	0.97952363	0.44597016	0.99865102	1.3640848	0.3895947	1.2976423	0.13288501	0.50234562	1.37717197	0.33523265		
NC	119_PB_T	0.63524074	1.08394253	1.02646836	0.44466852	3.8699269	0.98916057	2.07713638	2.12150239	2.17494328	1.65969246	0.17544744	0.32064532	0.91958659	0.12301487		
NC	130_PB_T	0.19857838	1.82895782	1.18128679	1.02955254	3.08865761	0.69553351	0.47251472	1.81877431	1.33811279	0.89920365	0.73015744	0.50612029	1.33302104	0.1507159		
NC	131_PB_T	0.22606625	1.51686414	1.04959328	0.91435723	2.46553499	1.07886079	0.55911027	2.98326706	1.06170397	3.11244777	0.06761803	1.07684234	3.79771108	0.52580587		
NC	132_PB_T	0.42713087	1.41839685	1.32269536	1.38918271	2.99293824	1.14942529	1.13431453	2.59905104	2.06715223	1.73673023	0.13095994	0.51880282	4.2330281	0.14808547		
NC	24_PB_T	0.36066532	3.13113912	1.40740069	4.27257432	3.57945215	1.06688427	6.98864609	0.47652149	1.33687954	2.36145112	0.62058613	3.18755604	3.07774453	0.51278951		
NC	56_PB_T	0.37293928	1.0243265	0.75593084	0.28146361	2.64977885	0.54583836	1.60735826	1.12484922	0.41314837	0.72275834	0.55991154	0.495577	4.39987937	0.7247688		
NC	65_PB_T	0.57133051	3.99123825	0.42193667	1.71903863	2.22172873	1.01648379	1.74225524	1.00235194	0.34219266	2.31863284	0.25639214	1.01850263	2.95860377	1.22745213		
NC	73_PB_T	0.3209417	1.30791287	0.37627647	1.7103476	6.50736959	0.92559988	2.22445797	1.00306856	1.16303637	2.1691232	0.05533478	0.69017556	3.58770562	0.42054429		
NC	74_PB_T	0.37320189	1.1377125	0.5170506	0.75445126	1.76239815	0.93853737	2.19293834	1.08138014	0.22734131	1.64872749	0.12473594	0.51302686	1.07333266	1.06327331		
NC	75_PB_T	0.17985792	1.89302975	0.54057856	0.06832591	2.12111773	0.59282779	1.95030295	1.14345428	0.96862032	1.013836	0.08339781	0.16177165	0.17684354	0.00401917		
NC	86_PB_T	0.17054342	2.02633836	0.79620566	2.21807357	4.22523841	0.82647964	1.20894092	1.27049801	0.99702306	0.94454816	0.26943842	0.64685403	0.96069428	0.19980826		
CAS	102_PB_T	0.22315595	1.75006534	2.25870007	0.58201484	2.49090288	0.80215516	1.9320078	1.74302889	0.95293621	1.26455037	0.53678052	2.07977323	3.11915723	0.73078547		
CAS	103_PB_T	0.10650483	0.89725298	0.30243353	0.1477001	1.80656311	0.6309909	0.42601933	1.27504371	0.6068766	0.85002914	0.72543858	0.89825774	1.49609147	0.21401443		

CAS	104_PB_T	0.2590543	1.71258077	0.93138589	0.40420535	4.98150332	0.92735392	1.28519157	2.55727922	0.8688903	1.01101737	0.09475138	1.30232746	1.47066235	0.14716703		
CAS	10_PB_T	0.3583022	3.75247545	0.52979727	0.9483269	1.79865662	1.17290378	6.0441804	1.37093975	1.04632408	1.51691472	0.29399155	1.6639105	1.68840979	0.1541414		
CAS	115_PB_T	0.20673033	2.51605942	0.86625052	0.3075744	2.09553967	0.68775652	2.14898702	1.7365348	0.50926253	1.18895152	0.0695824	0.26320301	0.63935137	0.15529986		
CAS	116_PB_T	0.17233213	2.38644724	1.46431919	0.13302831	1.25469882	0.81026334	1.33129088	1.63362794	0.74274139	0.96344745	0.44745886	1.25772219	1.46633477	0.79917764		
CAS	120_PB_T	0.21632826	1.04642505	0.68319482	0.92367134	2.07172036	0.37329201	0.95486286	1.54750166	0.79588674	0.76670758	0.13080313	5.25828588	0.46284185	0.04628418		
CAS	121_PB_T	0.35199903	3.29305684	1.51692419	2.04542704	2.32077299	0.8472183	3.40803647	0.76249647	0.40041151	1.30612821	0.16944366	1.55525074	3.11151007	0.52144269		
CAS	124_PB_T	0.28557638	1.75368032	1.1061057	0.33585391	3.50736063	0.8718124	1.09202799	5.60795592	0.42233127	1.43592631	0.7903628	0.31272625	0.5500362	0.11966053		
CAS	126_PB_T	0.31016552	1.66536586	0.77642742	0.82609445	6.69389906	0.759196	0.56863679	2.8543337	1.30857415	2.13973666	0.09021154	0.15710999	1.18694061	0.03243561		
CAS	128_PB_T	0.29089665	1.93058743	1.10319282	4.36143657	2.65933889	1.10017313	1.08708781	2.97539961	0.09059065	3.16463341	0.11877441	0.97334622	4.90196078	0.39155293		
CAS	19_PB_T	0.31176145	2.57152914	0.74822749	0.70196611	2.07170513	0.67682406	2.48001207	0.58731835	0.45959672	2.22658018	0.13174436	1.29129582	1.23497762	0.48071605		
CAS	1_PB_T	0.32139717	2.45292744	0.8105677	0.12128195	2.07998545	0.54677946	1.26840706	1.40585994	1.20069131	1.11983667	0.43257229	0.66502936	1.65751999	0.04244868		
CAS	20_PB_T	0.14041704	0.97288948	0.32496515	0.4031975	1.4934355	0.19959279	1.14439886	0.25275067	0.47541197	0.38915579	0.13941406	0.25876854	1.64488531	0.56768603		
CAS	23_PB_T	0.32549656	2.73195409	0.4887487	0.55425111	3.91704373	0.73866556	4.2637026	1.28586257	1.10749448	1.47330021	0.13201254	1.01276793	0.97145103	0.51394193		
CAS	28_PB_T	0.11965932	1.44496174	0.41528824	3.47313698	1.00554053	0.4142827	2.59328902	0.65159026	0.88789229	1.18653782	0.14982554	1.64305322	2.00002011	0.16390311		
CAS	29_PB_T	0.20044521	1.47966841	0.47442057	0.44118091	2.34289225	0.73731605	2.51311959	0.64867696	0.60838647	1.39908742	0.38175244	0.3908178	1.14324278	1.5683075		
CAS	30_PB_T	0.25033699	3.88377066	2.008777	0.28479634	4.29018821	1.8780342	7.85368968	2.53884277	1.56485958	4.25066132	1.3966169	2.08073621	1.09763143	0.14391843		
CAS	31_PB_T	0.16408955	0.5546831	0.46206813	0.33019248	1.36808408	0.66743175	2.71301442	0.76608682	1.01775791	1.16171378	0.20435693	0.42079407	1.54526053	0.21744383		
CAS	32_PB_T	0.18811363	1.02305649	0.23237566	1.19406889	1.12063415	0.74943667	2.37908418	0.67499598	0.15894093	1.49786737	0.05331563	0.33900692	0.69310317	0.6699662		
CAS	35_PB_T	0.13028855	1.19481674	0.56155377	3.0451162	0.10301886	0.29188676	1.45438386	0.22421751	0.06463928	1.56548262	0.05150943	3.39457232	6.47200816	3.29256345		
CAS	37_PB_T	0.30015766	3.59279622	0.92472814	1.29866192	6.56405385	1.36940615	2.70141893	1.68573392	3.42705259	2.15668836	0.22031774	0.55989004	3.58774306	0.76100578		
CAS	3_PB_T	0.13999678	1.58730159	1.12299573	0.21452744	3.66509548	0.61538152	2.03649988	1.86830231	0.87724599	1.09882362	0.27395053	1.51679961	1.0857304	0.2437354		
CAS	46_PB_T	0.22367082	2.12185022	0.6417942	1.2140691	6.43103987	0.79796077	3.39334831	0.33651376	1.47602591	0.62869636	0.49469537	0.77982529	1.59390648	0.88964565		
CAS	47_PB_T	0.28300082	5.41931455	2.1391236	0.26890113	5.04164443	1.14711007	6.47276243	1.3072422	1.54190124	2.24889972	0.61937901	0.26789401	1.22566545	0.51363137		
CAS	48_PB_T	0.39915331	2.40701542	0.86382421	2.77189799	3.93105534	0.99284346	3.79195646	0.87692773	0.32960387	1.10674327	0.14917851	6.58401371	6.01249874	0.15119444		
CAS	49_PB_T	0.55508543	3.36274984	1.19983075	0.38886202	3.37685364	0.96409574	3.6226628	0.60545616	2.04908124	1.18371212	0.76160542	1.07591876	0.57321889	0.04634107		
CAS	52_PB_T	0.17485328	1.38877723	0.61600611	2.91221159	1.41892435	0.36076051	0.8441193	1.01796768	0.75166814	0.6401238	0.38487821	2.04799421	2.87402524	1.08429134		
CAS	57_PB_T	0.22057046	1.00314237	0.60833132	1.88643139	1.34155185	0.7795504	1.40298928	1.11795987	0.17625494	2.09390863	0.29308678	1.85621626	9.4200709	0.8178229		
CAS	58_PB_T	0.23320333	2.11189739	0.68654256	0.0834305	1.51280608	1.10470025	4.19866511	1.47159342	0.58903944	1.38916811	0.07739938	0.14374171	0.33673757	0.03618672		
CAS	63_PB_T	0.22571999	1.3271126	1.40168081	0.24486588	3.36463855	0.57739979	1.06511619	1.39361938	1.58407062	1.47322598	0.42020194	0.78195852	2.26526129	1.35331224		
CAS	77_PB_T	0.15322272	2.19350416	0.46672446	1.23787827	3.66827282	1.2590472	4.67329288	2.92534425	0.24394669	1.68544989	0.22479385	2.00298381	2.10882845	0.65422068		
CAS	78_PB_T	0.1872433	1.86035274	1.14460014	0.35133285	3.33413868	1.04896513	4.03781107	1.53116695	0.85366836	3.18011597	0.02214706	1.46774583	1.66606266	0.08758154		

CAS	79_PB_T	0.3558539	2.36460871	1.63066005	1.9480979	1.80049941	0.78146325	2.11086062	1.96528403	0.44279547	1.37590101	0.63790855	1.58921116	2.42021088	0.74608005		
CAS	83_PB_T	0.3390142	1.73489498	1.67464848	1.6756696	0.86387354	1.01500036	2.75500097	2.1086275	0.18992964	2.44559945	0.01531691	0.52588047	1.18042295	0.120493		
CAS	87_PB_T	0.30554519	4.02149909	1.26755876	0.83394678	1.76570835	1.16671877	1.46318836	2.41713473	0.1189912	1.03663517	0.21075559	0.74419918	2.66923473	0.57377958		
CAS	90_PB_T	0.40329492	2.24030328	3.20014519	1.00924554	4.8899509	2.19896555	2.87045159	3.22434288	0.78340038	2.93397054	0.17240858	0.62309065	0.96387486	0.20164746		
CAS	9_PB_T	0.29030502	2.58956112	0.64310626	0.20160071	3.77497329	0.89510715	3.81025341	0.23184082	2.68532145	1.36080479	0.51508981	1.32350866	6.72741568	0.63201822		
ASCVD	101_PB_T	0.18362138	3.82275493	1.65259239	2.26802668	4.08305336	1.16024496	1.78072379	2.5152093	0.52765923	1.19757458	0.08575724	6.92414016	3.09936741	0.94433851		
ASCVD	111_PB_T	0.32049667	2.74841011	0.86977555	1.71334697	2.68491549	0.4313603	2.31402626	2.07214199	0.40616402	2.66778202	1.24167263	0.94133298	0.91109745	3.84495218		
ASCVD	112_PB_T	0.52753448	0.69868116	0.77720729	0.16812645	2.3145072	1.33091714	1.18091211	2.88835196	0.29799658	1.31178899	0.15503876	0.63022249	0.6906272	0.6594181		
ASCVD	114_PB_T	0.10658301	1.1271656	0.40622203	2.3538757	1.34435361	0.51280504	0.95723608	0.83557057	0.41024403	1.2126331	0.30969403	0.34790303	2.87573024	0.45549154		
ASCVD	118_PB_T	0.25755564	1.64694756	0.61068856	0.55937865	2.89750091	0.77970945	0.76461829	1.55941889	0.58956095	1.01513139	0.56843334	0.84812266	1.21936496	0.32194455		
ASCVD	11_PB_T	0.34164433	2.55275835	1.73543225	1.29905468	3.04254933	1.05113579	6.61721726	0.89694233	1.47340415	3.14736057	0.99066777	0.65406244	2.75028722	0.36180033		
ASCVD	127_PB_T	0.43318523	5.3449794	0.97669099	1.30967683	4.8996488	1.27728915	0.82588585	3.50191796	0.66799591	3.35414917	0.32792527	0.26213779	2.05358137	0.33602219		
ASCVD	129_PB_T	0.10735427	0.49162235	0.40333099	0.1745761	1.09360891	0.3210595	0.19765225	0.49764222	0.36520518	0.26186415	0.02407946	0.26788402	1.43975118	0.33510585		
ASCVD	12_PB_T	0.22448158	0.25971411	0.28588685	0.98651097	1.18179988	1.03784981	2.20656332	0.0825448	0.01912623	0.74290316	0.11677069	0.60700624	1.72941413	0.13489028		
ASCVD	133_PB_T	0.23902454	1.83857271	0.84515849	1.22437042	1.68729136	1.05392676	1.13158452	3.36449729	0.1906145	1.15276391	0.02017085	0.44880135	1.94144403	0.06252963		
ASCVD	13_PB_T	0.24254919	1.72917361	0.5043002	2.47096989	3.69887518	0.92572942	4.765081	0.3597813	0.95402682	1.5230068	0.24052795	1.29561693	1.8080021	0.26377225		
ASCVD	14_PB_T	0.43232022	1.47734602	0.49278459	0.12495969	2.83174782	0.68526282	3.14313931	0.33154628	1.21936472	1.59525153	0.09875847	0.30332957	2.27043696	0.17836988		
ASCVD	15_PB_T	0.35553855	1.47352094	0.35352416	0.39683339	2.89869669	0.76445823	3.03265314	0.07352497	0.80575308	1.92373547	0.29007111	0.72618496	1.35567955	0.05035957		
ASCVD	21_PB_T	0.22895014	0.85454637	0.56936286	0.47798363	1.13872571	0.53220867	1.63578852	0.35748356	0.36953356	1.17085907	0.25505849	0.16769594	0.60450871	0.08736255		
ASCVD	22_PB_T	0.26653524	4.11268909	1.304514	1.06915835	2.74983907	0.61152237	3.54642742	1.13654651	0.50390248	0.92231252	0.08549244	2.24090763	1.96129707	0.19814129		
ASCVD	25_PB_T	0.29490806	3.26713839	0.32409691	0.40763741	3.43723894	0.79313156	4.36826266	0.92498465	0.93907582	1.64665385	0.0583777	0.32610993	1.3356416	0.26773223		
ASCVD	34_PB_T	0.30095234	4.23959038	1.29874065	0.09493128	4.99500096	1.01596663	3.02164231	0.47869601	1.55626698	0.50293378	0.10604025	0.34740807	0.30095234	0.01514861		
ASCVD	36_PB_T	0.24719625	1.9502163	0.88443575	3.05044222	3.31992665	1.25117773	2.4283992	2.25110681	0.85100347	2.5985999	0.19451508	0.33229659	3.20848572	3.22570841		
ASCVD	4_PB_T	0.24375749	1.75767282	1.00927689	1.17245339	4.05725279	1.02035677	2.28245651	2.06186605	0.21656141	1.69220077	0.06245027	0.44118091	1.02136403	0.1712346		
ASCVD	54_PB_T	0.18992684	0.37985369	0.36779484	0.57982957	0.3316183	0.53360399	1.03304124	1.05816384	0.18289252	1.2521103	0.01004904	1.88620468	0.63208457	0.10752472		
ASCVD	59_PB_T	0.1337342	1.54146263	0.7380519	0.49471599	2.14980242	0.82955425	2.30666358	0.6636434	0.2674684	1.44292164	0.05329258	0.22121447	1.68726307	0.10457411		
ASCVD	62_PB_T	0.21915935	2.22376371	2.82594927	0.50668034	1.73216314	0.58811111	2.13227976	1.89502468	1.67687065	1.61554624	0.29757417	1.43659961	0.45943039	0.38101557		
ASCVD	69_PB_T	0.33569225	2.56860017	0.66130366	0.61291558	1.58168511	0.80041936	3.20369362	1.54640215	0.78630618	1.76112422	1.37502772	2.14218029	2.10286498	0.42339563		
ASCVD	6_PB_T	0.28247452	1.92203301	0.49558696	1.49178713	2.80765596	0.75594604	1.72198878	1.87780213	0.80922415	0.89265968	0.10555098	2.2718591	1.29073765	0.73885683		
ASCVD	70_PB_T	0.27060469	4.42021186	2.07128271	0.15592463	4.72602533	1.65380708	3.57821884	2.30567264	0.33800437	1.97370407	0.14787691	0.35611174	1.24236724	0.08550706		
ASCVD	71_PB_T	0.27477781	0.78205993	0.56565982	4.02806156	2.78300605	0.64316125	2.75985627	1.52889193	1.13735871	1.68993387	0.51030165	1.01657725	0.99040794	0.11071633		

ASCVD	72_PB_T	0.139662	1.24891988	0.26827161	0.10550007	0.96859112	0.46520507	1.84373932	0.94347206	0.26525732	1.20471033	0.42501457	0.62094327	1.09318168	0.18085726		
ASCVD	81_PB_T	0.40884481	2.99752062	0.44325254	2.67671912	0.21454233	0.9229368	2.67165916	2.22334666	0.09816323	3.62900369	0.3208015	0.63856702	5.75115114	0.71345444		
ASCVD	84_PB_T	0.26196929	0.29308441	0.19572418	0.75479273	0.96055405	0.33022182	0.70259962	0.75880759	0.32921811	0.59319482	0.04918197	0.9645689	0.88427181	0.33122553		
ASCVD	88_PB_T	0.27081923	2.60916137	0.88824665	1.32175952	3.35593529	1.55013693	1.18938146	2.460615	0.39511313	1.98567083	0.05861013	0.44058651	0.97515133	0.24454572		
ASCVD	91_PB_T	0.42447646	2.25748883	0.65637572	1.33896613	3.65392565	1.27040461	2.7888406	3.43009246	1.18369446	1.39845333	0.56563253	0.93364657	1.35812303	1.18167794		
ASCVD	99_PB_T	0.30652342	0.82309076	0.78389596	1.47030743	1.82004563	0.7175663	3.06623921	1.97380983	0.58390199	1.97581982	1.21302875	2.27028331	2.54163191	0.71354633		

Table S5. Frequencies of CD45+ immune cell clusters identified by myeloid cell panel.

Group	Sample	C01	C02	C03	C04	C05	C06	C07	C08	C09	C10	C11	C12	C13	C14
NC	107_PB_M	1.39834814	0.44767261	0.04929429	5.26845266	0.07042041	0.44968462	1.3410058	0.1539189	0.1026126	0.35713208	0.10864863	0.43459453	1.15489472	0.69716206
NC	117_PB_M	1.09330327	0.19530463	0.02919502	5.08899448	0.02315467	1.23525148	4.90577055	0.25973503	0.15402891	3.2839367	0.19127774	0.32819232	3.81146056	0.21745258
NC	119_PB_M	0.59870599	0.52927622	0.28576891	5.20622654	0.09458548	0.76171502	2.83857075	0.16502148	0.41355994	1.50733038	0.66310462	0.01811211	0.15294674	0.43368451
NC	130_PB_M	0.78512189	0.38904247	0.03619	3.59487308	0	0.87459161	3.45513948	0.1035436	0.018095	1.71198794	0.1578286	0.01608444	0.0452375	0.20608193
NC	131_PB_M	0.85610072	0.28838135	0.08741874	4.8261171	0.00401925	1.40171421	3.27267612	0.10450056	0.05526472	0.66920549	0.08942836	0.00100481	0.00502407	0.20297224
NC	132_PB_M	0.57248068	0.47086285	0.08551996	4.38767708	0	3.67735834	0.9246217	0.13280747	0.01408564	0.78678364	0.19619285	0.02112846	0.32900032	2.6863329
NC	24_PB_M	0.85823108	0.41605113	0.0683369	2.79678817	0.00602973	3.76154441	3.68818274	0.16380757	0.03818827	1.89232918	0.14471344	0.00803964	0.26631292	0.3637935
NC	56_PB_M	0.84119655	0.37442281	0.08733186	3.40092351	0.00200763	0.38044569	1.12025698	0.0762899	0.1596065	0.17466372	0.21883156	0.09235093	0.58422004	1.75266011
NC	65_PB_M	1.75327419	0.70211439	0.09857767	6.61778019	0.00201179	1.06121874	1.08334842	0.18910817	0.05934778	2.95230048	0.12372503	0.01106484	0.27863279	1.02903012
NC	73_PB_M	0.6943887	0.38231516	0.2418319	2.98025207	0.00702416	3.09163523	4.64598218	0.19366621	0.02408284	1.00846913	0.04816569	0.08228305	2.67620615	0.14851088
NC	74_PB_M	1.12192376	0.48154254	0.225189	3.30243687	0.00301592	2.66909281	6.88334406	0.22116777	0.0502654	3.14460351	0.06735564	0.17291298	5.85792987	0.19704037
NC	75_PB_M	1.35361751	0.18376261	0.17974595	4.81297384	0.0030125	1.30541748	2.27343475	0.22593764	1.85068032	0.39865442	0.1104584	0.03414169	0.32936687	1.17387157
NC	86_PB_M	0.92818907	0.14846985	0.06968993	4.19452581	0	0.83122917	6.52863347	0.24946975	0.04948995	0.96454904	0.14442986	0.00605999	0.05049995	0.16462984
CAS	102_PB_M	0.31296227	0.19321144	0.07144798	3.23025369	0.02616405	0.21635656	0.35120204	0.07245429	0.0945931	0.80202872	0.63900657	0.04930917	0.31396858	0.4840349
CAS	103_PB_M	0.61839318	0.10239627	0.03413209	1.34620983	0.00301166	1.71965507	1.96661078	0.1003885	0.00803108	0.16463715	0.08533023	0.45275215	1.17052995	0.45877546
CAS	104_PB_M	0.41070855	0.27012371	0.07631748	3.48047879	0	1.01421915	3.43629499	0.08033419	0.00803342	4.12415649	0.1466099	0.01907937	0.06727988	0.0532214
CAS	10_PB_M	0.83792412	0.29105778	0.04229905	3.89453436	0.01208544	1.08265437	1.70001914	0.14401821	0.04431329	1.84806583	0.06143434	0.00100712	0.27695809	0.13696837
CAS	115_PB_M	1.25275899	0.22374296	0.05341611	2.53071426	0.01713347	0.80325737	7.62036262	0.30235535	0.01814132	0.36383427	0.10884793	0.05039256	2.92579192	0.55129458
CAS	116_PB_M	0.40425017	0.22581556	0.038308	3.5717166	0.00806484	0.79237066	1.84080003	0.11089157	0.08064841	0.34779628	0.16432114	0.05141336	2.10290738	0.4496149
CAS	120_PB_M	0.2943836	0.14970361	0.23711444	1.60856023	0	0.93338692	0.68220637	0.07736361	0.02109917	0.17381694	0.42298804	0.00502361	0.02411333	0.39284638
CAS	121_PB_M	1.43560915	0.34152386	0.0715286	4.68764167	0.0110819	1.47086973	2.0521655	0.1118264	0.01611912	1.54239832	0.29618884	0.00705212	0.67498816	0.21861557
CAS	124_PB_M	1.31075421	0.30433604	0.03515433	5.60661303	0.00200882	2.28302247	4.02667711	0.21293478	0.41682988	0.49617822	0.3113669	0.01004409	0.11249385	1.83907353
CAS	126_PB_M	2.17411126	0.35458478	0.20059368	3.55293951	0.0070917	1.65743058	4.80006484	0.24719625	0.21173777	3.41819729	0.37180747	0	0.00810479	0.12359812
CAS	128_PB_M	1.41555909	0.23324553	0.06635433	4.37938592	0.00804295	3.11061066	4.27281684	0.14276235	0.2825086	2.05095208	0.17895562	0.00201074	0.01709127	0.32573945
CAS	19_PB_M	0.80147037	0.41379172	0.10646098	5.52391857	0.00903914	1.29360129	1.91027148	0.19484367	0.33846556	18.9028493	0.32741772	0.00903914	0.08938705	0.02912612
CAS	1_PB_M	0.69910402	0.35859215	0.17276709	3.76973764	0	1.08079875	0.62879184	0.12053518	0.04720961	1.46751577	0.64888103	0.06930773	1.20736068	32.9141388
CAS	20_PB_M	0.88273015	0.12524548	0.12724941	2.88164803	0.00300589	0.90878121	0.45689552	0.18636528	0.01502946	0.38375215	0.10320228	0.03506873	0.09218067	0.11121799
CAS	23_PB_M	1.01304928	0.25175779	0.08124455	3.24476675	0.00902717	1.60081846	3.83654801	0.2156491	0.02507548	1.06520627	0.06920832	0.62086882	3.76533365	1.60884262
CAS	28_PB_M	0.56793097	0.1123821	0.07124222	1.14489263	0.00501706	1.29941802	2.45334136	0.09833434	0.329119	0.09231387	0.51675697	0.12241622	3.4166165	0.35119406

CAS	29_PB_M	0.34844605	0.21690014	0.13455842	4.3259527	0.02912085	1.52030928	0.49003364	0.08836672	0.01004167	3.05768941	0.09941256	0.18275845	0.24501682	0.13154592
CAS	30_PB_M	1.20330532	0.22819575	0.13169006	2.26486791	0.00402107	1.96529816	8.93280791	0.40411758	0.0371949	2.45787929	0.06031606	0.00100527	0.04121597	0.05931079
CAS	31_PB_M	1.19542307	0.14353106	0.09133795	4.50968584	0.13650507	0.7086219	1.01575831	0.1756499	0.08933052	0.63635451	0.10137509	0.01505571	0.3211884	0.13449764
CAS	32_PB_M	0.62946119	0.13954562	0.10139646	3.64625686	0.01807066	2.00584285	1.26494594	0.10742001	0.05220412	0.33029144	0.08834543	0.00100393	0.03312954	0.17568694
CAS	35_PB_M	0.78506174	0.11645417	0.15259512	3.43037848	0.01204698	0.30820199	1.8803333	0.2037948	0.16263427	1.66951109	0.32526855	0.02810963	0.04818793	0.13552856
CAS	37_PB_M	0.3754953	0.22923884	0.06431135	0.20226957	0.00311184	1.47916105	4.41881211	0.25102172	0.252059	1.06424911	0.30703483	54.5744041	2.35669979	0.05808767
CAS	3_PB_M	0.44620923	0.16444565	0.07620652	1.41683964	0	1.64445648	3.2628423	0.14639674	0.00401087	0.32588314	0.19753532	0.02105706	0.28076086	1.74573093
CAS	46_PB_M	1.58843244	0.20369664	0.44552369	1.94164041	0.02307893	0.87800277	1.31650244	0.12141524	0.03210982	2.0108772	0.07124365	0.1705834	3.15980654	0.30303638
CAS	47_PB_M	0.75784226	0.30253385	0.12865227	3.0172977	0.0010051	1.28953796	1.48955203	0.22715166	0.25730453	2.06145156	0.07035671	0	0.03216307	0.08844843
CAS	48_PB_M	1.71230127	0.41352176	0.34125582	4.0097559	0.02910711	0.78890316	1.31082383	0.14553557	0.10137305	0.70057813	0.07025855	0.00501847	0.03814036	0.00401477
CAS	49_PB_M	0.5491637	0.56823886	0.24396121	4.31399715	0.00200791	7.00861394	7.9724113	0.30219063	0.31323414	2.68357328	0.0722848	0.00100396	0.06023734	0.13754192
CAS	52_PB_M	0.69819933	0.21969203	0.22370467	3.94141546	0.0100316	1.87189647	2.27616994	0.11034759	0.07824648	1.2649847	0.24176155	0.35411546	0.43135878	0.54170638
CAS	57_PB_M	1.24045802	0.23202089	0.05423865	3.34069908	0	2.66773805	6.16613098	0.18983528	0.02209723	0.67195661	0.17075131	0.0050221	0.02410607	0.21092808
CAS	58_PB_M	0.69621396	0.15750085	0.09429987	2.32138199	0.00401276	3.30350515	0.69119801	0.0611946	0.04514356	2.42772015	0.1123573	0.08025521	0.41331434	2.19197047
CAS	63_PB_M	0.99332095	0.28122332	0.20288254	2.61638126	0.00903932	1.62808216	1.6300909	0.16069904	0.04419224	0.29829759	0.03615728	0.46401848	1.53568021	0.30934565
CAS	77_PB_M	1.12287262	0.18697789	0.13269399	3.60585864	0.00100526	1.18821436	4.02706153	0.47448154	0.02915247	0.61220182	0.0542839	0.02111041	0.29454045	0.15279914
CAS	78_PB_M	0.40258215	0.16364312	0.20279699	2.59017941	0.00803156	1.55209975	2.66547532	0.14757999	0.06124068	1.21276617	0.07328802	0.00301184	0.22086801	0.55618581
CAS	79_PB_M	1.15705138	0.29956673	0.0733838	2.0316254	0.0070368	0.77605879	2.83884717	0.19200418	0.01608412	2.96752013	0.19300944	0.00301577	0.04121556	0.11862038
CAS	83_PB_M	1.61115873	0.35406718	0.02959063	4.90286111	0	6.86604628	3.45292029	0.20509367	0.06020162	4.13248439	0.17346231	0.0214277	0.16019754	0.09489409
CAS	87_PB_M	1.92534381	0.35967961	0.07556294	5.26623344	0.01108257	2.12079996	1.30472017	0.12493073	0.22870384	1.42763589	0.07556294	0.20653871	0.45237016	0.2035162
CAS	90_PB_M	0.75109346	0.37604947	0.05630687	3.53325625	0.00201096	2.26434066	3.45683978	0.24131517	0.07138907	1.37549646	0.07440551	0.00402192	0.04826303	0.20210145
CAS	9_PB_M	0.65728736	0.31108257	0.14349938	4.54080199	0.00200698	1.06871914	2.8649701	0.23682415	0.21474732	2.07622526	0.06322001	0.01806286	2.15349416	0.14249589
ASCVD	101_PB_M	1.00147957	0.25062152	0.01509768	3.36074401	0.01711071	0.65322637	2.69644601	0.13386611	0.04529305	3.22788441	0.20130243	0.00503256	0.02616932	0.03623444
ASCVD	111_PB_M	0.67862385	0.36695956	0.19403615	6.63945469	0.00100537	0.36092735	1.3381457	0.17895562	0.02010737	1.17225987	0.13069793	0.00100537	0.03820401	0.1186335
ASCVD	112_PB_M	1.18586203	0.46189376	0.03413997	4.27352144	0.0120494	5.11798373	5.21036249	0.13957225	0.11246109	1.44090772	0.31027212	0.00100412	0.0893664	0.13957225
ASCVD	114_PB_M	0.88332831	0.18189127	0.07436439	5.53612702	0.01708371	0.95166315	1.30539644	0.1205909	0.09546779	0.41402874	0.22007838	0.15073862	2.53140388	0.26730982
ASCVD	118_PB_M	0.62068758	0.24706981	0.21995239	4.04752579	0.06226963	0.55038316	0.78740948	0.06327398	0.03816526	0.83561823	0.42483956	0.086374	0.36256993	1.24940995
ASCVD	11_PB_M	1.2031388	0.30304247	0.11439351	3.18194589	0.00602071	4.92795216	3.3214257	0.11439351	0.03612427	0.63317813	0.08328651	0.00100345	0.750582	0.03411736
ASCVD	127_PB_M	1.00594236	0.42995923	0.42286085	2.24308922	0	0.64291074	2.77952421	0.1166163	0.00507027	1.91250735	0.43604356	0	0.00405622	0.26771047
ASCVD	129_PB_M	0.33904444	0.15392819	0.03823053	1.58857913	0.0030182	0.53422135	1.20929203	0.05231546	0.04627906	1.37629908	0.28773504	0.00201213	0.0211274	0.39639023
ASCVD	12_PB_M	1.82616806	0.21962153	0.02607379	5.13453072	0.01504257	1.01487209	0.92862802	0.11632921	0.13638597	7.97456803	0.1133207	0.05315042	8.77884413	0.07621569

ASCVD	133_PB_M	1.44220615	0.23232191	0.08347497	5.97097485	0.00704006	1.9078557	11.6905191	0.43648359	0.02614879	0.88905874	0.14381833	0.00402289	0.05933763	0.11465237
ASCVD	13_PB_M	0.44398461	0.16674535	0.12957922	4.49308409	0.01406286	1.26867096	0.67501733	0.05223348	0.14263759	1.66042209	0.13962412	1.20840155	43.4311372	0.34554458
ASCVD	14_PB_M	0.90201272	0.39030361	0.13043566	5.34184175	0.01304357	1.16890414	2.16924528	0.16555295	0.05418096	0.90702948	0.10836193	0.01003351	0.64013806	0.15852949
ASCVD	15_PB_M	1.08956287	0.33007936	0.2136988	4.61208151	0	4.20976593	1.15276956	0.1093576	0.05518044	1.01933322	0.25383002	0.02708858	9.70072136	0.14848555
ASCVD	21_PB_M	1.20404559	0.20468775	0.04715845	3.40544229	0.04615508	3.3000883	4.67972387	0.1364585	0.05117194	3.84190881	0.14147536	0.59098571	3.30510515	0.03210788
ASCVD	22_PB_M	0.70383646	0.30322198	0.08835607	3.63565168	0	1.11850759	2.69988052	0.12952197	0.0411659	0.39860638	0.0542185	0.00602428	0.13755434	0.02710925
ASCVD	25_PB_M	1.51845124	0.33921779	0.07326301	3.36407704	0.01605765	2.9746791	0.72359772	0.18165213	0.22179625	1.78741683	0.1023675	0.0341225	0.63427705	3.12020152
ASCVD	34_PB_M	0.8	0.23849057	0.05232704	0.83522013	10.3768553	0.86037736	1.4963522	0.14188679	0.12880503	1.02742138	0.10163522	0.00503145	0.24955975	0.01610063
ASCVD	36_PB_M	0.43628267	0.21061922	0.11734499	2.63073436	0	3.25958317	2.49633924	0.19557499	0.10230076	0.88259483	0.16949833	0.00501474	0.00300885	0.32997011
ASCVD	4_PB_M	1.12418196	0.22282892	0.04918296	3.82422612	0.00401494	2.67796202	9.02055647	0.34026579	0.15959369	1.40422371	0.21981772	0.02609708	2.39189786	0.19070944
ASCVD	54_PB_M	0.52669068	0.27688881	0.15549915	4.4542983	0.0100322	0.39727525	0.5868839	0.07122864	0.01705475	1.74760983	0.22973746	0.01203864	0.02909339	0.39025271
ASCVD	59_PB_M	0.57462619	0.16747395	0.08624407	2.61339591	0.00701987	2.80995216	4.58096413	0.23666978	0.15243138	2.48703832	0.05515609	7.1933572	3.97926131	0.45227995
ASCVD	62_PB_M	0.54443186	0.21957749	0.16944564	2.74923048	0	1.25028826	1.3164623	0.05013185	0.04511866	1.12495864	0.10527688	0.0080211	0.03408966	0.27472252
ASCVD	69_PB_M	0.73461523	0.2870218	0.14451447	3.42820441	0.00200715	3.03681105	4.78704187	0.21476456	0.0551965	1.03769419	0.11942515	0	0.0441572	0.06121794
ASCVD	6_PB_M	0.99925683	0.29626208	0.0461968	3.58728182	0	1.28748468	6.33799988	0.20688131	0.06025669	1.5726997	0.04920963	0	0.43987386	0.03414546
ASCVD	70_PB_M	0.94463817	0.20199178	0.06431579	2.0289622	0	2.56459215	1.26521219	0.1115477	0.05627632	2.02795727	0.08139967	0.00200987	0.03316283	0.03617763
ASCVD	71_PB_M	0.65112919	0.26787595	0.1043412	3.56967283	0.01103609	1.38653397	2.2904899	0.10634776	0.03310826	0.59795531	0.37322043	0.30800718	0.96515606	0.35315482
ASCVD	72_PB_M	0.6724767	0.14474835	0.08845732	4.07104731	0.00301559	1.32887026	3.52220982	0.1909874	0.21611733	1.94606114	0.14776394	0.07538977	1.26755325	1.03334238
ASCVD	81_PB_M	2.27295625	0.60148709	0.07153364	5.01843754	0.00201503	1.60094304	2.54700062	0.41711165	0.00906764	6.18614867	0.15314244	0.00503758	0.02720293	0.05541338
ASCVD	84_PB_M	0.96310119	0.2638496	0.08627781	4.96498726	0.02207107	1.24801862	1.72555629	0.11637473	0.06420675	0.67918698	0.27689159	0.68520636	2.54118261	0.37821786
ASCVD	88_PB_M	0.77679122	0.30303949	0.0191925	3.62637252	0.00303039	4.01325293	0.63537279	0.12525632	0.115155	6.91132055	0.10707395	0.32829278	0.6899199	0.68890976
ASCVD	91_PB_M	1.25099225	0.37379046	0.08641392	6.10624893	0.00301444	2.08900634	3.06166538	0.21402518	0.17383266	2.7481637	0.05225028	0.01406738	0.02512033	0.2461792
ASCVD	99_PB_M	1.67049824	0.30117156	0.02610153	4.21238618	0.03413278	2.16040397	2.55493871	0.19776932	0.06123822	2.3019546	0.17267169	0.01305077	0.01807029	0.04818745
**Group**	**Sample**	**C15**	**C16**	**C17**	**C18**	**C19**	**C20**	**C21**	**C22**	**C23**	**C24**	**C25**	**C26**	**C27**	**C28**
NC	107_PB_M	0.44566059	1.33899379	0.62975967	0.35813808	0.1368168	0.24747744	3.42243192	0.04225225	1.08045029	6.41529934	34.0945444	0.16498496	1.24241723	1.31082563
NC	117_PB_M	0.67953932	0.6422905	0.20033826	0.57786011	0.0362421	0.04228245	0.59698788	0.00906052	0.28993678	5.13631055	37.1934523	0.05134297	0.19027101	0.44396569
NC	119_PB_M	0.7788209	1.18835592	1.62405289	1.36947706	1.02031575	0.19822703	5.27666254	0.04528028	4.04000765	26.8572464	2.51054024	0.1126976	0.70737867	1.1380445
NC	130_PB_M	0.04724805	0.14978638	0.51068108	0.11862277	0.83538578	0.61623523	0.53380246	0.12968082	1.52500628	10.9223423	0.14274943	0.02010555	2.65091732	0.47248052
NC	131_PB_M	0.02913958	0.15273158	0.24416957	0.07636579	0.68427769	0.65212367	1.68808593	0.01909145	4.43624964	1.80363943	0.2079963	0.11354388	3.73790456	0.7546146
NC	132_PB_M	0.95882968	2.82115261	0.85721185	2.57566001	1.4045396	0.386349	2.2647698	0.01207341	2.81008532	1.05541693	2.5162991	0.08350773	5.89081616	6.96736156
NC	24_PB_M	6.15132604	3.79671782	1.95664627	8.47478067	4.00574834	0.62508165	0.98184047	0.01507432	1.77876933	2.26114746	0.04723286	0.0572824	0.72055232	2.40988071

NC	56_PB_M	0.95261996	4.04838386	1.89821321	1.67737402	2.19032323	1.17847822	1.41437462	0.03212206	7.89600482	2.70728769	1.09616543	0.41959446	15.8853644	7.92812688
NC	65_PB_M	3.62524393	3.34761704	1.46659424	3.13436739	1.16884946	1.55813065	0.85199268	0.00704126	2.07113686	3.21986843	0.40336371	0.17703744	15.4907759	11.4430563
NC	73_PB_M	15.5153729	18.078189	2.8106687	2.37015333	1.85538252	0.44352573	0.18062134	0.05017259	0.86296861	0.24985952	0.06221402	0.1896524	0.1605523	1.77209601
NC	74_PB_M	13.4490108	6.53249156	1.65574232	3.00788161	0.26138009	0.41117098	0.16487052	0.03216986	0.99927618	3.89757922	1.51097796	0.13873251	0.18497668	2.07193984
NC	75_PB_M	8.83566802	4.86418637	2.16699302	10.0456896	3.07978109	3.30772707	1.14173821	0.02811668	2.02841793	7.70999649	0.18476678	0.07732088	0.42175026	5.14434905
NC	86_PB_M	1.63619836	0.89384911	0.87667912	1.35440865	0.98979901	7.32552267	2.45530754	0.00908999	5.74790425	4.83890516	0.07069993	0.07170993	20.0636299	5.6034744
CAS	102_PB_M	1.22568505	2.49766033	6.79762108	2.03878317	5.10802733	2.1363952	3.6730299	0.25560263	21.7715074	2.45338271	0.31094965	0.39346704	4.24260111	2.49665402
CAS	103_PB_M	0.38348408	2.77273047	2.66431088	1.59316555	3.56078022	0.54912511	0.27707227	0.04216317	49.5186371	1.2207242	0.91654704	0.47383374	2.00375453	2.34607933
CAS	104_PB_M	0.53120983	1.86174486	3.76265263	0.50008033	9.28462404	0.2751446	0.81037114	0.05924647	29.5639862	1.72517674	0.03916292	0.51514299	1.9932921	1.84768638
CAS	10_PB_M	9.16882358	11.2042138	12.5376411	2.41708882	5.13127814	0.26990825	1.68692657	0.0100712	1.96187042	0.60930781	0.01712105	0.53075242	1.70001914	4.18156366
CAS	115_PB_M	7.7302184	8.57882908	12.2846978	4.10497778	1.23764123	0.22172726	6.87757632	0.07558884	1.65892301	2.23843743	3.19488818	0.16327189	0.50594128	2.17191925
CAS	116_PB_M	4.57679745	7.22710593	8.55578854	2.83277552	1.87709182	0.38207186	0.65426025	0.20363724	2.25613936	5.3288439	1.639179	0.2338804	1.58474132	4.24311464
CAS	120_PB_M	1.61860746	1.43172913	2.99608158	1.93911384	3.39596102	0.6249372	2.22947855	0.08439666	4.54134432	24.1304129	0.19491611	0.08339194	1.44177635	2.61830604
CAS	121_PB_M	9.50725864	4.55465893	7.60721734	2.40880104	0.79588156	0.47752894	2.64252828	0.03022335	0.4251418	7.05513747	0.5974149	0.05540948	0.57928089	1.85168394
CAS	124_PB_M	0.15769227	0.35656532	1.04960778	0.88588905	0.49216058	0.68299836	6.79583371	0.01707496	5.42682376	11.0675867	5.78338908	0.06829984	6.22432479	1.48351262
CAS	126_PB_M	0.25631414	0.98068019	1.49026918	0.08408725	1.69592835	0.05065497	2.12750869	0.04660257	1.7901466	2.01100226	0.01114409	0.07294315	7.00862148	0.59367623
CAS	128_PB_M	0.1186335	0.57708162	0.8022842	0.17694489	0.61327489	0.32071261	2.12233326	0.04122012	1.92729174	3.2634267	0.45543201	0.11963887	1.49699395	1.47889731
CAS	19_PB_M	3.262125	2.46366768	3.29727721	1.47337973	4.38800004	0.48710918	2.61733305	0.19082628	6.11548003	5.18947041	0.03013046	0.20990891	2.21458917	4.83393092
CAS	1_PB_M	1.55791715	2.16963317	1.18626703	16.4108642	0.92812086	1.29474868	0.69910402	0	0.83169272	0.5845956	0.56551087	0.03816947	0.46205151	1.84217928
CAS	20_PB_M	2.00192377	0.82261232	1.01599134	1.10015631	0.76149253	64.6717566	0.13626708	0.01302553	0.90276943	1.78850547	0.01001964	0.08917478	1.59412448	1.10717005
CAS	23_PB_M	5.05722224	5.60487066	0.98095267	20.8708212	4.24878885	0.39819858	0.35807781	0.02206642	1.01806437	0.94384096	0.262791	0.03510567	0.31996309	5.66204275
CAS	28_PB_M	7.46136865	4.24142083	1.8252057	30.4966887	1.7298816	0.34818383	0.26690749	0.017058	0.32811559	0.59903672	0.13646398	0.02408188	0.38631347	2.93297211
CAS	29_PB_M	2.25134307	6.94281267	2.71526836	3.97951499	18.7990159	1.65888437	1.46809258	0.09640006	10.3830898	1.03730481	0.05020836	0.17572928	2.06055129	4.22553597
CAS	30_PB_M	1.26965298	1.67377056	2.70517512	2.5785114	7.6329969	0.45538622	2.80670715	0.12264265	5.87478387	2.10402509	0.01608428	0.13269532	6.5563553	5.10575415
CAS	31_PB_M	7.42045569	7.94339055	11.8799558	1.61497541	3.22493225	0.61427281	4.03894409	0.05721168	11.8909967	1.98333835	0.1756499	1.27270902	2.54842919	3.60032119
CAS	32_PB_M	5.29068658	3.48964451	3.08305474	4.16026664	7.21822325	1.62535514	1.68257888	0.02007851	8.36169422	5.02866207	0.02007851	0.28009517	5.19129798	11.0441828
CAS	35_PB_M	2.44252585	4.46240337	1.71970686	1.84921193	6.56259412	2.19957836	3.90824214	0.12448549	4.90312218	2.38128702	0.07529365	0.18572432	9.54522638	9.72492722
CAS	37_PB_M	0.05082671	0.12447358	0.025932	0.31222123	0.09542975	0.09335519	0.04252847	0.03319295	0.4937452	0.35682426	0.33919051	0.01452192	0.12551086	0.08609423
CAS	3_PB_M	2.0615869	13.4795295	2.36641298	2.06660049	3.31297817	1.49705702	0.11230434	0.00401087	4.99453519	0.11029891	0.05314402	0.39607336	3.00213579	4.62052161
CAS	46_PB_M	9.80854522	18.7701941	3.3926027	4.11607698	3.10461779	0.32511188	0.14148387	0.0220755	0.84990668	0.26791627	0.13847358	0.11138092	0.81679343	6.20923559
CAS	47_PB_M	8.34129034	12.7767783	8.19655654	1.60312786	6.70197903	0.8925251	0.97494296	0.00502548	2.42429116	0.37791603	0.00301529	0.20403445	0.19096821	1.81721327

CAS	48_PB_M	25.0441625	4.51260639	3.29111129	0.82905091	4.1663321	0.52995022	0.41753653	0.07728441	0.93243135	0.63533804	0.00100369	0.13750602	0.28605267	3.12550185
CAS	49_PB_M	15.6456438	11.1188081	4.61417987	1.12643817	1.23386141	0.40158223	0.31022228	0.00200791	1.1686043	0.60638917	0.04317009	0.46583539	0.16364476	1.69568098
CAS	52_PB_M	0.88980288	0.50258314	0.39524502	5.20238752	1.85283643	4.7620003	2.73160455	0.05617696	13.9549581	3.51607564	1.25595626	0.100316	16.4417916	5.83237197
CAS	57_PB_M	1.11591	3.02430695	1.83708317	1.28264363	5.61269586	0.81960627	1.32282041	0.01607071	10.2591402	1.96564885	0.02008839	0.16472479	7.44073925	5.74929691
CAS	58_PB_M	2.31335647	4.80929355	0.733332	4.75612448	2.82398026	1.09648683	2.08362593	0.00902871	7.4817921	0.733332	3.96761702	0.34409422	10.0339078	15.5825525
CAS	63_PB_M	0.99733842	3.49419977	2.0479084	2.806207	4.58092703	0.83663938	1.3870336	0.28021895	19.7137548	1.00838648	0.42886557	0.26314468	6.79756943	4.4362979
CAS	77_PB_M	13.8504378	5.22130744	2.4266916	4.59201624	2.6488535	3.30930768	2.2256401	0.03719453	1.10477799	2.29299235	0.06132071	0.08343637	0.60617027	4.42313299
CAS	78_PB_M	11.5132471	11.5534049	4.99764073	4.47659301	3.60316042	0.91760619	1.52499322	0.03112231	1.85428735	0.8162077	0.08332748	0.30519943	3.01284046	8.27853464
CAS	79_PB_M	1.88686832	3.57067463	2.53123838	1.02335213	3.58776401	1.15303035	1.95924686	0.04423133	7.16245966	2.71419524	0.03116298	0.29755622	21.0470762	8.75880857
CAS	83_PB_M	2.6815232	1.86012816	1.44177789	1.51218318	1.86318926	1.60707726	3.08354761	0.03673319	4.80184482	11.8913514	0.11121995	0.14489205	3.85698543	4.58246602
CAS	87_PB_M	1.8044431	1.22714221	1.17172938	1.40647826	1.25434487	4.36451564	2.39282656	0.07959297	8.24643595	1.77421792	0.32643192	0.09571306	20.9218679	3.86781522
CAS	90_PB_M	7.00316726	3.03152179	2.71379016	6.50646021	8.87134885	1.45895128	1.48609924	0.01709316	4.29842642	3.20446433	0.04223015	0.11663566	0.99341411	7.60444422
CAS	9_PB_M	9.23915225	14.9450086	7.11576285	1.13093566	0.92823024	0.59105688	0.68137117	0.01103841	1.28246297	1.40990647	0.04214667	0.9462931	1.06269819	3.27238791
ASCVD	101_PB_M	1.46447515	1.69597294	3.17957183	0.48010629	2.81924049	0.36335088	8.87844353	0.11474238	10.4174006	3.45133011	0.07246887	0.51936026	5.11207513	2.6209576
ASCVD	111_PB_M	0.55395814	0.89578348	1.39947319	1.03452436	2.07005409	0.17996099	0.57306014	0.12164961	2.9809181	6.10158245	0.12164961	0.07741339	25.3302636	4.22154304
ASCVD	112_PB_M	0.73099709	1.83753389	2.96415303	0.44984436	2.81052314	0.06928406	0.29119389	0.55728487	3.27241691	2.91997188	0.08635405	0.24600864	22.4410081	2.23817652
ASCVD	114_PB_M	3.39061401	4.82866044	3.68304693	0.95065823	0.44317154	0.67430409	1.12250025	0.1748568	2.05105015	5.22259069	3.03788564	0.32459049	1.07526882	1.5325093
ASCVD	118_PB_M	0.74723553	2.30498057	1.88918015	2.52191991	3.66888628	0.20589151	6.99026786	0.17776974	2.56510691	5.86640152	2.38733717	0.0883827	2.8774594	2.3632328
ASCVD	11_PB_M	10.10175	7.33021594	12.6525247	1.0696797	2.22565626	0.14349362	0.54286746	0.05518985	1.32857028	0.854941	0.00903107	0.39837039	0.50172594	1.23725616
ASCVD	127_PB_M	0.16529093	0.65710751	0.78792058	0.18760014	1.14081165	0.17441743	1.12154461	0.02535137	1.68637313	0.81225789	0.03346381	0.04157625	30.7593242	1.09416513
ASCVD	129_PB_M	0.47184523	1.64994919	1.03725465	0.54629415	3.39245651	0.26057124	0.55333662	0.06036399	3.49608137	3.06548487	0.14990392	0.17404952	4.26471624	2.86024729
ASCVD	12_PB_M	13.4470552	15.9902524	12.0581245	0.92060531	0.28781452	0.284806	0.4773509	0.04613055	1.09810764	0.43924306	0.21460734	1.30669796	0.21059599	1.00785222
ASCVD	133_PB_M	0.84380123	2.00239362	2.90553248	0.29165954	0.64869105	0.2272933	8.81616397	0.07542919	1.90282709	1.99736501	0.45760377	0.38016313	11.4471342	2.75769126
ASCVD	13_PB_M	1.53686981	2.39872229	2.84471588	1.71868251	0.13861963	0.17578576	0.43092624	0.06428736	1.18228481	0.77044388	1.51979348	0.13259269	0.47914176	0.73227326
ASCVD	14_PB_M	14.3348785	10.6405394	13.9104609	2.4772741	2.0618867	0.78863404	1.82609917	0.01404692	2.8164068	1.01438806	0.05117091	0.92007304	0.34715951	2.12108442
ASCVD	15_PB_M	8.39043673	5.460857	16.2190362	2.3215916	0.74543758	0.44144352	0.58591595	0.06521325	1.48485548	0.68825058	0.03611811	0.15049211	0.19563974	0.61200124
ASCVD	21_PB_M	4.10077862	2.33986194	6.44766415	4.83424306	4.58239685	1.28230856	1.43181088	7.51926473	8.75441483	4.50614063	0.29599454	0.23679563	4.26031466	3.74859528
ASCVD	22_PB_M	8.10767393	1.70185849	5.00617488	2.24906373	2.21191401	1.32333303	0.34237979	0.28816129	6.85060795	11.9812846	0.04919827	0.25904395	3.6165748	7.11768427
ASCVD	25_PB_M	3.02084483	1.32877029	0.48775103	32.4715729	1.23443161	0.50280507	0.99657771	0.00401441	0.69248602	0.24989713	0.10838912	0.01505404	0.18566654	2.10756616
ASCVD	34_PB_M	1.02037736	0.45383648	0.82716981	0.31194969	0.04930818	0.08352201	51.2362264	0.01408805	0.14490566	1.88679245	0.25056604	0.05433962	0.12176101	0.10867925
ASCVD	36_PB_M	1.87350811	1.33693058	0.13940987	2.25563156	5.82713177	1.85244619	0.31091409	0.0020059	3.64471546	1.27575071	0.02707961	0.0742182	0.83645919	11.6592783

ASCVD	4_PB_M	8.10414743	6.83743526	20.1609989	1.26269723	0.61729634	0.87625969	5.18428554	0.05420163	2.44108082	2.8787088	0.2930903	0.29007909	0.63636729	1.49355603
ASCVD	54_PB_M	1.13865508	3.10496694	1.49279186	2.54015389	9.78842083	1.81181593	4.83853169	0.01906119	16.4427813	2.59733745	0.05216746	0.21268271	1.60113966	5.12846236
ASCVD	59_PB_M	1.25555322	2.08991446	0.55356659	3.20507035	2.21827773	2.73072796	0.93263937	0.32391668	10.9429686	3.76365113	2.38073749	0.14942287	1.57044436	3.78170222
ASCVD	62_PB_M	1.12295337	1.68543269	0.46321826	2.1255903	4.73846216	1.26332254	0.4912921	0.02406329	3.02896618	3.80099662	0.04010548	0.1032716	29.0614316	12.590112
ASCVD	69_PB_M	2.87724299	4.77399542	5.86487897	1.78134158	18.5570631	0.15354663	1.25647304	0.06222151	10.0006021	0.94235478	0.00301072	0.14852876	2.39051022	2.63136767
ASCVD	6_PB_M	4.01610862	2.83909454	43.1899894	0.43886958	0.82852954	0.78534557	0.3414546	0.03615402	1.01030389	1.98043666	0.0090385	0.18780003	0.17675297	0.22797116
ASCVD	70_PB_M	4.62269744	2.02393753	0.46729442	1.18280759	1.78978786	0.37685034	0.44317599	0.02009868	1.45715463	4.24986685	0.02411842	0.11657237	18.014451	21.8462652
ASCVD	71_PB_M	2.30052271	3.27771814	1.65240336	4.64017337	8.02925567	0.29596781	0.17457085	0.16253148	16.5892468	1.51696046	0.17356757	0.17657741	1.39255365	5.75381497
ASCVD	72_PB_M	5.05915584	5.06619221	3.4970799	6.36591176	2.90602414	6.52372767	0.47947891	0.02814551	3.37846667	2.49188304	0.07639496	0.06131701	4.32837771	4.53142748
ASCVD	81_PB_M	1.94249098	0.89064421	0.24986399	0.96217785	1.24629738	0.86948637	0.60753219	0.08463135	1.53746952	11.3436234	0.17631531	0.09067645	8.12057952	12.2393052
ASCVD	84_PB_M	2.77292883	1.93422821	4.2205903	3.94169225	2.46092418	4.95395172	1.9502799	0.49559582	17.1141074	9.9921748	0.80158109	0.11737796	6.44274564	3.82632075
ASCVD	88_PB_M	1.82631797	2.81523683	1.03437478	1.42832611	1.13437781	2.43542734	2.10612443	0.06262816	6.51635908	2.78695314	0.94851359	0.24142146	2.62230169	3.33747487
ASCVD	91_PB_M	1.51324846	2.84864501	0.27129952	1.41980085	2.99836215	0.8470574	0.93749058	0.00200963	2.66978829	0.89026437	0.04220215	0.08038504	3.74895751	11.7422454
ASCVD	99_PB_M	2.89425867	1.51489293	0.56419472	0.72080393	2.59409101	0.4838823	3.51768379	0.0200781	5.83469697	14.2444108	0.18672637	0.36542149	2.29793898	7.50117959
**Group**	**Sample**	**C29**	**C30**	**C31**	**C32**	**C33**	**C34**	**C35**	**C36**	**C37**	**C38**	**C39**	**C40**	**C41**	**C42**
NC	107_PB_M	2.00396366	1.11465449	2.07337807	3.55522469	2.62466928	0.16599097	0.03018018	0.01609609	0.28972969	4.62662093	1.69511987	0.81285273	0.85309297	2.03414384
NC	117_PB_M	0.10469939	0.47517416	0.02315467	1.06410824	18.7502517	1.21813716	0.040269	0.05335642	0.07248419	1.11746466	0.38255547	0.16510289	0.040269	1.13055209
NC	119_PB_M	1.62606534	3.27124903	5.99510973	3.52783731	6.05145853	0.37632948	0.17407754	0.01509343	0.13785331	3.91121039	1.96818305	0.54436965	0.11571628	2.83353961
NC	130_PB_M	1.42548379	1.13495853	49.0575521	2.0789143	1.08771048	0.03317416	0.02312139	0.01507917	0.10253833	1.10379492	2.87811008	0.09550138	0.35486303	1.05855743
NC	131_PB_M	12.4827926	1.58157575	38.2451945	1.28415108	0.31350167	0.01607701	0.00200963	0.00602888	0.15775565	1.44492117	1.79660574	0.14368827	0.03918771	1.11433768
NC	132_PB_M	4.92293142	18.6111558	5.91194462	2.32714907	0.6318416	0.67409852	0.12978912	0.17707663	0.10866066	2.88655023	1.70838699	0.91556665	0.03923857	1.37838055
NC	24_PB_M	1.35065875	0.33766469	0.66427488	0.81300813	2.76060981	9.15211995	4.60068136	0.36881827	0.20601566	0.72356719	3.3384586	4.26100676	0.21807511	3.87108445
NC	56_PB_M	13.8897812	2.23147962	3.91186509	3.05962658	2.26360169	0.50491869	0.16261795	0.14454929	0.0833166	1.62316804	1.16944389	0.36940373	0.17265609	1.59405742
NC	65_PB_M	3.83044642	3.77713401	0.65684914	1.58730159	1.85788722	1.66777315	0.69507313	0.56631863	0.14987829	2.08924296	0.87412236	0.76045627	0.08751283	1.26340354
NC	73_PB_M	0.65224372	0.65124027	0.26390784	1.28843221	0.40639801	6.38697118	5.13064943	0.31809424	0.14349362	4.63394076	2.32198764	2.17648712	0.0541864	1.09777635
NC	74_PB_M	0.42222937	1.04049381	0.05428663	1.95733473	7.92283256	13.1313334	2.08199292	0.33376226	0.09952549	1.38933569	0.72884832	0.6132379	0.12767412	2.092046
NC	75_PB_M	2.74137671	1.79344279	0.46894613	2.11075965	8.97625144	6.79017924	2.22824723	0.24602099	0.1134709	1.89687202	0.98910478	0.54526284	0.02108751	1.18792991
NC	86_PB_M	10.9342491	1.83213817	0.83728916	1.41197859	0.51004949	0.22421978	0.27774972	0.06362994	0.23330977	3.11584688	1.96343804	0.55650944	0.13937986	0.81203919
CAS	102_PB_M	6.11634951	2.02670746	2.56005152	4.49719743	0.41459954	0.38843549	0.7788836	0.09861834	0.21333763	2.32356878	1.22971028	0.2948487	0.20428084	1.04958087
CAS	103_PB_M	6.98703984	1.38536135	0.3021694	2.75365665	0.97678014	0.20880809	0.20579643	0.01505828	0.08533023	1.27292622	0.96674129	0.23792075	0.14355556	1.1404134
CAS	104_PB_M	10.0949952	1.35965617	1.95613753	1.26827603	1.2783178	0.14761407	0.3996626	0.01405848	0.12150546	2.92918541	2.00333387	0.62359415	0.05121305	1.5715376

CAS	10_PB_M	1.00611322	0.56700875	0.594201	0.68484183	0.80569627	2.46945908	8.59275075	1.07359028	0.2326448	0.39076269	1.39284743	2.15926601	0.08359099	1.8319519
CAS	115_PB_M	0.14815412	1.2134528	0.74278631	6.46637305	0.90101894	3.99209845	2.62545227	0.35677931	0.10985578	2.73026879	0.68836234	0.25297064	0.05341611	1.62969533
CAS	116_PB_M	0.61897657	3.40638735	4.31166579	17.7003105	2.76523247	2.4396145	0.90830275	0.25505061	0.09980241	1.55853059	0.75103835	0.29134239	0.35586112	1.52324691
CAS	120_PB_M	1.87380689	3.29649352	21.8386416	3.36280518	0.90324525	0.26022305	0.40088416	0.018085	0.05525972	1.6839144	1.40761579	0.40691249	0.0994675	1.27800663
CAS	121_PB_M	0.3646951	2.5689848	3.31449411	6.46578213	3.86355165	3.65098075	4.87804878	0.22667513	0.21156345	2.85711407	0.72133063	0.39794078	0.04130525	1.55751
CAS	124_PB_M	2.91278714	9.05274154	8.01518667	3.49835779	2.07711855	0.11148944	0.03615874	0.05725133	0.13559526	2.12231697	1.02550195	0.18782455	0.14363054	2.98912225
CAS	126_PB_M	1.52471456	0.59468933	39.6891811	1.23699434	0.54606056	0.07496935	0.1560173	0.01823579	0.1671614	4.10913106	3.4070532	0.60380723	0.04356327	1.85194566
CAS	128_PB_M	3.94305592	1.88406089	34.6017735	3.05531538	0.16488046	0.02211811	0.01407516	0.00201074	0.13371403	1.51710132	0.80630567	0.12768182	0.09551002	1.48794563
CAS	19_PB_M	2.69768096	1.94341499	6.43184991	2.85837677	1.05657497	0.54435707	1.22430122	0.09541314	0.09340444	0.56042665	1.04753583	1.4211536	0.18379584	1.49045366
CAS	1_PB_M	1.16115553	0.67399253	0.22299008	0.76640283	0.57555547	8.95877697	0.23805697	0.15870465	0.11149504	0.65088995	1.20736068	2.1113745	0.02310258	1.06070955
CAS	20_PB_M	0.63524508	0.36972466	0.28856559	0.92381067	1.48992024	4.86653842	0.95587351	0.25550078	0.11522584	0.55508797	0.7123963	0.75848663	0.06111979	0.56210172
CAS	23_PB_M	0.82247565	0.28987252	0.16248909	0.36008385	2.38116731	10.3972959	2.91176441	0.45537067	0.19659174	1.05517608	2.27685333	2.71717871	0.0421268	1.91376042
CAS	28_PB_M	0.15251856	0.32209512	0.22877784	0.48665463	0.96327514	14.7190448	5.20670279	1.31045555	0.15151515	0.80674293	1.48203893	1.40878989	0.05619105	1.58137668
CAS	29_PB_M	9.51046844	1.27529246	3.07978109	2.16297635	0.57639203	0.87864638	2.63794748	0.17070844	0.16870011	0.41472109	1.03529648	1.61068434	0.17372094	1.47010092
CAS	30_PB_M	3.37367807	2.5041216	3.38976235	0.86955648	0.47448631	0.24126422	1.06357312	0.18396397	0.39808597	1.93815594	2.10503036	1.58128594	0.66749769	5.7280148
CAS	31_PB_M	3.51801666	3.63545117	1.2345679	5.86871424	1.0739737	1.67319081	4.3390545	0.23486902	0.22583559	0.39747064	0.87724581	1.2415939	0.09936766	1.32590585
CAS	32_PB_M	7.12586212	5.69225672	2.15944342	5.40914978	0.99288217	1.11536106	1.53801363	0.20781255	0.15661235	0.33631499	0.52505296	0.53910791	0.10641609	1.76289291
CAS	35_PB_M	8.9549242	0.76799518	6.23130208	1.19265134	0.54311816	0.41060135	0.96175083	0.11344243	0.08734063	0.23090051	0.32727638	0.62644313	0.11444634	1.0450758
CAS	37_PB_M	0.23961164	0.03526751	0.0311184	0.06431135	1.86295459	1.01134784	0.06846047	0.02178288	0.23546252	0.53108728	2.76227621	7.5368753	0.06119951	1.49886936
CAS	3_PB_M	28.2525644	0.97063041	1.82394289	0.85431519	0.2426576	1.00171465	0.46526086	0.04411956	0.12533967	0.55851357	2.10670918	2.71736406	0.02005435	1.8650543
CAS	46_PB_M	0.47060948	0.3381565	0.13847358	0.49870557	0.71645026	6.61863573	8.95663168	1.91254089	0.21975155	1.81721487	2.05904192	4.07794658	0.10235004	1.53424713
CAS	47_PB_M	1.51166414	1.10661052	0.65733268	1.44030233	0.91865759	2.076528	8.1814801	0.2160956	0.33369182	1.72172917	2.31976119	4.57218096	0.27338607	2.29865418
CAS	48_PB_M	0.58816445	0.81198812	0.80797334	0.78689578	0.28003051	2.59354424	8.64481291	0.21177935	0.23988277	1.09402602	1.79661153	3.49787217	0.01003694	2.08768267
CAS	49_PB_M	0.82826336	1.34028071	0.09236391	0.92163123	1.84627432	5.23261651	6.35805072	0.21083067	0.18874365	1.39148244	1.63945947	3.86121318	0.05622151	1.8784009
CAS	52_PB_M	6.91177208	1.54687265	0.24878367	0.82459748	1.25595626	0.68114561	0.16652455	0.09329388	0.08827808	0.7864774	0.72829413	1.03827055	0.17856247	1.14360235
CAS	57_PB_M	11.2746083	3.54760948	5.59562073	0.9351145	0.49216553	0.19184411	0.31438329	0.06227401	0.09240659	0.90699076	0.60265167	0.20992366	0.06428284	0.692045
CAS	58_PB_M	9.60855521	3.84623101	0.48353765	1.47067675	0.44842599	0.65708954	0.21969864	0.10433178	0.14144981	0.98613591	0.77646917	0.54673863	0.02507975	2.55412211
CAS	63_PB_M	10.2345202	3.04725556	3.35760558	2.86345603	0.8004821	0.58353839	0.65083112	0.09139758	0.14362477	2.96590167	1.81188169	0.90192337	0.07030583	1.21629087
CAS	77_PB_M	1.35106608	2.27891875	0.46141319	3.56263257	1.03843099	8.56077284	4.13060305	0.41718186	0.16486223	2.55134353	1.35307659	0.99218915	0.24628809	3.2922183
CAS	78_PB_M	2.71266076	1.38042507	0.49795697	1.59426546	0.72986838	3.80696136	4.41133655	0.95575612	0.10742217	2.75583041	1.34729487	1.08928087	0.05923278	1.62036805
CAS	79_PB_M	5.27257557	2.00247293	2.66091659	1.54005448	0.46442896	0.26639324	0.47046051	0.22417242	0.1749148	1.19223539	0.87055299	0.569981	0.12364667	2.22161907

CAS	83_PB_M	5.68038039	2.85600588	2.35806702	2.08256806	6.30994653	0.86118934	0.70609363	0.07652749	0.24590833	1.01832578	0.38569854	0.12652545	0.06428309	1.95094078
CAS	87_PB_M	13.0794418	2.92982721	1.05082867	0.88358269	0.22769634	0.2035162	0.42819002	0.07153292	0.20754622	1.17172938	0.75260692	0.21963629	0.09369805	1.59790439
CAS	90_PB_M	4.0098537	1.06480318	1.38052386	2.14368307	1.03765522	1.89130763	1.86114323	0.22824393	0.23829873	3.70720426	2.27439546	0.6143482	0.09149867	2.71982304
CAS	9_PB_M	0.99044675	0.68939911	0.2177578	2.54284911	4.36820134	4.39328864	3.37073014	0.49973909	0.28398828	0.31308955	2.46357323	4.53478104	0.14349938	1.24934773
ASCVD	101_PB_M	5.89514157	2.80313629	4.98324157	1.6013608	0.6632915	0.13990519	0.57471843	0.04831258	0.1489638	2.99135406	1.65470595	0.42072207	0.01409117	0.93806931
ASCVD	111_PB_M	1.04558342	2.75672089	9.93605855	1.45275773	3.12669656	0.22721332	0.2503368	0.1508053	0.03820401	2.04894135	0.84853116	0.30663744	1.30195243	3.80934189
ASCVD	112_PB_M	0.99708806	0.71794357	20.7601165	2.97620243	0.83140878	0.18576162	0.34240386	0.11647756	0.15965458	1.61763229	0.73400944	0.34441209	0.06225525	1.30434783
ASCVD	114_PB_M	0.99286504	2.08421264	3.06099889	34.5111044	2.53743342	1.34056879	0.36378253	0.08642348	0.07034469	1.24309115	0.49241282	0.23012763	0.06632499	1.05517033
ASCVD	118_PB_M	1.67525385	2.4003937	32.2084626	4.04149969	0.90592265	0.28121767	0.16672191	0.02611307	0.16772625	1.84197576	1.43621883	0.40676128	0.19082628	1.37897094
ASCVD	11_PB_M	0.26491129	0.45155334	0.30203901	0.60809184	2.04503492	4.40314683	12.1648471	0.52580878	0.17259372	0.76161997	2.07513848	3.73183752	0.09532793	2.22565626
ASCVD	127_PB_M	2.03317987	0.52325228	23.2492344	0.47356359	0.43198734	0.09126493	0.074026	0.0537449	0.22309206	2.25120165	2.19847081	0.24033099	0.02433732	1.25844201
ASCVD	129_PB_M	6.76177349	0.98292705	53.4764631	2.62683984	1.14792197	0.15996459	0.09960059	0.0181092	0.04627906	0.88332646	0.68211314	0.14487359	0.00704247	0.24044991
ASCVD	12_PB_M	0.44225157	0.94266775	0.21962153	4.49873141	0.60671701	4.46864627	4.6040294	0.22263004	0.16446544	0.30285709	0.6197539	0.70599797	0.0080227	0.52147578
ASCVD	133_PB_M	1.54177269	3.6910018	16.6376683	4.82746829	0.17097284	0.05430902	0.11565809	0.05531474	0.15286983	1.09422615	0.57326186	0.16192133	0.0533033	2.69533646
ASCVD	13_PB_M	0.23906864	0.31139192	0.04218858	0.77345735	1.48061836	5.03651321	1.26565749	0.36161643	0.13761514	1.06074151	2.61669663	2.8929314	0.05524695	2.12750997
ASCVD	14_PB_M	1.12375334	0.9210764	0.3391327	3.39835049	0.74147653	4.61039873	7.96359842	0.39431702	0.12240885	0.89298256	1.62141553	1.87325668	0.01204021	0.65819838
ASCVD	15_PB_M	0.16955444	0.39529261	0.12340353	1.3012551	0.76650648	10.3508473	11.0942783	0.53775847	0.1354429	0.79961474	1.82296108	2.13899451	0.04615091	0.92402155
ASCVD	21_PB_M	1.07059721	1.74486274	2.73719698	3.54992776	0.37526088	1.88332798	4.2974394	0.32709905	0.11639107	0.42843956	0.72242736	0.6090464	0.0943169	0.8327982
ASCVD	22_PB_M	1.85045734	3.40070484	1.84443307	4.12361818	1.50506541	1.97295099	3.3153609	0.29117343	0.13554625	0.60845206	1.60647409	1.75306485	0.05321445	1.78117815
ASCVD	25_PB_M	0.20874941	0.36330426	0.04716934	0.29204845	0.99858492	18.739274	1.38999006	0.25993316	0.1013639	1.38898646	2.21695888	2.24305256	0.01103963	1.67300609
ASCVD	34_PB_M	0.07245283	0.32301887	0.08754717	0.5272956	4.99522013	1.22163522	0.65308176	0.05333333	0.24654088	1.58289308	2.5881761	2.40201258	0.01308176	1.25786164
ASCVD	36_PB_M	28.3072232	1.24165045	1.21958558	0.78530881	0.78530881	0.39415883	0.21563396	0.01002949	0.13238922	1.60371492	1.46330211	2.12625118	0.06117987	1.61173851
ASCVD	4_PB_M	0.27703055	1.33396234	0.21781025	2.91584695	0.66146063	1.47247762	2.68097322	0.27301562	0.14253021	0.60525154	1.22756655	2.58160357	0.07929498	1.3801341
ASCVD	54_PB_M	19.8236339	2.61639864	4.12724847	2.86319084	0.89085966	0.31501119	0.39627203	0.02909339	0.09129305	0.23074068	0.26384695	0.27086949	0.03410949	0.76044102
ASCVD	59_PB_M	13.3277174	1.96155119	2.74777621	6.26372635	1.13721833	0.99280965	0.21962153	0.05415325	0.18552504	1.09209062	0.89052017	0.31188263	0.02707663	0.97676424
ASCVD	62_PB_M	5.78421248	0.89334951	1.7455909	0.314828	3.10416395	0.55445823	0.27171461	0.36696512	0.08021095	1.95614466	1.30743856	0.47926045	0.18548783	2.30807022
ASCVD	69_PB_M	2.86118582	0.69647947	4.4990165	1.05274778	0.51583638	0.7025009	4.47894504	0.12845731	0.09533941	1.80141303	1.06980852	0.55698286	0.26393963	3.80956204
ASCVD	6_PB_M	0.11448772	0.71002471	0.3735915	1.51143873	1.1207745	2.18330086	4.99628417	0.15365457	0.09741499	1.02335951	1.10571033	1.54357563	0.06226525	1.17098841
ASCVD	70_PB_M	2.83089972	0.68134541	0.48839803	2.05609543	2.11739642	0.65320725	0.51251646	0.32559869	0.19495724	3.11328624	1.80385694	0.79992764	0.0381875	1.83902964
ASCVD	71_PB_M	14.488377	1.59922948	1.88115136	3.80745036	0.5568208	0.74142446	1.07451366	0.06521325	0.10133135	2.17210278	1.42365535	0.86482799	0.28693829	1.80088891
ASCVD	72_PB_M	5.03503111	2.35718666	1.67365278	2.22249027	1.70682428	13.7953218	2.65271453	1.05244112	0.06835339	1.01926962	0.56089985	0.20003418	0.31864741	1.43140034

ASCVD	81_PB_M	4.2194773	4.5267697	2.45430915	1.11431277	2.9530296	0.2881496	0.10276664	0.06851109	0.13097709	0.27404437	0.1753078	0.10881174	0.12493199	3.35200596
ASCVD	84_PB_M	4.41421377	3.05784626	1.26908646	4.13531572	1.55801681	1.37241919	1.41957102	0.23676237	0.06320352	0.7293485	0.39125986	0.1384458	0.06019382	0.56080579
ASCVD	88_PB_M	27.5877047	0.73739608	3.29403921	1.77884178	0.81315595	0.38688041	0.19899593	0.0222229	0.21515804	1.79298363	1.19397558	0.31213067	0.03535461	1.44751861
ASCVD	91_PB_M	26.3793571	0.95557721	3.3791863	0.61494559	0.56269531	0.23311663	0.08239467	0.01306257	0.27330915	2.85768833	1.7624421	0.50140171	0.2773284	2.15833844
ASCVD	99_PB_M	12.8570138	2.1473532	0.95170212	0.87339752	5.31567799	0.3865035	0.40256598	0.02911325	0.13351939	1.69057634	0.72783126	0.16865607	0.70674925	3.34701991

Group	Sample	C43	C44	C45	C46	C47	C48	C49	C50	C51	C52	C53	C54		
NC	107_PB_M	0.83800288	2.15687655	0.57342334	2.03716186	0.06539038	1.4255103	1.01505991	2.77154613	0.95067553	0.36417412	1.889279	1.05328813		
NC	117_PB_M	0.13288769	0.93826763	0.80034631	1.27954738	0.0241614	0.89598518	0.60302823	1.47082511	0.3070511	0.33020577	1.33592397	0.33725285		
NC	119_PB_M	0.54436965	0.13885954	0.38639177	0.73555307	0.03924291	0.97805416	0.81806382	1.56166672	1.02635313	0.73454684	1.02937181	0.92371781		
NC	130_PB_M	0.40713747	0.4644383	0.84945966	1.69288766	0.00201056	0.66549384	1.21940186	0.51972858	0.70972606	0.25634582	1.68585072	1.00929882		
NC	131_PB_M	0.69131138	1.16658796	1.86091378	3.58316335	0.00602888	0.95256278	1.50621477	1.23391043	0.82897077	0.58279157	1.53334472	0.96261091		
NC	132_PB_M	0.55738892	0.79181423	1.63997102	3.59586285	0.06942209	1.33813587	0.8250161	1.48402286	1.06346587	0.71635544	1.65305055	0.9537991		
NC	24_PB_M	0.53966053	1.91242827	2.7585999	1.01701388	2.25009296	1.62400635	2.77769403	1.08334087	0.66427488	0.41404122	4.02685238	0.5115218		
NC	56_PB_M	0.10540052	1.2397109	3.11383256	0.8452118	0.0481831	0.38546477	0.78498294	1.01284883	0.4226059	0.57418189	0.97269625	0.72475407		
NC	65_PB_M	0.20520249	2.28941598	1.53298328	0.87211057	0.26957974	1.83877522	1.38612268	2.69579737	0.60051904	0.53312411	3.61116141	0.37519866		
NC	73_PB_M	0.239825	0.83386851	4.17837361	0.89608252	0.2940114	0.67833347	1.22822509	1.8473549	0.81781328	0.9382275	1.53829172	0.15754194		
NC	74_PB_M	0.58006273	1.27975712	1.19430594	0.51270709	0.18497668	0.78615088	0.86959144	1.21742802	0.51773363	0.54588226	1.35012868	0.2503217		
NC	75_PB_M	0.0582417	0.01907918	0.24702515	0.17572928	0.08033338	0.46693779	0.88567555	1.50625094	0.32936687	0.37857107	2.14590551	0.32635437		
NC	86_PB_M	0.26966973	0.5958994	1.02817897	2.23209777	0.03736996	0.83425917	0.7989092	1.1968488	0.66255934	0.30703969	2.07756792	0.78072922		
CAS	102_PB_M	0.22038179	2.51376128	2.1887233	1.42895958	0.01509464	0.70039145	1.23776076	2.16457187	0.81410443	0.42566894	1.87576102	1.96029102		
CAS	103_PB_M	0.2750645	0.83924789	1.42250509	0.55615231	0.01806993	0.53707849	0.48788813	0.78905364	0.26602953	0.47082208	0.80310803	0.30016163		
CAS	104_PB_M	0.22493573	0.54928503	1.3094473	1.13070373	0.03514621	0.33639942	1.15179145	1.87479916	0.54225578	0.56736022	1.50626607	0.99614396		
CAS	10_PB_M	0.12991852	1.14811719	1.53082292	0.21350951	2.76555246	1.48147402	2.61851289	1.28407843	0.45924688	0.40687662	4.03855257	0.23868752		
CAS	115_PB_M	0.11791859	0.16831114	0.63897764	0.42833674	0.05845537	0.48981566	0.90908175	2.77159069	0.89295613	0.29530039	2.80283408	0.64905615		
CAS	116_PB_M	0.17944272	1.30247187	1.07968063	0.63813057	0.04334852	0.47582564	0.96778096	1.78132183	0.58167668	0.40425017	2.93257793	0.84378402		
CAS	120_PB_M	0.15472722	3.5898724	1.71204662	0.66914498	0.03215111	0.39284638	0.88013664	1.03888275	0.46719582	0.22606249	0.94443886	0.65407415		
CAS	121_PB_M	0.45637259	1.1837479	3.41221628	1.12128631	0.08764772	1.44971338	0.94800576	2.59114859	0.63368292	0.25891337	4.14563625	1.06587683		
CAS	124_PB_M	0.35556091	0.17978927	0.30634485	0.58557066	0.00602646	0.62876026	1.15507076	2.65666275	1.22738823	0.34149918	1.75369874	0.97929912		
CAS	126_PB_M	0.489327	0.12157192	0.84087248	1.55713374	0.01722269	1.21065375	1.1407499	1.34944837	0.70005167	0.53086407	1.92691501	0.74462804		
CAS	128_PB_M	1.23459273	0.97319687	1.21850683	7.15721955	0.02211811	3.1156375	1.32306517	2.32843384	1.05764784	0.63941447	1.99666218	1.07775521		
CAS	19_PB_M	0.52828748	1.2484056	1.11382285	0.53029618	0.62470497	0.74321813	1.14495767	0.75326162	0.47807004	0.6457963	3.27116414	0.54636576		

CAS	1_PB_M	0.06428543	0.24307927	1.34497167	0.39274378	0.99742858	0.76740729	1.18224919	1.13604404	0.2872755	0.54441721	2.93704046	0.42689542		
CAS	20_PB_M	0.10921406	0.61821169	1.06107972	0.48094265	0.12524548	0.2384674	0.3657168	0.33064807	0.19337902	0.29758326	1.07210132	0.23045169		
CAS	23_PB_M	0.05516605	0.80542433	0.76129149	0.21865816	1.44434749	0.70813148	2.07123442	1.05016098	0.27081515	0.37111706	2.90273724	0.16850721		
CAS	28_PB_M	0.04515352	2.72827614	1.13586193	0.2508529	0.56291391	0.5538832	1.33955449	0.7615894	0.26389725	0.22175396	1.68974513	0.19566526		
CAS	29_PB_M	0.11447507	1.64482603	0.47798363	0.41572526	0.76919215	0.50911282	0.96902144	0.46693779	0.51312949	0.45589195	1.43595923	0.33840438		
CAS	30_PB_M	0.87056174	0.44834935	0.51972335	1.24854236	0.70670312	1.94519281	2.73935422	2.40862117	1.32896377	0.81627729	4.16080261	1.46266436		
CAS	31_PB_M	0.17966476	0.41051892	0.97761718	0.47074174	0.88828666	0.62430995	1.02077687	0.65943993	0.34427381	0.23085416	0.64839908	0.25996186		
CAS	32_PB_M	0.42566435	0.76699897	0.66861428	0.74190083	0.41763294	0.67564176	0.90353281	0.61440231	0.4949352	0.24796956	1.10431788	0.18171049		
CAS	35_PB_M	0.93062945	5.03061942	3.07398856	2.6563598	0.55516514	2.56901917	0.85633972	0.73586989	0.38951912	0.64551752	1.28601546	0.32125289		
CAS	37_PB_M	0.1493683	0.80492915	2.96973217	0.97400577	2.14613198	1.05180176	1.36817211	1.05180176	0.50100616	0.58087671	3.98626641	0.62547974		
CAS	3_PB_M	0.12634239	1.00171465	1.66651626	0.5775652	1.14209508	0.3178614	1.68556789	1.02878802	0.40810597	0.1734701	2.49275537	0.52141303		
CAS	46_PB_M	0.09030886	1.39175982	1.31750587	0.40237613	0.79873166	0.588011	0.83686207	1.03453812	0.5027193	0.42244476	2.58283329	0.2307893		
CAS	47_PB_M	0.0723669	0.61712884	1.09957484	0.17689687	2.01521715	0.56586896	1.86947825	2.19512931	0.68145498	0.69452122	7.6025449	0.54878233		
CAS	48_PB_M	0.04918099	4.81471816	8.52637707	0.35631123	1.54468444	0.55203148	1.47141481	1.31182753	0.42857716	0.3131524	3.15661635	0.36735185		
CAS	49_PB_M	0.06927294	0.54815975	0.46683935	0.09838765	1.25193261	0.67265024	1.09732345	1.1866755	0.32126579	0.73087967	4.45254302	0.43370881		
CAS	52_PB_M	0.25580579	2.74765511	4.33666048	2.08456638	0.42935246	0.35913126	0.60089281	0.78547424	0.35913126	0.4333651	1.50975573	0.64804133		
CAS	57_PB_M	0.76838088	1.95460024	4.98794697	4.98895139	0.0723182	1.88328646	1.06468461	1.27159502	0.75030133	0.19586179	0.84973885	0.60365609		
CAS	58_PB_M	0.19160932	0.06019141	0.20766036	0.26283582	0.13342429	0.52667483	1.39142473	2.10970887	0.62900022	0.42133986	2.07760679	0.75138942		
CAS	63_PB_M	0.11851554	2.03786471	1.80685984	0.64681364	0.12855923	0.46904033	1.1017928	1.57786371	0.66187917	0.59559082	1.76969819	1.05860493		
CAS	77_PB_M	0.29353519	1.50084944	2.28696081	0.54484956	0.33274023	1.15202509	2.1190828	3.22989234	0.36591373	0.45236587	2.34023945	0.1940147		
CAS	78_PB_M	0.12649713	0.42667684	1.30814099	0.7148092	0.26102583	1.29810154	2.05306856	3.60918409	0.93366932	0.46783861	2.41448894	0.48992541		
CAS	79_PB_M	0.29051942	2.21659278	1.44354977	1.84766328	0.11962564	1.19022488	1.05853614	1.542065	0.89668969	0.84843733	2.90318365	1.10578325		
CAS	83_PB_M	0.69078813	0.51528509	1.24994898	1.30198767	0.03163136	0.98465369	1.16423819	2.60805681	0.902004	0.8928207	2.08358842	1.31321171		
CAS	87_PB_M	0.17429852	1.27348748	1.61402448	1.56364919	0.04836028	0.78383961	1.02564103	1.63316709	0.6971941	0.21359125	4.16099945	1.17777442		
CAS	90_PB_M	0.36297823	0.54899201	0.82650445	0.44844402	0.09250415	1.27394299	2.58609421	3.37338495	0.97028807	1.0286059	4.06716605	1.3533759		
CAS	9_PB_M	0.08830731	1.22225344	5.27435475	0.68237466	2.69136595	0.65728736	1.70493317	0.38835146	0.36928511	0.61112672	2.90611327	0.191667		
ASCVD	101_PB_M	0.3563053	5.47542601	3.77341399	3.10609644	0.04026049	1.57720451	1.31551136	2.20828762	0.50023653	0.49520397	4.05121134	1.36382394		
ASCVD	111_PB_M	0.5036897	4.33716044	1.11093238	0.76609093	0.02714495	0.41220115	0.89578348	2.1112742	0.91790159	0.86059558	3.06838518	0.78820904		
ASCVD	112_PB_M	0.26107039	1.02219098	0.54423135	0.47896375	0.0180741	0.98403454	0.89165579	2.31549352	1.00010041	0.4940255	0.77015765	0.62656893		
ASCVD	114_PB_M	0.23615717	0.80494423	2.34046829	2.357552	0.02311325	0.9205105	0.55471812	1.13656919	0.47733896	0.17586172	1.22701236	0.3497136		
ASCVD	118_PB_M	0.22196109	0.85168781	0.85470085	0.67693111	0.0472044	0.67492241	0.84967911	0.97220967	0.73920074	0.45898742	1.65315817	0.62169193		

ASCVD	11_PB_M	0.20871799	0.54989163	1.83330657	0.37930481	2.81969977	1.57040218	2.53371598	1.70486473	0.75459581	0.58501244	3.56626796	0.50473629		
ASCVD	127_PB_M	0.22816233	0.45835277	1.0454905	1.68535908	0.03447786	1.41055023	2.36274768	2.33029793	0.78285031	0.88729795	5.77199992	0.92278987		
ASCVD	129_PB_M	0.05835186	0.60867028	0.61470668	1.00204232	0.0241456	0.30483817	0.28471684	0.38431743	0.23038925	0.19215872	0.56842762	0.4185237		
ASCVD	12_PB_M	0.06017028	0.25672654	1.46715204	0.53551551	0.5986943	1.15125806	1.28062417	0.88350031	0.32090817	0.32191101	0.4693282	0.12334908		
ASCVD	133_PB_M	0.51090706	0.21522463	1.38789713	1.58602448	0.03821746	1.00672828	0.70702296	1.38588569	0.65170822	0.47470105	1.74794581	0.70098863		
ASCVD	13_PB_M	0.3134009	0.657941	1.15717256	1.13708276	0.9311623	1.69758822	2.37160106	1.25059014	0.90605004	0.46708788	2.10842466	0.30436049		
ASCVD	14_PB_M	0.07725804	0.38328016	1.28930628	0.70535589	0.93211326	0.88495575	1.66656633	1.35352076	0.40134048	0.21572051	1.74482772	0.18963338		
ASCVD	15_PB_M	0.05417716	0.2708858	0.70931947	0.10133135	0.9942512	0.8277066	1.92529572	1.41763567	0.47555507	0.49160756	1.61628525	0.19062334		
ASCVD	21_PB_M	0.10936747	0.23880238	0.4234227	0.38930808	0.34114625	0.61305988	1.01039493	0.70637341	0.37526088	0.28596083	0.9552095	0.43546316		
ASCVD	22_PB_M	0.20884163	1.37654749	2.5824071	1.07834573	0.62250871	0.6807434	1.38658795	0.94179544	0.44178037	0.58636304	4.56439451	0.83938271		
ASCVD	25_PB_M	0.1033711	0.4445961	0.56803926	0.13046838	1.22138477	0.7868247	2.2229805	1.5375197	0.45262492	0.37936191	3.62200299	0.10036029		
ASCVD	34_PB_M	0.05433962	0.24754717	0.16603774	0.08050314	0.60075472	0.55748428	1.23572327	0.98113208	0.40955975	1.02641509	4.84528302	0.87748428		
ASCVD	36_PB_M	0.2246605	3.68182456	2.94766614	1.44524903	1.08920225	1.23362686	1.36401019	2.14229836	0.79032355	0.26879024	2.28772591	0.61280164		
ASCVD	4_PB_M	0.42457944	0.26498575	0.70462119	1.01076003	1.3299474	1.1432529	1.13020436	0.99369655	0.79194604	0.85317381	2.20520336	0.51290802		
ASCVD	54_PB_M	0.09630915	1.87100593	0.73736695	0.56481305	0.35514	0.74539271	0.73134763	0.92697559	0.35714644	0.42536542	0.47051034	0.33206593		
ASCVD	59_PB_M	0.34397344	0.23767261	0.80929029	0.98779546	0.04913906	0.80828745	1.03492885	1.3237462	0.40915792	0.55356659	1.31873201	0.6408135		
ASCVD	62_PB_M	0.3910284	1.34954931	0.54643713	0.63667445	0.07519777	0.43614707	1.01366594	1.7265408	0.76801989	0.2877568	2.5136108	2.47250268		
ASCVD	69_PB_M	0.28401108	1.81445948	1.11697644	0.56902573	0.12444302	1.12500502	1.18321224	1.7472201	0.82995464	0.26695034	2.61832122	0.39239693		
ASCVD	6_PB_M	0.15365457	2.53078113	1.38590395	1.15391568	0.72006749	0.88577339	0.97515416	1.31962159	0.40271557	0.73412738	2.2736859	0.31233053		
ASCVD	70_PB_M	0.19897698	0.32258389	0.74767106	0.72556251	0.06130099	1.00794903	1.94052799	3.65595072	1.04613653	0.40096876	4.86086686	1.52348029		
ASCVD	71_PB_M	0.19563974	1.74169534	1.54806216	1.58819339	0.07123293	1.15276956	1.2962387	1.95338758	0.87586408	0.25884643	0.86482799	0.43542384		
ASCVD	72_PB_M	0.20204457	0.39906316	0.52672316	0.35885528	0.04221827	0.53778032	0.87251088	1.4112964	0.34578772	0.23823166	1.29167798	0.17791985		
ASCVD	81_PB_M	0.18437544	1.36921434	6.3070506	1.90823544	0.14105225	1.05184678	2.21149777	2.38680557	0.71634393	0.64380277	3.43361477	0.42416427		
ASCVD	84_PB_M	0.31501435	0.94002689	0.57284456	0.92497843	0.00501615	0.51867012	0.41734385	0.64307069	0.29996589	0.47151829	0.2608399	0.18459439		
ASCVD	88_PB_M	0.35354607	0.74244674	1.4727719	0.74648727	0.038385	1.36973848	1.27882663	1.91015889	0.72123398	0.63638292	2.478863	0.79598372		
ASCVD	91_PB_M	0.29943429	1.21682861	1.1464917	1.31730991	0.03717808	0.81992745	1.56549874	2.53011927	1.07916922	0.47829101	2.43064278	0.57173863		
ASCVD	99_PB_M	1.57412334	2.41439198	1.56408429	2.31098975	0.02509763	0.73787032	0.6766321	1.56207648	0.65555009	0.66558914	0.8322374	0.73084298		

Table S6. Frequencies of peripheral myeloid cell clusters identified by myeloid cell panel.

Group	Sample	M01	M02	M03	M04	M05	M06	M07	M08	M09	M10	M11	M12	M13	M14	M15
NC	107_PB_M	0.40401226	0.041794372	8.219559766	13.15129563	0.013931457	5.906937866	0.794093062	0.027862914	2.201170242	0.208971858	0.027862914	0.891613263	19.57369741	47.01866815	1.518528838
NC	117_PB_M	0.203825651	0.047036689	8.748824083	8.905613045	0	3.041705864	0.219504547	0	6.522420822	2.038256507	0	0.360614613	56.78896206	12.15114456	0.972091565
NC	119_PB_M	0.509592326	0.209832134	2.083333333	8.767985612	0	8.168465228	2.622901679	0.01498801	3.447242206	0.029976019	0.029976019	3.552158273	16.05215827	53.19244604	1.318944844
NC	130_PB_M	0.209248797	0.167399037	8.872148985	7.21908349	0	8.955848504	0.62774639	0.188323917	0.083699519	0	0.418497594	0	25.2354049	25.69575225	22.32684662
NC	131_PB_M	0.18239098	0.18239098	6.831371249	8.63870005	0	4.775327475	1.492289836	0.016580998	0.679820925	0	0.033161996	0.082904991	24.75543028	50.12435749	2.205272757
NC	132_PB_M	0.565383914	0.091190954	5.051978844	5.653839139	0	8.170709466	2.006200985	0.091190954	4.01240197	0.054714572	4.103592924	0.072952763	25.38756155	39.5221594	5.216122561
NC	24_PB_M	0.654862964	0.097016735	11.83604172	10.93863691	0.048508368	9.919961193	1.430996847	0.048508368	49.96361872	0	0.048508368	0.145525103	2.498180936	10.89012855	1.479505215
NC	56_PB_M	0.618600683	0.149317406	7.764505119	11.86006826	0	7.444539249	1.514505119	0.021331058	10.21757679	0	0.063993174	0.063993174	11.90273038	47.14163823	1.237201365
NC	65_PB_M	0.197411713	0.087738539	9.146742707	11.87760474	0	7.600350954	0.998025883	0.164509761	17.28449221	0.109673174	0.581267822	0.054836587	9.278350515	33.4174161	9.201579294
NC	73_PB_M	0.466200466	0.093240093	9.603729604	7.272727273	0	9.184149184	5.454545455	0.979020979	41.77156177	0.13986014	1.375291375	0.20979021	14.87179487	7.855477855	0.722610723
NC	74_PB_M	0.39184953	0.039184953	14.77272727	8.659874608	0.019592476	9.326018809	4.036050157	0.176332288	42.12382445	0.470219436	0.411442006	0.156739812	6.406739812	12.61755486	0.39184953
NC	75_PB_M	0.19993848	0.076899416	13.99569363	7.505382959	0	2.614580129	2.876038142	0.261458013	39.7108582	0	1.184251	0.030759766	7.336204245	23.34666257	0.861273454
NC	86_PB_M	0.132375189	0.056732224	7.885779123	9.644478064	0	2.742057489	1.777609682	0.378214826	2.723146747	0	65.45007564	0	4.236006051	4.689863843	0.28366112
CAS	102_PB_M	0.525348043	0.236406619	3.204623063	6.015235093	0	4.202784345	1.208300499	0.236406619	12.1355398	0	0	1.182033097	6.724454951	63.51457841	0.814289467
CAS	103_PB_M	0.190839695	0.381679389	18.65458015	12.64312977	0	4.770992366	2.003816794	0	6.631679389	0.047709924	0.572519084	0.238549618	11.45038168	42.17557252	0.238549618
CAS	104_PB_M	0.308056872	0.118483412	5.497630332	4.502369668	0	6.184834123	2.061611374	0.142180095	12.34597156	0	0.047393365	0	16.27962085	51.84834123	0.663507109
CAS	10_PB_M	0.257834193	0.039666799	2.677508925	15.92621975	0.019833399	5.434351448	0.674335581	0.019833399	52.87584292	0	0	0.35700119	11.22570409	8.825862753	1.666005553
CAS	115_PB_M	0.469948058	0.371011625	20.72718279	11.05614643	0.024734108	4.872619342	0.44521395	0	17.70962157	0.098936433	0.049468217	0.915162008	15.92876577	24.85777888	2.473410834
CAS	116_PB_M	0.403992395	0.237642586	3.255703422	6.630228137	0	5.061787072	0.570342205	0.023764259	14.37737643	0.023764259	1.948669202	0.142585551	28.77851711	35.48003802	3.065589354
CAS	120_PB_M	0.526546731	0.394910048	8.336989908	5.528740676	0	7.6349276	11.10136025	0	0.702062308	0	0.833698991	0.219394471	12.68100044	49.53927161	2.501096972
CAS	121_PB_M	0.369287583	0.06154793	11.64794584	11.29404524	0	4.862286506	0.877058009	0.29235267	15.07924296	0.030773965	0.584705339	0.615479305	15.72549623	30.5277735	8.032004924
CAS	124_PB_M	0.221453287	0.138408304	6.615916955	11.07266436	0	3.98615917	0.415224913	0.01384083	0.747404844	0.01384083	0.041522491	0.110726644	9.757785467	49.96539792	16.89965398
CAS	126_PB_M	0.306056701	0.386597938	18.00902062	17.84793814	0.016108247	5.589561856	3.447164948	0	1.691365979	0	0.692654639	0.193298969	12.09729381	36.32409794	3.398840206
CAS	128_PB_M	0.197693575	0.098846787	10.24711697	14.13509061	0.016474465	4.10214168	1.004942339	0.016474465	0.757825371	0	0.049423394	0.065897858	11.8121911	54.72817133	2.767710049
CAS	19_PB_M	0.263929619	0.190615836	0.498533724	13.60703812	0	6.026392962	1.495601173	0.278592375	20.4398827	0	1.217008798	0.219941349	14.67741935	37.19941349	3.885630499
CAS	1_PB_M	1.10486139	0.200883889	4.41944556	15.91000402	0	6.126958618	4.017677782	0.020088389	13.43913218	0.1807955	0.662916834	0.020088389	6.64925673	46.28364805	0.964242668
CAS	20_PB_M	0.274245824	0.074794316	9.947643979	12.41585639	0	2.892046871	2.617801047	54.32560459	8.277237597	0	0.972326103	0.074794316	4.712041885	3.266018449	0.149588631
CAS	23_PB_M	0.218102508	0.109051254	12.12649945	12.25736096	0	5.299890949	1.352235551	0	55.68157034	0.021810251	0.021810251	0.283533261	2.246455834	5.845147219	4.53653217
CAS	28_PB_M	0.845219229	0.316957211	17.90808241	13.20655045	0.052826202	5.229793978	3.116745906	0.105652404	46.48705758	0.369783413	0.052826202	0.369783413	3.275224511	7.765451664	0.898045431

CAS	29_PB_M	0.357639579	0.079475462	2.880985496	4.768527717	0.019868865	4.152592887	3.238625075	0.079475462	44.70494735	0	0.019868865	0.675541427	16.94814226	21.63719452	0.437115041
CAS	30_PB_M	0.209918657	0.078719496	23.30097087	8.527945421	0.026239832	5.903962215	2.912621359	0.104959328	13.98583049	0	0	0.13119916	13.48727368	28.15533981	3.17501968
CAS	31_PB_M	0.115759881	0.181908384	5.870679676	14.61881925	0.049611378	2.34827187	1.174135935	0	22.32511989	0.016537126	0.099222755	3.241276666	21.64709773	22.12667438	6.184885067
CAS	32_PB_M	0.132831525	0.398494576	4.848350675	9.785255701	0	3.032986495	1.704671242	0.022138588	23.44476422	0.044277175	0.022138588	0.509187514	21.43015276	33.29643569	1.328315253
CAS	35_PB_M	0.156319786	0.156319786	8.575256811	14.98436802	0	2.367128182	5.337204109	0	17.17284502	0	0.200982582	0.714604734	7.034390353	35.66324252	7.637338097
CAS	37_PB_M	1.897983393	0.948991696	24.55516014	18.62396204	0	24.43653618	6.168446026	0	3.795966785	6.761565836	0	0.237247924	9.845788849	1.660735469	1.067615658
CAS	3_PB_M	0.667302193	0.905624404	8.007626311	13.53670162	0	7.292659676	3.527168732	0.285986654	20.44804576	0	0.095328885	0	17.44518589	27.16873213	0.61963775
CAS	46_PB_M	0.453785527	0.262717936	19.67996179	19.53666109	0.047766898	4.490088369	7.356102221	0	42.08263673	0.143300693	0.095533795	0.549319322	2.340577979	2.197277287	0.764270361
CAS	47_PB_M	0.286738351	0.023894863	12.21027479	6.594982079	0	6.833930705	2.508960573	0.047789725	61.57706093	0	0.262843489	0.023894863	3.632019116	3.94265233	2.054958184
CAS	48_PB_M	0.308546745	0.231410059	10.70657205	18.51280469	0.077136686	5.877815489	3.933970997	0.061709349	39.17000926	0	0.046282012	0.601666152	6.772601049	4.227090404	9.472385066
CAS	49_PB_M	0.371418465	0.035373187	2.882914751	8.790237	0	9.568447117	3.360452777	0.053059781	70.10965688	0.017686594	0.017686594	0.017686594	1.574106827	3.07746728	0.123806155
CAS	52_PB_M	0.177339901	0.236453202	5.379310345	9.793103448	0.019704433	4.354679803	3.802955665	0.216748768	15.25123153	0.0591133	0.118226601	0.216748768	11.84236453	47.44827586	1.083743842
CAS	57_PB_M	0.330169212	0.061906727	12.69087908	12.38134544	0	4.49855551	1.134956665	0.041271151	1.361947998	0	62.60833677	0	1.712752786	2.352455634	0.825423029
CAS	58_PB_M	0.214526509	0.36775973	10.97149862	11.33925835	0	4.596996629	2.696904689	0.153233221	13.26999694	0.122586577	0.030646644	0.183879865	5.577689243	47.90070487	2.574318112
CAS	63_PB_M	0.41625857	0.097943193	12.4387855	13.05093046	0	6.464250735	4.823702253	0.024485798	15.03428012	0.024485798	0.759059745	0.220372184	11.1410382	34.6963761	0.808031342
CAS	77_PB_M	0.258912567	0.199163513	13.44353714	10.05775742	0.019916351	3.385779725	1.633140809	0.119498108	44.23421629	0	0.039832703	0.099581757	9.639514041	7.926707827	8.942441745
CAS	78_PB_M	0.089498807	0.656324582	3.221957041	11.33651551	0	4.534606205	4.206443914	0.029832936	54.38544153	0.029832936	0.119331742	0.178997613	7.875894988	12.17183771	1.163484487
CAS	79_PB_M	0.338123415	0.056353903	16.37080868	17.69512539	0	8.340377571	2.648633418	0	7.100591716	0.028176951	0.817131586	0.394477318	10.76359538	33.30515638	2.141448295
CAS	83_PB_M	0.355029586	0.103550296	8.195266272	15.76923077	0	4.822485207	0.798816568	0.162721893	14.00887574	0	1.656804734	0.044378698	22.5443787	29.39349112	2.144970414
CAS	87_PB_M	0.237435694	0.224244823	15.02440311	10.53950666	0	4.695950402	1.015697138	1.23994196	9.734863474	0.118717847	1.939058172	0.171481335	22.88616278	31.48661127	0.68592534
CAS	90_PB_M	0.852333262	0.27700831	6.28595781	11.76219902	0	7.308757724	0.95887492	0.106541658	39.82527168	0	0.021308332	0.042616663	8.438099297	22.73598977	1.385041551
CAS	9_PB_M	0.337357955	0.088778409	2.095170455	10.63565341	0	5.202414773	2.041903409	0.142045455	61.02627841	0	0.905539773	0.088778409	6.747159091	9.747869318	0.941051136
ASCVD	101_PB_M	0.325027086	0.086673889	10.57421452	11.1375948	0.043336945	5.178764897	0.390032503	0	8.147345612	0	0	0.845070423	21.4084507	27.23726977	14.62621885
ASCVD	111_PB_M	0.242439709	0.063799923	1.888477734	6.903151716	0	4.529794564	1.939517673	0.051039939	8.625749649	0.012759985	0.242439709	0	23.15937221	50.03189996	2.309557229
ASCVD	112_PB_M	0.538448595	0.185091705	6.007067138	16.62460037	0	7.083964328	0.319703853	0	7.504627293	0.050479556	0.016826519	0.420662965	7.639239441	47.26569073	6.34359751
ASCVD	114_PB_M	0.135317997	0.210494662	6.525334536	7.006465193	0	2.601112615	0.64651932	0.210494662	11.35167644	0.060141332	0.571342655	0.210494662	21.22989024	47.22598106	2.014734626
ASCVD	118_PB_M	0.405797101	0.193236715	2.106280193	9.004830918	0.019323671	5.140096618	3.09178744	0.115942029	1.15942029	0.193236715	0.096618357	2.048309179	15.03381643	25.15942029	36.23188406
ASCVD	11_PB_M	0.584429138	0.041744938	10.83281152	16.88582759	0.041744938	5.656439157	1.168858276	0	54.56063452	0.041744938	0.062617408	0.333959507	1.85764976	7.43059904	0.500939261
ASCVD	127_PB_M	0.741656366	0.296662546	13.49814586	11.6934487	0.024721879	9.987639061	12.21260816	0.024721879	2.595797281	0	1.95302843	0.098887515	9.320148331	36.83559951	0.716934487
ASCVD	129_PB_M	0.236966825	0.28436019	10.04739336	6.492890995	0.047393365	8.341232227	3.6492891	0	3.033175355	0	0.09478673	0.09478673	18.38862559	48.05687204	1.232227488
ASCVD	12_PB_M	0.069483046	0.041689828	6.225680934	20.56698166	0	3.126737076	0.291828794	0.236242357	50.36131184	0.347415231	1.070038911	0.36131184	7.837687604	6.072818232	3.390772651

ASCVD	133_PB_M	0.22100884	0.14300572	7.592303692	11.57046282	0.02600104	2.938117525	0.767030681	0.325013001	4.706188248	0	0.15600624	0.14300572	25.96203848	32.77431097	12.67550702
ASCVD	13_PB_M	0.248851455	0.133996937	3.694486983	5.091883614	0	2.947932619	1.397396631	0.325421133	12.5957121	56.8338438	0.612557427	0.880551302	6.642419602	4.881316998	3.713629403
ASCVD	14_PB_M	0.236861584	0.162842339	8.171724648	6.854182087	0.029607698	6.202812731	1.03626943	0	67.74241303	0	0	0.370096225	2.635085122	6.395262768	0.162842339
ASCVD	15_PB_M	0.289203085	0.08033419	7.937017995	8.595758355	0	5.173521851	1.767352185	0.064267352	10.49164524	0.048200514	61.34318766	0.032133676	2.458226221	1.55848329	0.16066838
ASCVD	21_PB_M	0.143149284	0.163599182	5.214723926	19.14110429	0	4.110429448	0.470347648	0.429447853	10.83844581	1.00204499	0.18404908	1.104294479	9.63190184	14.19222904	33.37423313
ASCVD	22_PB_M	0.106112054	0.127334465	6.727504244	8.764855688	0	6.36672326	1.803904924	0.063667233	30.70882852	0	0.084889643	0	12.62733447	31.55772496	1.061120543
ASCVD	25_PB_M	0.340136054	0.094482237	16.02418745	18.76417234	0.018896447	6.027966742	1.058201058	0.302343159	49.6409675	0.170068027	0.699168556	0.510204082	1.133786848	4.894179894	0.321239607
ASCVD	34_PB_M	0.049095819	0.106374274	0.744619917	1.317404468	4.655920137	1.857458473	0.081826364	0	1.873823746	0.065461092	0.008182636	86.30226659	0.810081008	1.644709926	0.48277555
ASCVD	36_PB_M	0.502215657	0.147710487	5.819793205	8.892171344	0	5.849335303	4.933530281	0.029542097	36.04135894	0	0.059084195	0	5.76070901	30.69423929	1.270310192
ASCVD	4_PB_M	0.192122959	0.115273775	11.06628242	8.799231508	0.057636888	4.399615754	0.768491835	0.499519693	2.151777137	0	64.2074928	0.076849183	3.669548511	3.30451489	0.691642651
ASCVD	54_PB_M	0.147983722	0.240473548	3.810580836	6.992230855	0	5.105438402	3.163152053	0.018497965	13.33703293	0.018497965	0.055493896	0.332963374	21.03218646	35.88605253	9.859415464
ASCVD	59_PB_M	0.203547543	0.02907822	9.072404769	8.025588834	0	4.768828148	2.268101192	0.261703984	15.12067461	0.116312882	0.058156441	0.348938645	19.5114859	37.16196569	3.053213143
ASCVD	62_PB_M	0.435611217	0.108902804	5.200108903	10.97195753	0	5.662945821	4.737271985	0.163354206	11.95208277	0.054451402	0.136128505	0.027225701	10.75415192	48.7884563	1.007350939
ASCVD	69_PB_M	0.262008734	0.19650655	5.829694323	11.15720524	0	5.76419214	1.965065502	0	16.63755459	0	0.655021834	0.065502183	8.558951965	18.40611354	30.50218341
ASCVD	6_PB_M	0.224123879	0.203748981	13.97718011	6.907090465	0	6.173594132	1.691116544	0.32599837	16.15729421	0	3.300733496	0.020374898	19.98777506	28.66748166	2.363488183
ASCVD	70_PB_M	0.744416873	0.093052109	7.90942928	21.308933	0	5.303970223	1.674937965	0	13.12034739	0	0.03101737	0.093052109	3.815136476	11.44540943	34.46029777
ASCVD	71_PB_M	0.632083697	0.261551874	7.519616391	8.456843941	0.02179599	5.318221447	2.37576286	0	20.35745423	0.043591979	0	0.217959895	9.285091543	44.92153444	0.588491718
ASCVD	72_PB_M	0.222132472	0.262520194	6.542810985	8.52180937	0	2.786752827	1.171243942	0.403877221	29.01857835	0	1.51453958	0.18174475	20.47657512	21.40549273	7.491922456
ASCVD	81_PB_M	0.15176426	0.050588087	3.338813709	23.21993171	0	7.664095106	0.670292146	0.012647022	2.782344758	0	0.025294043	0.037941065	6.652333375	6.424686986	48.96926774
ASCVD	84_PB_M	0.175187132	0.063704412	6.497849976	10.75011945	0	4.108934544	1.974836757	0.047778309	12.1356904	0.031852206	1.082974996	0.573339704	14.55645803	46.64755534	1.353718745
ASCVD	88_PB_M	0.405896176	0.06408887	7.647938475	9.720145268	0	6.024353771	0.555436872	0.576799829	10.89510788	0.12817774	0.042725913	0.12817774	8.182012391	54.71053194	0.918607135
ASCVD	91_PB_M	0.244278735	0.179994857	7.379789149	11.63538185	0	4.577012085	1.542813062	0	22.07508357	0	0.565698123	0.012856776	20.72512214	29.63486758	1.427102083
ASCVD	99_PB_M	0.38616251	0.064360418	8.109412711	19.51729686	0	4.569589702	0.611423974	0	3.925985519	0	0.048270314	0.643604183	18.06918745	40.43443282	3.620273532

Table S7. Frequencies of peripheral T cell clusters identified by T cell panel.

Group	Sample	T01	T02	T03	T04	T05	T06	T07	T08	T09	T10	T11	T12	T13
NC	107_PB_T	3.13258232	2.09272097	20.4852686	6.29116118	4.38041595	1.01819757	1.40814558	3.73483536	1.50346621	3.48353553	2.57365685	9.67071057	5.45060659
NC	117_PB_T	4.77021524	3.5718441	6.13147179	0.95404305	5.99185573	1.75683537	0.61663758	3.42059337	1.38452589	2.66433973	2.32693426	8.50494474	11.5881326
NC	119_PB_T	1.85762712	0.02711864	11.6135593	5.37627119	10.5830509	1.43050848	2.37966102	4.56949153	2.03389831	3.82372881	5.60677966	9.17288136	5.43728814
NC	130_PB_T	7.22972973	0.5152027	19.8310811	3.10810811	2.86317568	0.88682432	0.38851351	1.61317568	1.51182432	3.29391892	4.11317568	8.30236487	5.32094595
NC	131_PB_T	4.38043976	0.07443885	11.1887311	1.30554283	2.13582226	0.81310124	0.24049473	0.93907467	1.49450298	3.35547412	3.88799817	9.20178653	6.37883646
NC	132_PB_T	5.71333111	1.12484687	9.90087983	2.48357278	4.788952	1.2083751	0.67936296	2.60608086	1.06916138	4.41029068	3.49148012	7.86279096	5.88595612
NC	24_PB_T	12.2180244	3.15079045	0.32278179	8.54271357	6.88112093	0.85830613	13.3294208	3.80735796	1.26545134	2.9380479	1.78263581	3.07743095	1.48552984
NC	56_PB_T	1.66505612	0.13379915	7.55221884	4.34847246	9.06860923	0.57236304	2.22998588	3.3524121	1.08525979	2.60908348	2.17795287	6.68995763	4.4376719
NC	65_PB_T	8.43704129	0.93815815	3.68243028	2.45069883	10.0963686	1.95928266	1.67209139	2.36135044	2.52728317	3.59946391	2.05501308	5.94166826	2.99317123
NC	73_PB_T	7.52275523	0.55596556	5.10209102	6.7896679	21.3431734	0.35424354	2.16482165	2.04674047	1.44157442	3.31611316	2.1303813	4.39852399	3.33579336
NC	74_PB_T	4.83557284	0.6718529	3.05869873	3.36810467	11.4303395	1.37022631	3.4476662	4.45544555	2.88189533	5.05657709	5.54278642	9.24681754	3.81895332
NC	75_PB_T	0.21831413	0.01212856	5.28805337	5.63978169	19.9636143	0.92177077	4.88781079	4.43905397	2.94724075	3.33535476	4.00242571	13.8993329	5.37295331
NC	86_PB_T	14.5506524	0.64506671	15.3056737	4.24424571	12.4908371	1.10687583	0.72570004	2.43366075	2.44099106	2.7488638	2.66823047	5.53437912	5.92288521
CAS	102_PB_T	2.97434626	0.05576899	7.01449994	2.12541827	8.39013509	0.79935556	0.99764531	3.62498451	1.75362499	2.93097038	2.44763911	6.13458917	7.29954146
CAS	103_PB_T	0.46521932	0	12.9818343	3.98759415	3.56668144	1.66149756	0.77536553	1.59503766	1.26273815	4.23128046	3.02392557	8.20779796	5.51617191
CAS	104_PB_T	1.88325179	0.67019637	20.7291737	6.42718317	6.58803029	0.36190604	1.54145165	2.89524831	1.06561222	5.4621004	2.17143623	11.9361973	5.80390054
CAS	10_PB_T	3.82570504	0.26583449	0.97087379	7.1197411	4.35159501	1.68747111	20.1745261	2.51386963	2.30582524	3.79103098	3.16111882	6.6285252	7.2295423
CAS	115_PB_T	2.66488322	0.01028912	10.4743286	1.78001852	12.737936	0.57619097	1.53307954	6.7496656	1.94464451	4.95935796	3.62177179	16.7712728	7.37730219
CAS	116_PB_T	0.79992559	0.01860292	6.00874337	0.91154311	6.40870617	0.59529346	1.00455772	3.4694447	1.79518184	5.17161194	3.06018045	11.8221561	6.61333829
CAS	120_PB_T	6.1150536	0.45296693	6.93794353	3.26136192	6.34153707	0.59640646	1.20791182	2.43092254	0.74739544	1.87981277	2.30258191	6.87754794	4.93733957
CAS	121_PB_T	10.3896901	1.7060448	3.68211108	1.46670758	11.6722921	1.41761277	1.86560295	6.29027309	2.52838294	2.5590672	2.92114145	5.97729365	4.67628107
CAS	124_PB_T	1.18983957	0.04010695	17.921123	3.03475936	4.03074866	1.12299465	0.80882353	3.15508021	1.35695187	3.80347594	5.90909091	24.552139	8.9973262
CAS	126_PB_T	3.78279439	0.01220256	31.3605857	6.63209274	2.61744966	1.30567419	0.61622941	1.19585113	1.25686394	2.40390482	3.42281879	8.59060403	7.26052471
CAS	128_PB_T	16.5890114	1.16252249	8.39599576	2.3481109	2.05748028	2.4080823	0.81653365	1.86833972	1.25478618	2.79097661	4.68238225	8.3221848	3.150805
CAS	19_PB_T	4.35456251	0.53004974	2.07942592	7.01296583	9.51643154	1.27211938	7.7713447	4.82752997	1.83478757	3.84082199	3.49017369	4.40349017	1.90817908
CAS	1_PB_T	0.81542594	0	2.17766692	15.7808903	4.89255564	0.69071374	9.29585572	1.40061397	1.07444359	3.56868764	2.30237913	6.8303914	2.64773599
CAS	20_PB_T	4.86610999	0.69104521	0.67664843	20.6017852	1.58364526	0.82061618	12.4532105	0.33112583	1.52605816	1.10855168	1.75640657	1.48286784	2.26029369
CAS	23_PB_T	2.73306823	0.16411033	2.55002209	10.338951	14.2397273	1.18033201	11.5003472	5.70599003	1.90620463	2.98554567	1.57167203	7.42283658	5.10004418
CAS	28_PB_T	11.3928546	13.0833156	0.85233326	2.5214859	6.05866894	0.51850274	6.02315505	3.76447191	1.41345266	1.05121102	1.44896655	4.11250799	5.27736345

CAS	29_PB_T	2.73088381	0.41377028	1.04270109	12.9675604	8.51539225	0.77788812	11.5524661	1.36544191	1.8123138	4.04667329	2.09367759	2.58192651	5.02317114
CAS	30_PB_T	0.37759597	0.07403843	1.37711472	8.56624588	10.2506201	0.92548032	13.6119646	2.81716211	2.49509495	3.55014252	4.27201718	6.37470847	6.83744864
CAS	31_PB_T	2.52393386	0.11604294	1.63427135	5.00918673	12.2522	0.8219708	8.92563582	4.64171744	3.34590465	2.97843535	2.69799826	5.81181704	5.40566676
CAS	32_PB_T	9.19455294	2.3540589	1.31953975	4.35975932	8.92008867	1.5306661	8.44505437	5.20426475	2.77631162	5.20426475	4.27530877	8.56117386	2.058482
CAS	35_PB_T	7.09614295	0.78273856	0.04119677	0.42741645	0.15448787	9.14568206	3.84159844	0.71579381	0.79303775	0.46346362	1.24620217	0.35017251	1.02991915
CAS	37_PB_T	4.76210299	0.258086	2.11880281	23.1986013	3.00961579	0.68684178	9.16205303	0.74511926	1.33205678	4.23344295	1.19052575	3.28851517	2.09798943
CAS	3_PB_T	1.29899216	0.04479283	3.85218365	22.1201941	3.85218365	0.44792833	10.1381112	2.82194849	0.90332214	3.47891004	2.02314296	7.2489735	4.02388951
CAS	46_PB_T	5.99608829	0.07264599	0.87175189	26.677843	10.6398435	0.78792959	13.3277452	0.77116513	1.75468008	2.46996368	1.46968427	0.92204526	1.75468008
CAS	47_PB_T	0.75088985	0.32668585	1.5554147	16.8413867	11.0146765	0.46321127	18.8356331	2.41357453	1.76020284	3.29123799	2.35506363	3.97874104	5.34887123
CAS	48_PB_T	10.0876199	0.0566508	0.83087847	11.0280233	4.01465368	0.34368155	11.099781	0.98950072	1.2463177	2.37933379	1.36339603	1.48425108	1.39360979
CAS	49_PB_T	1.91884457	0.13755158	1.77441541	14.7592847	9.869326	0.82530949	14.0784044	1.91196699	2.00137552	4.47042641	1.76066025	3.39752407	4.28473177
CAS	52_PB_T	17.6607281	2.24632068	3.47862827	4.40109851	3.0913316	0.49292303	2.60545032	1.85902401	0.77459334	1.50693613	1.39426801	3.85184142	2.55615802
CAS	57_PB_T	7.5529132	0.0332937	2.40665874	1.64565993	3.53864447	1.61712247	1.41736029	1.41260404	1.14625446	1.87871582	2.79191439	2.90606421	2.47324614
CAS	58_PB_T	0.44105486	0.04594321	1.63557843	4.05219149	14.637508	0.69833686	10.5577506	8.29734448	4.20839842	6.5423137	3.30791142	17.5043646	4.6126987
CAS	63_PB_T	1.3185288	0.12491326	11.3046496	5.59333796	7.83483692	0.40943789	1.33240805	1.77654407	1.2907703	2.35947259	1.83206107	7.41151978	3.18528799
CAS	77_PB_T	4.48583549	0.955678	4.10551465	3.55453703	13.267346	0.80940075	4.21766054	6.20215515	2.18928275	4.08113511	2.31605637	16.4903213	3.91535424
CAS	78_PB_T	1.40941904	0.03437607	4.37722012	3.93033116	14.8447347	0.73335625	4.06783545	5.19078721	1.40941904	5.0017188	4.10794087	10.0836485	3.46625415
CAS	79_PB_T	8.52995716	2.60246819	4.59748066	2.5129484	6.45181917	1.40034529	2.01419528	2.42342861	2.23799476	2.48737132	3.42732911	9.58501183	6.18965407
CAS	83_PB_T	10.0356436	1.11683168	2.70891089	0.72871287	6.58217822	1.98019802	1.74257426	7.55643564	3.27920792	4.34059406	4.16633663	16.7287129	4.35643564
CAS	87_PB_T	4.09129726	1.01700179	6.72308051	1.73899542	6.23398804	1.59149134	1.20332272	3.27614316	2.36006521	6.43583573	3.13640245	14.6650105	6.42030898
CAS	90_PB_T	3.60877068	0.61415085	10.9329002	5.38016445	9.61831286	1.3805705	2.30941021	4.58836666	2.71038473	7.85199472	4.14678713	13.5620749	4.72540859
CAS	9_PB_T	0.64645361	0.03186743	0.51443139	14.7182009	2.92269872	0.34598926	11.6725849	0.20030957	1.95757079	2.41281981	1.44769189	1.52053173	1.87562597
ASCVD	101_PB_T	8.45515769	0.10676741	8.72618265	2.47207622	7.39980289	0.82128778	0.91573587	1.53580815	1.41672142	2.88272011	1.87664258	8.93561104	2.5131406
ASCVD	111_PB_T	2.13390657	6.52230902	8.34687779	2.09196246	4.31500026	0.4194411	1.50474493	4.90746081	0.45089918	1.81932575	3.09862109	10.1557175	2.19157972
ASCVD	112_PB_T	0.31174229	0.04329754	12.1925875	2.85763769	6.05299619	1.51541392	1.30758573	3.31659162	2.62383097	8.25251126	4.52026325	14.1669553	10.6598545
ASCVD	114_PB_T	18.4007624	1.16087672	5.89967946	1.03959109	5.90834272	1.07424413	0.5717751	3.08412025	0.97894828	2.91951832	1.97522308	4.98137399	3.7511912
ASCVD	118_PB_T	3.97392957	0.06336562	13.4606681	5.1054585	7.75776229	1.43025256	1.05911107	2.31737123	2.02769983	3.97392957	3.59373586	6.98832262	7.30515072
ASCVD	11_PB_T	4.81017241	0.11312729	1.0271958	9.14973528	6.01384678	1.65618354	15.9826237	1.52043079	1.61093262	2.52952622	3.50242092	3.98208064	6.07267297
ASCVD	127_PB_T	5.99946193	0.05380683	18.9238633	5.32687651	2.45359161	1.26446059	0.89857412	1.51197202	1.61958569	3.61043853	3.27145548	9.92198009	7.97417272
ASCVD	129_PB_T	2.84216892	0.26204394	13.5254989	4.55553316	4.39427535	0.62487402	0.94738964	1.12880468	1.43116307	4.25317476	2.5196533	4.61600484	4.77726265
ASCVD	12_PB_T	10.088818	0.10248235	0.04554771	13.5048964	0.25051241	1.53723525	18.9706217	0.11386928	4.28148486	7.31040765	3.06308358	0.86540651	2.04964701

ASCVD	133_PB_T	9.07970419	0.18077239	9.0221857	1.88989318	3.35250616	1.26540674	0.64913722	4.65899754	1.83237469	5.39852095	3.00739523	15.4889072	13.1142153
ASCVD	13_PB_T	12.5	0.09942554	0.49712771	19.2443659	1.73994697	1.45824127	18.4544852	0.54131684	1.40300486	3.38599205	1.93327441	1.17101193	4.3857711
ASCVD	14_PB_T	0.50485131	0	0.9544845	18.7978228	3.84949121	0.82038337	17.0939497	0.4575215	1.77486787	3.61284216	2.06673503	2.73724067	3.03699614
ASCVD	15_PB_T	2.35544287	0.08178621	0.08178621	24.6585426	0.29443036	1.3576511	17.8948229	0.01635724	2.21640631	3.59041466	2.35544287	0.14721518	3.15694774
ASCVD	21_PB_T	5.91103887	0.26599675	1.063987	15.3243683	2.77818827	1.77331166	16.5952416	0.85710064	3.02940742	4.6697207	2.80774346	3.42840254	2.46785873
ASCVD	22_PB_T	6.5201367	0.31495008	1.57475039	14.7624472	3.71909134	0.87783958	14.943376	2.28506333	1.36701736	2.59331234	2.13093882	5.56858541	4.06754674
ASCVD	25_PB_T	1.24973964	0.04165799	1.45802958	12.0738735	12.1224745	2.0828994	15.1357356	7.64424078	1.77740749	4.09636881	3.13823509	5.77657433	1.91626744
ASCVD	34_PB_T	0.37364958	0.02436845	1.7220372	32.003899	8.44772967	0.86914142	17.0416701	1.16968565	2.44496792	5.46665584	1.68142312	1.47835269	4.55690033
ASCVD	36_PB_T	9.51205279	3.29487813	1.61601652	11.9405665	2.17713337	0.92023163	8.53346501	1.20752346	1.11774476	3.77519415	2.31180141	4.74929299	3.36670108
ASCVD	4_PB_T	6.69601647	0.09941064	5.46758503	16.6938863	8.27238515	1.83199602	8.46410566	2.64148264	1.64027551	4.4663779	4.49478094	11.1623944	2.74089328
ASCVD	54_PB_T	3.370341	2.94739625	1.32170235	2.15437484	2.51123447	1.58604282	5.617235	3.26460481	2.33941316	3.40999207	3.99154111	9.41052075	4.59952419
ASCVD	59_PB_T	2.79582595	0.35440047	2.96318173	6.40874188	15.3868872	2.06733609	5.69009648	5.39476275	3.79996062	4.84347313	2.35282536	7.41287655	3.62276039
ASCVD	62_PB_T	2.08662662	1.35946886	6.14922542	2.93234271	6.08599431	0.82200443	2.4502055	4.93202656	1.15396775	2.97186216	4.07840658	10.5042681	8.37021815
ASCVD	69_PB_T	2.71523998	0.40821376	2.30702623	1.65141019	7.65710045	0.85353785	2.35650668	4.50890648	1.66996536	3.15437902	3.30900544	9.06110836	7.86739238
ASCVD	6_PB_T	4.87090401	4.65971796	4.4076572	10.4707405	5.09571497	0.76299475	5.40908781	1.90748689	1.22624157	3.7672866	1.74398801	10.7841134	3.57653791
ASCVD	70_PB_T	0.3827228	0.09719944	7.14415892	4.13705121	21.8759492	0.80797035	2.6790596	4.11882632	3.5477796	7.62408116	3.8090031	15.3332118	3.46273009
ASCVD	71_PB_T	16.5857084	6.22695617	4.64680751	2.68625271	10.6806344	0.87200796	2.27073214	5.71779716	1.4455434	2.20635571	3.1661497	7.47351788	4.30736818
ASCVD	72_PB_T	0.18573551	0.07429421	4.3833581	1.77067855	10.4754829	1.28776622	3.07082714	6.83506687	2.29073799	4.29668153	2.61267954	10.277365	5.80733036
ASCVD	81_PB_T	12.136665	0.56603774	0.44875064	0.24477308	1.06578276	1.46353901	1.94288628	5.82355941	1.84599694	2.48342682	2.41713412	9.49515553	3.97246303
ASCVD	84_PB_T	8.93880012	0.13566476	5.98432318	1.91438046	8.00422068	2.32137474	0.73861923	3.66294845	1.55260778	2.90925535	2.92432921	8.23032861	5.38136871
ASCVD	88_PB_T	5.60497577	1.78636002	13.8424821	4.62862515	6.0967672	2.63976278	1.16438852	2.49511825	2.66145946	6.87061546	4.91791423	11.3618283	6.6102553
ASCVD	91_PB_T	6.22954282	0.32731496	8.4890719	2.73994298	9.99366487	0.67574702	1.60489917	4.14422975	2.6765917	3.55823039	3.40513145	14.5549572	6.96336184
ASCVD	99_PB_T	6.08056196	0.14817254	4.69213039	0.76830205	5.6250686	2.05246405	1.15794095	7.61716606	1.2457469	1.90429152	4.21468555	7.81472945	6.09702557
**Group**	**Sample**	**T14**	**T15**	**T16**	**T17**	**T18**	**T19**	**T20**	**T21**	**T22**	**T23**	**T24**	**T25**	**T26**
NC	107_PB_T	0.05199307	9.39774697	1.21750433	2.48266898	1.49480069	3.76516465	4.29809359	1.62478336	0.48093588	3.01559792	0.30329289	3.59185442	3.05025997
NC	117_PB_T	0.18615474	2.52472368	0.20942408	2.87376382	5.99185573	3.69982548	6.56195463	7.44618965	1.80337406	4.52588714	1.86154741	5.10762071	3.52530541
NC	119_PB_T	0.11525424	1.59322034	0.09491525	0.33220339	0.87457627	0.90847458	1.4779661	4.35932203	0.3661017	14.7457627	1.29491525	6.19661017	3.72881356
NC	130_PB_T	0.11824324	2.08614865	0.43918919	1.5625	3.64864865	1.25844595	5.41385135	2.74493243	1.84966216	11.0641892	6.08108108	2.06081081	2.69425676
NC	131_PB_T	0.18323408	1.40288594	0.0171782	4.51213926	7.2091159	3.00618415	8.74370133	5.93792946	5.79477783	5.92075126	0.79019698	4.98740266	6.09825928
NC	132_PB_T	0.11693953	0.80187103	0.9689275	0.99120169	2.6450607	0.94108475	8.56442811	12.3733155	2.24969373	11.404388	0.9800646	3.49704867	3.24089542
NC	24_PB_T	0.18706672	7.04251183	2.35117192	2.14576532	1.2140997	1.98070645	3.31218135	6.81509739	0.68224333	4.93342626	2.33283204	5.68169314	1.66159264

NC	56_PB_T	0.11893258	0.71359548	0.87712778	2.31175203	0.55749647	4.7052702	27.6592582	4.17007359	1.00349365	3.07738051	4.31130603	3.06994722	1.50152382
NC	65_PB_T	0.09573042	0.33186547	1.21896739	4.8184313	0.8360457	8.27110856	8.26472653	9.40710958	0.65734891	2.15712553	1.74867573	9.94958198	3.5292616
NC	73_PB_T	0.20172202	1.76629766	0.34440344	1.13653137	1.48585486	2.17466175	3.72447725	12.4477245	1.19065191	5.67281673	0.51168512	5.46125461	3.3800738
NC	74_PB_T	1.32602546	2.21004243	0.44200849	0.39780764	3.35042433	10.0159123	2.18352192	5.67538897	1.22878359	1.98019802	1.30834512	6.3384017	4.35820368
NC	75_PB_T	0.23044269	1.58884172	0.02425713	0.14554275	2.92298363	0.06064281	0.73984233	1.29775622	0.0970285	11.6798059	1.10369921	4.76652517	4.41479685
NC	86_PB_T	0.10262425	3.18135171	1.08488491	0.38850608	2.16976983	1.03357279	4.30288814	2.41900015	0.43981821	7.30831256	1.99384255	1.81791526	2.93945169
CAS	102_PB_T	0.11773454	0.94187632	0.48952782	11.0298674	1.55533523	4.76515058	6.1036064	11.5689677	1.62349734	5.94249597	3.40810509	3.38951543	2.51580122
CAS	103_PB_T	0.12184316	2.86885246	5.53832521	1.50642446	4.46389012	2.15994683	9.66991582	4.92910944	1.80549402	6.62383695	8.48471422	2.33717324	2.21533008
CAS	104_PB_T	0.06701964	4.24234301	2.11782052	2.26526372	1.95027143	1.03210241	1.66878896	6.76898331	1.13263186	5.82400643	0.77742779	3.29066417	1.32698881
CAS	10_PB_T	0.54900601	0.51433195	3.85460009	4.58853444	0.71659732	1.14424411	2.9877485	5.53629219	0.73971336	5.87725381	1.77415626	5.38603791	2.30582524
CAS	115_PB_T	0.10289124	2.24302912	0.11318037	0.21607161	1.08035806	1.61539253	0.91573207	4.49634736	1.47134479	5.27832082	0.87457557	6.86284597	3.52916967
CAS	116_PB_T	0.15812483	9.10612966	1.73007162	0.60459492	3.15319505	7.58069017	2.65091619	8.4178216	2.00911543	6.86447772	4.30657613	3.80429728	1.93470375
CAS	120_PB_T	0.02264835	5.40540541	27.5705874	6.41703156	1.07202174	0.41521969	2.26483467	0.59640646	0.46806583	6.05465801	1.11731844	3.32930696	1.17771403
CAS	121_PB_T	0.06750537	5.89751458	2.19085609	1.39920221	2.01902424	3.52255293	2.54065664	14.8941393	1.27032832	2.41178276	0.97575944	3.57164775	2.08652961
CAS	124_PB_T	0.22058824	1.21657754	0.28074866	0.38770054	1.5040107	0.80882353	1.67780749	0.39438503	1.55748663	2.76737968	5.70855615	4.34491979	3.20855615
CAS	126_PB_T	0.28676022	0.37827944	0.04881025	0.21354484	5.23489933	0.21354484	3.99633923	2.63575351	0.51250763	7.84014643	0.7504576	3.12385601	4.30750458
CAS	128_PB_T	0.27217788	1.23172026	0.10610324	3.07699405	4.38713844	2.13129123	15.5233658	3.08160723	3.20616321	0.41979979	0.65045901	3.92581999	6.14014855
CAS	19_PB_T	0.08970073	2.12019897	5.25156976	2.96827856	4.96615836	4.26486178	3.76743048	4.21593411	1.08456332	3.75927587	1.1416456	9.68767838	3.84082199
CAS	1_PB_T	0.13430545	2.61895626	3.50153492	0.08633922	1.08403684	0.52762855	0.88257867	12.1450499	2.51343055	11.3679969	4.19224866	6.8495779	2.61895626
CAS	20_PB_T	0.08638065	1.98675497	1.33890009	0.4031097	1.77080334	8.75323927	6.72329398	15.04463	0.76302908	6.93924561	2.00115174	3.0665131	0.96458393
CAS	23_PB_T	0.09467904	2.37328789	3.12440826	0.75112037	0.66275327	3.33901408	1.74840624	3.69248248	0.59963391	6.86107429	0.99097393	6.791643	1.57167203
CAS	28_PB_T	0.04261666	1.12934157	1.64784431	8.6866965	1.25719156	1.54840543	5.83848285	6.56296612	1.38504155	6.27175226	1.20036934	5.21343845	1.69756375
CAS	29_PB_T	0.09930487	1.33234029	0.38894406	1.44819596	1.38199272	13.3316783	2.26746111	5.06454816	1.2082092	5.02317114	3.26878517	7.51406819	2.74743462
CAS	30_PB_T	0.85514382	3.16144079	0.85514382	2.76903713	1.31048014	0.57009588	1.91019139	1.89908563	0.18509607	5.74167993	5.27893977	9.125236	4.80879577
CAS	31_PB_T	0.25142636	1.40218548	0.26109661	2.05976211	2.1661348	2.32085872	5.85049802	7.9682816	0.57054444	9.91200077	2.03075138	6.07291365	2.96876511
CAS	32_PB_T	0.36947113	1.1189697	0.43280904	1.6467856	1.82624301	7.15718357	1.98458778	3.84249974	1.2350892	1.72067983	0.98173757	8.47672332	5.00369471
CAS	35_PB_T	0.20598383	1.28224934	5.18564293	10.6699624	3.2751429	16.8649261	15.0162212	11.936763	4.03213348	0.3604717	0.38621968	2.48725475	2.20917658
CAS	37_PB_T	0.07492819	1.42363568	0.41626774	0.39545436	1.74416185	3.12617075	9.38267494	4.4832036	0.53698539	14.1614286	1.09062149	4.64138534	2.43932898
CAS	3_PB_T	0.268757	6.00970511	3.15789474	1.8514371	2.81448302	1.38857783	4.29264651	2.09779769	1.79171333	6.5248227	2.13512505	4.06868234	1.34378499
CAS	46_PB_T	0.04470523	0.88851635	1.03380833	2.60966751	0.50293378	4.71081308	1.05616094	6.86225203	0.65940207	8.29281922	2.74378318	2.47555183	0.60352054
CAS	47_PB_T	0.34618948	0.62411624	0.1170218	0.31693403	2.15027549	2.56960359	1.46764835	3.28148618	0.18528451	7.67467941	3.04744259	6.96767273	2.31605637

CAS	48_PB_T	0.13596193	0.80444142	20.3414155	3.45569907	0.5023038	0.49852708	4.13550872	17.2105144	0.94040335	1.2463177	0.59294509	3.04781328	0.77045094
CAS	49_PB_T	0.25447043	2.63411279	0.72902338	3.87207703	1.14167813	0.28885832	0.83906465	2.14580468	0.50206327	14.1059147	5.22008253	4.93810179	2.1389271
CAS	52_PB_T	0.0492923	3.99267657	7.79522569	2.52094923	0.73938455	7.65439054	12.8652912	5.4996127	1.16189001	5.27427646	2.87303711	2.21815365	1.43651856
CAS	57_PB_T	0.19976219	0.47562426	0.51843044	7.60047562	3.92865636	4.31866825	20.3709869	18.9488704	4.71819263	0.83709869	1.45541023	1.98335315	3.82401903
CAS	58_PB_T	0.29403657	0.48699807	0.22971607	0.28484793	0.90967564	0.41348893	0.900487	1.4793715	0.71671414	5.43048792	0.79022328	9.90535698	1.61720114
CAS	63_PB_T	0.12491326	2.37335184	0.47883414	2.88688411	3.33102013	8.09854268	8.51492019	5.03816794	2.56072172	10.9576683	3.18528799	3.61554476	3.06037474
CAS	77_PB_T	0.14627724	7.11882588	1.12145887	1.23848067	2.08201278	2.85240626	7.24559949	2.18928275	1.14583841	1.17509386	1.17509386	4.03725194	1.88210054
CAS	78_PB_T	0.83075513	4.938696	1.43233643	1.15732783	4.04491807	0.49845308	2.79019136	6.27363355	0.56720523	4.96161338	0.18333906	8.03827203	5.62621749
CAS	79_PB_T	0.01278854	3.90050515	0.82486093	5.28166763	1.17015154	4.16906452	11.0365113	2.91578745	2.05895518	2.86463329	4.25219004	3.51045463	3.54242599
CAS	83_PB_T	0.22970297	0.6970297	0.07920792	3.1049505	1.31485149	0.92673267	3.92871287	2.04356436	3.8019802	1.56831683	0.21386139	8.98217822	7.78613861
CAS	87_PB_T	0.07763372	0.72199363	0.17079419	4.76671066	1.42069715	4.60367984	14.8979117	4.17669436	1.37411692	0.88502446	1.7157053	2.85692105	3.43917398
CAS	90_PB_T	0.21317633	2.05562887	0.11673942	0.78164653	1.87798193	1.05573038	2.18251954	1.81707441	0.7562684	3.9894427	1.34504111	8.61333875	3.76611512
CAS	9_PB_T	0.22307202	0.89684057	1.05617773	3.85595921	2.56305199	3.42347264	8.3265046	19.2797961	0.59182373	12.1642539	2.36729491	3.61467723	1.37029955
ASCVD	101_PB_T	0.10266097	3.45351511	23.4888305	1.1087385	1.62204336	3.37138633	4.86613009	7.93363995	0.21353482	2.15588042	0.39421813	1.37565703	1.85611038
ASCVD	111_PB_T	0.11010329	3.30309862	0.28312274	1.21113616	1.58339013	20.1489016	2.89414355	0.78645205	1.23210822	2.11293452	7.51323861	6.46463587	4.39888848
ASCVD	112_PB_T	0.3204018	1.29026671	3.54173883	0.23380672	2.13889851	5.36023554	4.34707309	1.32490475	0.64946311	2.56321441	1.65396606	5.12642882	3.62833391
ASCVD	114_PB_T	0.21658148	0.97028502	0.0433163	1.7586416	1.96655982	3.62124231	12.9948887	8.0308412	4.19301741	3.59525253	3.01481417	4.5568743	3.29203847
ASCVD	118_PB_T	0.09957455	1.40309586	0.65176066	5.49470445	1.30352132	2.93292297	6.57191998	3.71141486	0.62460397	5.23218974	5.44944329	3.89245949	3.57563139
ASCVD	11_PB_T	0.63351283	1.77383592	0.28960587	0.27603059	4.01828137	1.87791303	0.85524232	9.70632155	0.87786778	6.69261053	4.71967057	6.81931309	3.48884565
ASCVD	127_PB_T	0.23675007	0.70486952	0.27441485	0.19908528	2.10384719	1.19989239	7.5921442	3.01318267	0.83400592	3.61043853	1.90476191	11.7298897	3.76647834
ASCVD	129_PB_T	0.06047168	1.24974804	1.4513203	2.70106833	1.22959081	6.08748236	17.0126991	6.93408587	5.5835517	7.31707317	0.52408789	1.93509373	2.03587986
ASCVD	12_PB_T	0.70598952	1.66249146	0.53518561	4.03097244	3.07447051	1.7194261	8.99567297	7.70895013	1.05898429	0.26189934	1.37781827	4.24732407	2.43680255
ASCVD	133_PB_T	0.05751849	2.94165982	0.13968776	0.5505341	2.13640099	0.60805259	8.79211175	6.32703369	0.53410025	1.56943303	0.36154478	3.83730485	3.20460148
ASCVD	13_PB_T	2.34202386	2.47459125	0.54684048	1.68471056	2.2536456	1.70680513	0.92797172	6.92664605	1.27596111	5.19222271	1.39195758	4.53490941	1.92775077
ASCVD	14_PB_T	0.31553207	1.15958034	0.20509584	0.71783545	2.28760748	1.64076674	3.89682101	11.3354895	1.60921354	9.48962688	0.9308196	7.52543977	3.17898556
ASCVD	15_PB_T	0.40893105	2.870696	2.03647665	0.67064693	2.78073117	0.59703934	2.48630081	4.2283471	3.41048499	6.71464791	2.39633598	10.5913143	2.60080151
ASCVD	21_PB_T	0.31032954	0.29555194	0.45810551	1.37431654	3.42840254	1.46298212	1.71420127	6.51692035	0.45810551	5.43815576	3.91606325	9.56110536	4.09339441
ASCVD	22_PB_T	0.44897139	1.4742344	12.0485157	0.82423105	1.28660457	1.46753334	5.23353213	6.86859211	0.58969376	3.38403806	0.66340548	3.62527642	1.36031629
ASCVD	25_PB_T	0.13885996	0.88176074	0.75678678	0.40963688	1.87460946	1.59688954	3.84642089	4.1380268	1.36082761	6.42921614	0.57626883	7.15128793	2.32590433
ASCVD	34_PB_T	0.11371944	0.56047437	0.95849241	1.15344001	0.22743888	0.12996507	0.82852733	1.34838762	0.14621071	12.4847697	0.97473804	2.61554707	1.17780846
ASCVD	36_PB_T	0.29178076	0.95165417	0.11222337	0.13466804	1.28832428	14.2658347	4.21062082	7.26309647	2.19060017	3.76621628	0.99654352	6.27553082	3.7303048

ASCVD	4_PB_T	0.24142583	1.76098843	0.46865015	0.67457218	3.40126394	1.17872612	2.65568416	3.11723354	0.96570333	1.59767095	0.44734787	6.04984733	2.76929632
ASCVD	54_PB_T	0.26434047	2.85487708	20.6449908	1.123447	3.15886862	1.44065556	2.20724293	5.28680941	0.79302141	2.39228126	0.31720857	7.79804388	5.19429025
ASCVD	59_PB_T	0.28548927	0.7186454	0.44300059	0.69895649	1.5554243	1.25024611	2.55955897	13.2801733	0.47253396	2.71707029	0.66942312	8.06261075	4.19373893
ASCVD	62_PB_T	0.38729055	3.58046159	1.71514385	5.65918432	2.39487828	2.93234271	1.44641164	1.69933607	0.55327221	13.2311097	2.86120771	5.41416377	4.22858046
ASCVD	69_PB_T	0.0865908	3.29663533	9.11677387	0.6741712	1.94210787	2.6843147	4.69445819	6.42008907	1.42874814	4.89856507	8.69000495	5.51707076	3.03067788
ASCVD	6_PB_T	0.04768717	1.64180121	0.59949588	13.0662852	1.97561142	4.05340963	4.70740514	4.29184549	0.55180871	5.50446216	0.72893249	2.54785748	1.60092649
ASCVD	70_PB_T	0.12757427	0.87479497	0.29159832	0.87479497	1.81641456	0.52852196	1.40331693	5.83804143	0.17009902	2.10801288	0.91124476	7.99465403	2.04118826
ASCVD	71_PB_T	0.18142448	2.72721952	2.39948499	0.60864985	1.47480541	0.74325511	2.24732253	2.86182478	0.65546907	6.58395271	3.13103529	5.817288	2.28243694
ASCVD	72_PB_T	0.06191184	4.80435859	1.38682516	1.47350173	1.84497276	2.14214958	6.06736008	7.70183259	0.5324418	3.36800396	5.32441803	8.01139178	3.91282813
ASCVD	81_PB_T	0.07139215	0.12748598	0.03059663	2.99337073	0.93319735	4.00305966	9.53085161	17.1902091	2.13156553	0.52014278	1.67261601	14.4110148	2.47832738
ASCVD	84_PB_T	0.22610793	3.24088031	0.73861923	10.1447091	1.29635213	5.11003919	4.79348809	6.36116973	2.29122701	4.88393126	0.90443172	4.44678927	2.86403377
ASCVD	88_PB_T	0.09401895	2.09011355	0.44116584	0.52795256	3.54379113	1.83698561	3.83308021	1.53323208	1.43198091	2.84949736	0.701526	5.66283359	4.77326969
ASCVD	91_PB_T	0.06335128	4.35012142	0.23228804	0.29563932	0.86580087	6.15563299	4.98891353	1.56794425	0.58072009	6.08172316	3.08837504	4.16534685	2.20145708
ASCVD	99_PB_T	0.04390297	0.82318077	6.47568873	5.0598178	3.06223247	4.19273406	4.61530019	7.00801229	1.91526726	3.18845352	6.9092306	4.28602788	3.00186588

Table S8. Frequencies of peripheral B cell clusters identified by T cell panel.

Group	Sample	B01	B02
NC	107_PB_T	0.609245095	0.390754905
NC	117_PB_T	0.687343358	0.312656642
NC	119_PB_T	0.513616818	0.486383182
NC	130_PB_T	0.607577808	0.392422192
NC	131_PB_T	0.591034212	0.408965788
NC	132_PB_T	0.517456817	0.482543183
NC	24_PB_T	0.689900111	0.310099889
NC	56_PB_T	0.575381141	0.424618859
NC	65_PB_T	0.904391583	0.095608417
NC	73_PB_T	0.776583035	0.223416965
NC	74_PB_T	0.687537994	0.312462006
NC	75_PB_T	0.777869529	0.222130471
NC	86_PB_T	0.717912049	0.282087951
CAS	102_PB_T	0.436559679	0.563440321
CAS	103_PB_T	0.747906198	0.252093802
CAS	104_PB_T	0.647731605	0.352268395
CAS	10_PB_T	0.876281287	0.123718713
CAS	115_PB_T	0.743887895	0.256112105
CAS	116_PB_T	0.619733054	0.380266946
CAS	120_PB_T	0.605002909	0.394997091
CAS	121_PB_T	0.684629901	0.315370099
CAS	124_PB_T	0.613220816	0.386779184
CAS	126_PB_T	0.682025737	0.317974263
CAS	128_PB_T	0.636363636	0.363636364
CAS	19_PB_T	0.774613753	0.225386247
CAS	1_PB_T	0.75162589	0.24837411
CAS	20_PB_T	0.749613601	0.250386399
CAS	23_PB_T	0.84824781	0.15175219
CAS	28_PB_T	0.776756757	0.223243243
CAS	29_PB_T	0.757216495	0.242783505
CAS	30_PB_T	0.659098727	0.340901273
CAS	31_PB_T	0.545544554	0.454455446
CAS	32_PB_T	0.814903846	0.185096154
CAS	35_PB_T	0.680276021	0.319723979
CAS	37_PB_T	0.795302013	0.204697987
CAS	3_PB_T	0.58565589	0.41434411
CAS	46_PB_T	0.767772512	0.232227488
CAS	47_PB_T	0.716988674	0.283011326
CAS	48_PB_T	0.735901387	0.264098613
CAS	49_PB_T	0.737028042	0.262971958
CAS	52_PB_T	0.69273183	0.30726817
CAS	57_PB_T	0.6225	0.3775
CAS	58_PB_T	0.75466954	0.24533046

CAS	63_PB_T	0.48633678	0.51366322
CAS	77_PB_T	0.824554756	0.175445244
CAS	78_PB_T	0.619095477	0.380904523
CAS	79_PB_T	0.591852227	0.408147773
CAS	83_PB_T	0.508834981	0.491165019
CAS	87_PB_T	0.760343184	0.239656816
CAS	90_PB_T	0.411786509	0.588213491
CAS	9_PB_T	0.801060181	0.198939819
ASCVD	101_PB_T	0.698175788	0.301824212
ASCVD	111_PB_T	0.759610028	0.240389972
ASCVD	112_PB_T	0.473396999	0.526603001
ASCVD	114_PB_T	0.735081967	0.264918033
ASCVD	118_PB_T	0.729500891	0.270499109
ASCVD	11_PB_T	0.595299647	0.404700353
ASCVD	127_PB_T	0.845501121	0.154498879
ASCVD	129_PB_T	0.549327354	0.450672646
ASCVD	12_PB_T	0.47601476	0.52398524
ASCVD	133_PB_T	0.685080797	0.314919203
ASCVD	13_PB_T	0.774208145	0.225791855
ASCVD	14_PB_T	0.749872123	0.250127877
ASCVD	15_PB_T	0.806504961	0.193495039
ASCVD	21_PB_T	0.600141044	0.399858956
ASCVD	22_PB_T	0.759190494	0.240809506
ASCVD	25_PB_T	0.909753363	0.090246637
ASCVD	34_PB_T	0.765499635	0.234500365
ASCVD	36_PB_T	0.687991422	0.312008578
ASCVD	4_PB_T	0.635238442	0.364761558
ASCVD	54_PB_T	0.508064516	0.491935484
ASCVD	59_PB_T	0.676224085	0.323775915
ASCVD	62_PB_T	0.440374278	0.559625722
ASCVD	69_PB_T	0.79525593	0.20474407
ASCVD	6_PB_T	0.795010395	0.204989605
ASCVD	70_PB_T	0.680923601	0.319076399
ASCVD	71_PB_T	0.580283794	0.419716206
ASCVD	72_PB_T	0.823178808	0.176821192
ASCVD	81_PB_T	0.871176471	0.128823529
ASCVD	84_PB_T	0.599589322	0.400410678
ASCVD	88_PB_T	0.74602716	0.25397284
ASCVD	91_PB_T	0.774740484	0.225259516
ASCVD	99_PB_T	0.512195122	0.487804878

Table S9. Frequencies of peripheral NK cell clusters identified by T cell panel.

Group	Sample	NK01	NK02	NK03	NK04
NC	107_PB_T	0.044916091	0.573050346	0.212240869	0.169792695
NC	117_PB_T	0.029873418	0.648607595	0.251983122	0.069535865
NC	119_PB_T	0.040110227	0.565829761	0.317819963	0.076240049
NC	130_PB_T	0.021553766	0.766935102	0.098768356	0.112742776
NC	131_PB_T	0.017890576	0.718951529	0.124401914	0.138755981
NC	132_PB_T	0.030091121	0.656071202	0.198135198	0.115702479
NC	24_PB_T	0.01152449	0.026932231	0.720531129	0.241012151
NC	56_PB_T	0.019191227	0.363262509	0.529129541	0.088416724
NC	65_PB_T	0.042066028	0.216187433	0.600106496	0.141640043
NC	73_PB_T	0.020815512	0.128885087	0.654405475	0.195893926
NC	74_PB_T	0.021575758	0.230545455	0.572242424	0.175636364
NC	75_PB_T	0.046082949	0.209677419	0.591589862	0.15264977
NC	86_PB_T	0.028800225	0.491430177	0.196684462	0.283085136
CAS	102_PB_T	0.058784676	0.173051519	0.584544254	0.183619551
CAS	103_PB_T	0.036202186	0.281079235	0.448428962	0.234289617
CAS	104_PB_T	0.047244094	0.322834646	0.319685039	0.31023622
CAS	10_PB_T	0.020564916	0.53716551	0.190535183	0.25173439
CAS	115_PB_T	0.014592275	0.560515021	0.191630901	0.233261803
CAS	116_PB_T	0.027439024	0.489692218	0.31213705	0.170731707
CAS	120_PB_T	0.016243232	0.364014994	0.396084965	0.22365681
CAS	121_PB_T	0.025706941	0.296915167	0.577763496	0.099614396
CAS	124_PB_T	0.017559263	0.204565408	0.621890547	0.155984782
CAS	126_PB_T	0.02970147	0.643430823	0.153659645	0.173208062
CAS	128_PB_T	0.038746527	0.776319852	0.121179376	0.063754245
CAS	19_PB_T	0.018921209	0.435611409	0.407794408	0.137672974
CAS	1_PB_T	0.05867476	0.266059686	0.419069297	0.256196257
CAS	20_PB_T	0.019362898	0.173641474	0.599000625	0.207995003
CAS	23_PB_T	0.028515763	0.201735742	0.631243358	0.138505136
CAS	28_PB_T	0.023760331	0.142355372	0.697520661	0.136363636
CAS	29_PB_T	0.011306533	0.038107203	0.423366834	0.52721943
CAS	30_PB_T	0.027853881	0.316894977	0.337671233	0.317579909
CAS	31_PB_T	0.019436103	0.258675342	0.57007007	0.151818485
CAS	32_PB_T	0.044354839	0.149193548	0.674899194	0.131552419
CAS	35_PB_T	0.030446349	0.126810523	0.631392255	0.211350872
CAS	37_PB_T	0.044850498	0.427325581	0.289451827	0.238372093
CAS	3_PB_T	0.026242236	0.62189441	0.228726708	0.123136646
CAS	46_PB_T	0.025369178	0.077243468	0.390382431	0.507004922
CAS	47_PB_T	0.048625793	0.102536998	0.626849894	0.221987315
CAS	48_PB_T	0.070888469	0.130434783	0.719281664	0.079395085
CAS	49_PB_T	0.019601875	0.219273148	0.616972058	0.144152919
CAS	52_PB_T	0.011640498	0.528965891	0.390092041	0.06930157
CAS	57_PB_T	0.028113763	0.342595619	0.541789256	0.087501362
CAS	58_PB_T	0.008439836	0.152881601	0.469978298	0.368700265

CAS	63_PB_T	0.029866859	0.408420295	0.360921195	0.200791652
CAS	77_PB_T	0.069151911	0.216029823	0.51612302	0.198695247
CAS	78_PB_T	0.027120202	0.233509538	0.610434383	0.128935877
CAS	79_PB_T	0.051480051	0.25971686	0.579150579	0.10965251
CAS	83_PB_T	0.019796041	0.368126375	0.49920016	0.112877425
CAS	87_PB_T	0.032796661	0.573643411	0.328861061	0.064698867
CAS	90_PB_T	0.031809145	0.173459245	0.531063618	0.263667992
CAS	9_PB_T	0.022731044	0.661357226	0.130579061	0.18533267
ASCVD	101_PB_T	0.02894356	0.481620839	0.3276411	0.161794501
ASCVD	111_PB_T	0.013644565	0.220793866	0.611073523	0.154488047
ASCVD	112_PB_T	0.053227633	0.320498301	0.315402039	0.310872027
ASCVD	114_PB_T	0.018473821	0.57547345	0.254363164	0.151689566
ASCVD	118_PB_T	0.05224525	0.503022453	0.238341969	0.206390328
ASCVD	11_PB_T	0.017751479	0.303994083	0.48816568	0.190088757
ASCVD	127_PB_T	0.040560841	0.334501753	0.544316475	0.080620931
ASCVD	129_PB_T	0.023494464	0.688900891	0.188765866	0.098838779
ASCVD	12_PB_T	0.026980482	0.715269805	0.198622273	0.05912744
ASCVD	133_PB_T	0.022813688	0.240086909	0.543183053	0.19391635
ASCVD	13_PB_T	0.036233951	0.505563481	0.198145506	0.260057061
ASCVD	14_PB_T	0.033238636	0.360511364	0.371022727	0.235227273
ASCVD	15_PB_T	0.011286277	0.21766392	0.631673235	0.139376568
ASCVD	21_PB_T	0.014528594	0.331993818	0.580731582	0.072746007
ASCVD	22_PB_T	0.024174053	0.291431641	0.484824067	0.199570239
ASCVD	25_PB_T	0.038357512	0.146053602	0.451438407	0.364150479
ASCVD	34_PB_T	0.039335664	0.133304196	0.481643357	0.345716783
ASCVD	36_PB_T	0.027857829	0.297310279	0.458693564	0.216138329
ASCVD	4_PB_T	0.023925632	0.43790003	0.336558976	0.201615361
ASCVD	54_PB_T	0.042115573	0.308521058	0.402546523	0.246816846
ASCVD	59_PB_T	0.023532488	0.325532749	0.587789253	0.063145509
ASCVD	62_PB_T	0.02116537	0.389264312	0.431212546	0.158357771
ASCVD	69_PB_T	0.015553869	0.391881639	0.431335357	0.161229135
ASCVD	6_PB_T	0.026045777	0.129176532	0.767561168	0.077216522
ASCVD	70_PB_T	0.03369885	0.253008826	0.627707943	0.085584381
ASCVD	71_PB_T	0.01714135	0.17035865	0.654008439	0.158491561
ASCVD	72_PB_T	0.018171045	0.433932451	0.485482915	0.062413589
ASCVD	81_PB_T	0.088761175	0.206683695	0.483184334	0.221370796
ASCVD	84_PB_T	0.034482759	0.405736384	0.449242668	0.110538189
ASCVD	88_PB_T	0.024314336	0.724178175	0.165726512	0.085780976
ASCVD	91_PB_T	0.025061125	0.396852078	0.370262836	0.207823961
ASCVD	99_PB_T	0.040024014	0.507904743	0.314788873	0.137282369

Table S10. Selected features for constructions of disease prediction (DP) model and disease progression prediction (DPP) model.

Specific features in the DP model					
**Clinical features**	**Feature importance**	**Immune features**	**Feature importance**	**Combined features**	**Feature importance**
**Age**	0.10438158	**M02**	0.14750854	**M02**	0.04205231
**TC**	0.19606877	**M03**	0.05274926	**M03**	0.13063799
**TG**	0.2697553	**M06**	0.23596988	**M06**	0.16349131
**HDL**	0.2600464	**M11**	0.10425144	**M11**	0.05681998
**LDL**	0.13314054	**M13**	0.08060714	**M13**	0.0285501
**BMI**	0.03660741	**T05**	0.19965774	**T05**	0.06100333
NA	NA	**T20**	0.1006592	**T20**	0.04678929
NA	NA	**T23**	0.07859678	**T23**	0.03884118
NA	NA	NA	NA	**Age**	0.03527252
NA	NA	NA	NA	**TC**	0.047382
NA	NA	NA	NA	**TG**	0.16919867
NA	NA	NA	NA	**HDL**	0.0816434
NA	NA	NA	NA	**LDL**	0.07310407
NA	NA	NA	NA	**BMI**	0.02521384
**Specific features in the DPP model**					
**Clinical features**	**Feature importance**	**Immune features**	**Feature importance**	**Combined features**	**Feature importance**
**Age**	0.14851783	**M02**	0.14432303	**M02**	0.06041345
**TC**	0.06229127	**M06**	0.12341175	**M06**	0.07411284
**TG**	0.13874855	**M11**	0.06193178	**M11**	0.07768105
**HDL**	0.36880899	**M15**	0.10325625	**M15**	0.1421956
**LDL**	0.13922902	**NK04**	0.04197233	**NK04**	0.02106988
**BMI**	0.14240433	**T05**	0.06390843	**T05**	0.04152788
NA	NA	**T15**	0.03898947	**T15**	0.01296903
NA	NA	**T17**	0.10064766	**T17**	0.08234732
NA	NA	**T20**	0.1595768	**T20**	0.02938045
NA	NA	**T23**	0.1619825	**T23**	0.02599567
NA	NA	NA	NA	**Age**	0.06667321
NA	NA	NA	NA	**TC**	0.04813859
NA	NA	NA	NA	**TG**	0.02928921
NA	NA	NA	NA	**HDL**	0.1463645
NA	NA	NA	NA	**LDL**	0.05032895
NA	NA	NA	NA	**BMI**	0.09151238

Table S11. Sample usages in construction of the DP models and evaluation of model net benefit.

DP model built with clinical features				
**Sample ID**	**Health probability**	**Disease Risk**	**Sample group**	**Dataset**
74_PB	0.90355942	0.09644058	NC	Training
130_PB	0.879092959	0.120907041	NC	Training
86_PB	0.487053639	0.512946361	NC	Training
119_PB	0.870002716	0.129997284	NC	Training
74_PB	0.90355942	0.09644058	NC	Training
119_PB	0.870002716	0.129997284	NC	Training
131_PB	0.839950254	0.160049746	NC	Training
75_PB	0.72633422	0.27366578	NC	Training
74_PB	0.90355942	0.09644058	NC	Training
56_PB	0.670549597	0.329450403	NC	Training
130_PB	0.879092959	0.120907041	NC	Training
73_PB	0.698436919	0.301563081	NC	Training
132_PB	0.606834324	0.393165676	NC	Training
74_PB	0.90355942	0.09644058	NC	Training
56_PB	0.670549597	0.329450403	NC	Training
73_PB	0.698436919	0.301563081	NC	Training
131_PB	0.839950254	0.160049746	NC	Training
74_PB	0.90355942	0.09644058	NC	Training
131_PB	0.839950254	0.160049746	NC	Training
119_PB	0.870002716	0.129997284	NC	Training
107_PB	0.819023399	0.180976601	NC	Training
132_PB	0.606834324	0.393165676	NC	Training
107_PB	0.819023399	0.180976601	NC	Training
130_PB	0.879092959	0.120907041	NC	Training
131_PB	0.839950254	0.160049746	NC	Training
73_PB	0.698436919	0.301563081	NC	Training
107_PB	0.819023399	0.180976601	NC	Training
117_PB	0.522308753	0.477691247	NC	Training
65_PB	0.637655916	0.362344084	NC	Training
73_PB	0.698436919	0.301563081	NC	Training
73_PB	0.698436919	0.301563081	NC	Training

86_PB	0.487053639	0.512946361	NC	Training
131_PB	0.839950254	0.160049746	NC	Training
24_PB	0.78440841	0.21559159	NC	Training
107_PB	0.819023399	0.180976601	NC	Training
75_PB	0.72633422	0.27366578	NC	Training
73_PB	0.698436919	0.301563081	NC	Training
119_PB	0.870002716	0.129997284	NC	Training
119_PB	0.870002716	0.129997284	NC	Training
74_PB	0.90355942	0.09644058	NC	Training
24_PB	0.78440841	0.21559159	NC	Training
74_PB	0.90355942	0.09644058	NC	Training
56_PB	0.670549597	0.329450403	NC	Training
132_PB	0.606834324	0.393165676	NC	Training
75_PB	0.72633422	0.27366578	NC	Training
107_PB	0.819023399	0.180976601	NC	Training
65_PB	0.637655916	0.362344084	NC	Training
56_PB	0.670549597	0.329450403	NC	Training
56_PB	0.670549597	0.329450403	NC	Training
75_PB	0.72633422	0.27366578	NC	Training
116_PB	0.417937079	0.582062921	CAS	Training
102_PB	0.122249288	0.877750712	CAS	Training
126_PB	0.108751526	0.891248474	CAS	Training
79_PB	0.20348196	0.79651804	CAS	Training
30_PB	0.465908186	0.534091814	CAS	Training
124_PB	0.187380166	0.812619834	CAS	Training
3_PB	0.453811863	0.546188137	CAS	Training
120_PB	0.696742593	0.303257407	CAS	Training
23_PB	0.101468864	0.898531136	CAS	Training
28_PB	0.436545818	0.563454182	CAS	Training
83_PB	0.219049019	0.780950981	CAS	Training
115_PB	0.096610731	0.903389269	CAS	Training
77_PB	0.179553467	0.820446533	CAS	Training
10_PB	0.456402906	0.543597094	CAS	Training
29_PB	0.254600684	0.745399316	CAS	Training
32_PB	0.038401913	0.961598087	CAS	Training

87_PB	0.411311335	0.588688665	CAS	Training
52_PB	0.247917727	0.752082273	CAS	Training
121_PB	0.488515063	0.511484937	CAS	Training
48_PB	0.178660867	0.821339133	CAS	Training
46_PB	0.091073371	0.908926629	CAS	Training
104_PB	0.169077277	0.830922723	CAS	Training
19_PB	0.261086242	0.738913758	CAS	Training
90_PB	0.118517705	0.881482295	CAS	Training
78_PB	0.027423077	0.972576923	CAS	Training
112_PB	0.427602532	0.572397468	ASCVD	Training
111_PB	0.082781366	0.917218634	ASCVD	Training
4_PB	0.344833119	0.655166881	ASCVD	Training
88_PB	0.024761905	0.975238095	ASCVD	Training
54_PB	0.34134222	0.65865778	ASCVD	Training
84_PB	0.186580251	0.813419749	ASCVD	Training
129_PB	0.06867982	0.93132018	ASCVD	Training
99_PB	0.074138528	0.925861472	ASCVD	Training
13_PB	0.138429182	0.861570818	ASCVD	Training
71_PB	0.196696324	0.803303676	ASCVD	Training
81_PB	0.024761905	0.975238095	ASCVD	Training
72_PB	0.05714369	0.94285631	ASCVD	Training
6_PB	0.299105214	0.700894786	ASCVD	Training
69_PB	0.336778477	0.663221523	ASCVD	Training
11_PB	0.129545103	0.870454897	ASCVD	Training
133_PB	0.483036162	0.516963838	ASCVD	Training
14_PB	0.019047619	0.980952381	ASCVD	Training
22_PB	0.352417734	0.647582266	ASCVD	Training
91_PB	0.108796703	0.891203297	ASCVD	Training
118_PB	0.206845849	0.793154151	ASCVD	Training
36_PB	0.020659341	0.979340659	ASCVD	Training
114_PB	0.110863601	0.889136399	ASCVD	Training
59_PB	0.364728678	0.635271322	ASCVD	Training
62_PB	0.152112332	0.847887668	ASCVD	Training
127_PB	0.120322677	0.879677323	ASCVD	Training
73_PB	0.698436919	0.301563081	NC	Testing

131_PB	0.839950254	0.160049746	NC	Testing
107_PB	0.819023399	0.180976601	NC	Testing
75_PB	0.72633422	0.27366578	NC	Testing
73_PB	0.698436919	0.301563081	NC	Testing
119_PB	0.870002716	0.129997284	NC	Testing
132_PB	0.606834324	0.393165676	NC	Testing
24_PB	0.78440841	0.21559159	NC	Testing
119_PB	0.870002716	0.129997284	NC	Testing
73_PB	0.698436919	0.301563081	NC	Testing
117_PB	0.522308753	0.477691247	NC	Testing
119_PB	0.870002716	0.129997284	NC	Testing
73_PB	0.698436919	0.301563081	NC	Testing
119_PB	0.870002716	0.129997284	NC	Testing
65_PB	0.637655916	0.362344084	NC	Testing
132_PB	0.606834324	0.393165676	NC	Testing
73_PB	0.698436919	0.301563081	NC	Testing
86_PB	0.487053639	0.512946361	NC	Testing
65_PB	0.637655916	0.362344084	NC	Testing
65_PB	0.637655916	0.362344084	NC	Testing
103_PB	0.485156618	0.514843382	CAS	Testing
128_PB	0.267663135	0.732336865	CAS	Testing
1_PB	0.430215641	0.569784359	CAS	Testing
20_PB	0.152775689	0.847224311	CAS	Testing
31_PB	0.21122947	0.78877053	CAS	Testing
35_PB	0.187066878	0.812933122	CAS	Testing
37_PB	0.204265429	0.795734571	CAS	Testing
47_PB	0.478736198	0.521263802	CAS	Testing
49_PB	0.661593523	0.338406477	CAS	Testing
57_PB	0.19615211	0.80384789	CAS	Testing
58_PB	0.272917277	0.727082723	CAS	Testing
63_PB	0.428539209	0.571460791	CAS	Testing
9_PB	0.529371611	0.470628389	CAS	Testing
101_PB	0.370727526	0.629272474	ASCVD	Testing
12_PB	0.028058608	0.971941392	ASCVD	Testing
15_PB	0.594495047	0.405504953	ASCVD	Testing

21_PB	0.131478148	0.868521852	ASCVD	Testing
25_PB	0.212839792	0.787160208	ASCVD	Testing
34_PB	0.188095849	0.811904151	ASCVD	Testing
70_PB	0.024502165	0.975497835	ASCVD	Testing

**DP model built with immune features**				
**Sample ID**	**Health probability**	**Disease Risk**	**Sample group**	**Dataset**
74_PB	0.726369873	0.273630127	NC	Training
130_PB	0.681238743	0.318761257	NC	Training
86_PB	0.835487283	0.164512717	NC	Training
119_PB	0.66405256	0.33594744	NC	Training
77_PB	0.220972252	0.779027748	CAS	Training
74_PB	0.726369873	0.273630127	NC	Training
119_PB	0.66405256	0.33594744	NC	Training
131_PB	0.529321623	0.470678377	NC	Training
75_PB	0.80796039	0.19203961	NC	Training
74_PB	0.726369873	0.273630127	NC	Training
56_PB	0.882723964	0.117276036	NC	Training
130_PB	0.681238743	0.318761257	NC	Training
73_PB	0.934057793	0.065942207	NC	Training
132_PB	0.936863453	0.063136547	NC	Training
74_PB	0.726369873	0.273630127	NC	Training
56_PB	0.882723964	0.117276036	NC	Training
73_PB	0.934057793	0.065942207	NC	Training
131_PB	0.529321623	0.470678377	NC	Training
74_PB	0.726369873	0.273630127	NC	Training
74_PB	0.726369873	0.273630127	NC	Training
87_PB	0.267319347	0.732680653	CAS	Training
131_PB	0.529321623	0.470678377	NC	Training
119_PB	0.66405256	0.33594744	NC	Training
69_PB	0.214336262	0.785663738	ASCVD	Training
107_PB	0.863559287	0.136440713	NC	Training
132_PB	0.936863453	0.063136547	NC	Training
107_PB	0.863559287	0.136440713	NC	Training
130_PB	0.681238743	0.318761257	NC	Training

131_PB	0.529321623	0.470678377	NC	Training
73_PB	0.934057793	0.065942207	NC	Training
107_PB	0.863559287	0.136440713	NC	Training
117_PB	0.337219227	0.662780773	NC	Training
65_PB	0.895432432	0.104567568	NC	Training
73_PB	0.934057793	0.065942207	NC	Training
73_PB	0.934057793	0.065942207	NC	Training
131_PB	0.529321623	0.470678377	NC	Training
90_PB	0.087133367	0.912866633	CAS	Training
24_PB	0.804760847	0.195239153	NC	Training
107_PB	0.863559287	0.136440713	NC	Training
75_PB	0.80796039	0.19203961	NC	Training
119_PB	0.66405256	0.33594744	NC	Training
119_PB	0.66405256	0.33594744	NC	Training
71_PB	0.124173243	0.875826757	ASCVD	Training
74_PB	0.726369873	0.273630127	NC	Training
24_PB	0.804760847	0.195239153	NC	Training
75_PB	0.80796039	0.19203961	NC	Training
56_PB	0.882723964	0.117276036	NC	Training
132_PB	0.936863453	0.063136547	NC	Training
75_PB	0.80796039	0.19203961	NC	Training
107_PB	0.863559287	0.136440713	NC	Training
65_PB	0.895432432	0.104567568	NC	Training
56_PB	0.882723964	0.117276036	NC	Training
56_PB	0.882723964	0.117276036	NC	Training
116_PB	0.16042094	0.83957906	CAS	Training
102_PB	0.267517076	0.732482924	CAS	Training
126_PB	0.199715978	0.800284022	CAS	Training
81_PB	0.429661847	0.570338153	ASCVD	Training
30_PB	0.304706491	0.695293509	CAS	Training
124_PB	0.034982656	0.965017344	CAS	Training
3_PB	0.106807443	0.893192557	CAS	Training
120_PB	0.015726496	0.984273504	CAS	Training
23_PB	0.213051227	0.786948773	CAS	Training
28_PB	0.241588467	0.758411533	CAS	Training

84_PB	0.080255413	0.919744587	ASCVD	Training
115_PB	0.177110877	0.822889123	CAS	Training
78_PB	0.165416449	0.834583551	CAS	Training
10_PB	0.178185897	0.821814103	CAS	Training
29_PB	0.121279893	0.878720107	CAS	Training
32_PB	0.093737013	0.906262987	CAS	Training
88_PB	0.056026973	0.943973027	ASCVD	Training
52_PB	0.082432207	0.917567793	CAS	Training
121_PB	0.285067009	0.714932991	CAS	Training
48_PB	0.489569711	0.510430289	CAS	Training
46_PB	0.189628954	0.810371046	CAS	Training
104_PB	0.209055072	0.790944928	CAS	Training
19_PB	0.289320332	0.710679668	CAS	Training
91_PB	0.448988789	0.551011211	ASCVD	Training
79_PB	0.370102624	0.629897376	CAS	Training
112_PB	0.168176407	0.831823593	ASCVD	Training
111_PB	0.369691531	0.630308469	ASCVD	Training
4_PB	0.20999602	0.79000398	ASCVD	Training
54_PB	0.135684371	0.864315629	ASCVD	Training
86_PB	0.835487283	0.164512717	NC	Training
129_PB	0.206587829	0.793412171	ASCVD	Training
13_PB	0.125678571	0.874321429	ASCVD	Training
72_PB	0.031758242	0.968241758	ASCVD	Training
83_PB	0.179444805	0.820555195	CAS	Training
73_PB	0.934057793	0.065942207	NC	Training
6_PB	0.098895521	0.901104479	ASCVD	Training
11_PB	0.282540003	0.717459997	ASCVD	Training
133_PB	0.205978577	0.794021423	ASCVD	Training
14_PB	0.232907093	0.767092907	ASCVD	Training
22_PB	0.160247419	0.839752581	ASCVD	Training
99_PB	0.367976542	0.632023458	ASCVD	Training
118_PB	0.185209287	0.814790713	ASCVD	Training
36_PB	0.20910298	0.79089702	ASCVD	Training
114_PB	0.184859946	0.815140054	ASCVD	Training
59_PB	0.237837919	0.762162081	ASCVD	Training

62_PB	0.163293928	0.836706072	ASCVD	Training
127_PB	0.187765873	0.812234127	ASCVD	Training
73_PB	0.934057793	0.065942207	NC	Testing
131_PB	0.529321623	0.470678377	NC	Testing
107_PB	0.863559287	0.136440713	NC	Testing
73_PB	0.934057793	0.065942207	NC	Testing
119_PB	0.66405256	0.33594744	NC	Testing
132_PB	0.936863453	0.063136547	NC	Testing
24_PB	0.804760847	0.195239153	NC	Testing
119_PB	0.66405256	0.33594744	NC	Testing
73_PB	0.934057793	0.065942207	NC	Testing
117_PB	0.337219227	0.662780773	NC	Testing
119_PB	0.66405256	0.33594744	NC	Testing
73_PB	0.934057793	0.065942207	NC	Testing
119_PB	0.66405256	0.33594744	NC	Testing
65_PB	0.895432432	0.104567568	NC	Testing
132_PB	0.936863453	0.063136547	NC	Testing
65_PB	0.895432432	0.104567568	NC	Testing
103_PB	0.266711797	0.733288203	CAS	Testing
128_PB	0.22715036	0.77284964	CAS	Testing
1_PB	0.128087329	0.871912671	CAS	Testing
20_PB	0.128909452	0.871090548	CAS	Testing
31_PB	0.368972898	0.631027102	CAS	Testing
35_PB	0.289944205	0.710055795	CAS	Testing
37_PB	0.211048063	0.788951937	CAS	Testing
47_PB	0.247409993	0.752590007	CAS	Testing
49_PB	0.575458708	0.424541292	CAS	Testing
57_PB	0.210385392	0.789614608	CAS	Testing
58_PB	0.133979354	0.866020646	CAS	Testing
63_PB	0.44427279	0.55572721	CAS	Testing
86_PB	0.835487283	0.164512717	NC	Testing
101_PB	0.305273193	0.694726807	ASCVD	Testing
12_PB	0.292175169	0.707824831	ASCVD	Testing
15_PB	0.127272755	0.872727245	ASCVD	Testing
21_PB	0.1270058	0.8729942	ASCVD	Testing

73_PB	0.934057793	0.065942207	NC	Testing
25_PB	0.20160402	0.79839598	ASCVD	Testing
34_PB	0.130916833	0.869083167	ASCVD	Testing
75_PB	0.80796039	0.19203961	NC	Testing
9_PB	0.249088911	0.750911089	CAS	Testing
70_PB	0.21780489	0.78219511	ASCVD	Testing
65_PB	0.895432432	0.104567568	NC	Testing
**DP model built with combined features**				
**Sample ID**	**Health probability**	**Disease Risk**	**Sample group**	**Dataset**
74_PB	0.730459207	0.269540793	NC	Training
13_PB	0.052661838	0.947338162	ASCVD	Training
86_PB	0.714712436	0.285287564	NC	Training
118_PB	0.07388406	0.92611594	ASCVD	Training
74_PB	0.730459207	0.269540793	NC	Training
119_PB	0.575077645	0.424922355	NC	Training
119_PB	0.575077645	0.424922355	NC	Training
130_PB	0.846659452	0.153340548	NC	Training
75_PB	0.946761998	0.053238002	NC	Training
74_PB	0.730459207	0.269540793	NC	Training
56_PB	0.524004135	0.475995865	NC	Training
117_PB	0.514416916	0.485583084	NC	Training
130_PB	0.846659452	0.153340548	NC	Training
73_PB	0.88455354	0.11544646	NC	Training
132_PB	0.928223443	0.071776557	NC	Training
74_PB	0.730459207	0.269540793	NC	Training
56_PB	0.524004135	0.475995865	NC	Training
73_PB	0.88455354	0.11544646	NC	Training
131_PB	0.65290257	0.34709743	NC	Training
74_PB	0.730459207	0.269540793	NC	Training
131_PB	0.65290257	0.34709743	NC	Training
119_PB	0.575077645	0.424922355	NC	Training
107_PB	0.930956932	0.069043068	NC	Training
127_PB	0.087026696	0.912973304	ASCVD	Training
132_PB	0.928223443	0.071776557	NC	Training

107_PB	0.930956932	0.069043068	NC	Training
130_PB	0.846659452	0.153340548	NC	Training
131_PB	0.65290257	0.34709743	NC	Training
73_PB	0.88455354	0.11544646	NC	Training
107_PB	0.930956932	0.069043068	NC	Training
117_PB	0.514416916	0.485583084	NC	Training
65_PB	0.883409961	0.116590039	NC	Training
73_PB	0.88455354	0.11544646	NC	Training
73_PB	0.88455354	0.11544646	NC	Training
86_PB	0.714712436	0.285287564	NC	Training
131_PB	0.65290257	0.34709743	NC	Training
24_PB	0.92387987	0.07612013	NC	Training
107_PB	0.930956932	0.069043068	NC	Training
75_PB	0.946761998	0.053238002	NC	Training
73_PB	0.88455354	0.11544646	NC	Training
119_PB	0.575077645	0.424922355	NC	Training
119_PB	0.575077645	0.424922355	NC	Training
74_PB	0.730459207	0.269540793	NC	Training
24_PB	0.92387987	0.07612013	NC	Training
74_PB	0.730459207	0.269540793	NC	Training
56_PB	0.524004135	0.475995865	NC	Training
132_PB	0.928223443	0.071776557	NC	Training
75_PB	0.946761998	0.053238002	NC	Training
107_PB	0.930956932	0.069043068	NC	Training
65_PB	0.883409961	0.116590039	NC	Training
56_PB	0.524004135	0.475995865	NC	Training
56_PB	0.524004135	0.475995865	NC	Training
75_PB	0.946761998	0.053238002	NC	Training
116_PB	0.199495393	0.800504607	CAS	Training
102_PB	0.105754607	0.894245393	CAS	Training
124_PB	0.135899295	0.864100705	CAS	Training
79_PB	0.273866966	0.726133034	CAS	Training
30_PB	0.334849428	0.665150572	CAS	Training
121_PB	0.481974831	0.518025169	CAS	Training
3_PB	0.237571151	0.762428849	CAS	Training

23_PB	0.176698884	0.823301116	CAS	Training
28_PB	0.454748057	0.545251943	CAS	Training
83_PB	0.250851732	0.749148268	CAS	Training
115_PB	0.142779748	0.857220252	CAS	Training
77_PB	0.315449911	0.684550089	CAS	Training
10_PB	0.209083694	0.790916306	CAS	Training
29_PB	0.085889527	0.914110473	CAS	Training
32_PB	0.073034854	0.926965146	CAS	Training
87_PB	0.297425352	0.702574648	CAS	Training
52_PB	0.110043457	0.889956543	CAS	Training
120_PB	0.188448884	0.811551116	CAS	Training
48_PB	0.126610445	0.873389555	CAS	Training
46_PB	0.141342741	0.858657259	CAS	Training
104_PB	0.126895826	0.873104174	CAS	Training
19_PB	0.280715312	0.719284688	CAS	Training
90_PB	0.205700882	0.794299118	CAS	Training
78_PB	0.082916167	0.917083833	CAS	Training
112_PB	0.341564519	0.658435481	ASCVD	Training
111_PB	0.125071151	0.874928849	ASCVD	Training
4_PB	0.274961622	0.725038378	ASCVD	Training
88_PB	0.046697192	0.953302808	ASCVD	Training
54_PB	0.177507243	0.822492757	ASCVD	Training
84_PB	0.230772709	0.769227291	ASCVD	Training
99_PB	0.101436508	0.898563492	ASCVD	Training
129_PB	0.038017982	0.961982018	ASCVD	Training
71_PB	0.157159812	0.842840188	ASCVD	Training
81_PB	0.172099512	0.827900488	ASCVD	Training
72_PB	0.139066545	0.860933455	ASCVD	Training
6_PB	0.305583676	0.694416324	ASCVD	Training
69_PB	0.174700688	0.825299312	ASCVD	Training
11_PB	0.149684871	0.850315129	ASCVD	Training
133_PB	0.301885947	0.698114053	ASCVD	Training
14_PB	0.154956793	0.845043207	ASCVD	Training
22_PB	0.229760018	0.770239982	ASCVD	Training
91_PB	0.141788545	0.858211455	ASCVD	Training

36_PB	0.046439449	0.953560551	ASCVD	Training
114_PB	0.148014652	0.851985348	ASCVD	Training
59_PB	0.2533996	0.7466004	ASCVD	Training
62_PB	0.034922022	0.965077978	ASCVD	Training
126_PB	0.147390942	0.852609058	CAS	Training
73_PB	0.88455354	0.11544646	NC	Testing
131_PB	0.65290257	0.34709743	NC	Testing
107_PB	0.930956932	0.069043068	NC	Testing
75_PB	0.946761998	0.053238002	NC	Testing
73_PB	0.88455354	0.11544646	NC	Testing
132_PB	0.928223443	0.071776557	NC	Testing
24_PB	0.92387987	0.07612013	NC	Testing
119_PB	0.575077645	0.424922355	NC	Testing
73_PB	0.88455354	0.11544646	NC	Testing
119_PB	0.575077645	0.424922355	NC	Testing
73_PB	0.88455354	0.11544646	NC	Testing
119_PB	0.575077645	0.424922355	NC	Testing
65_PB	0.883409961	0.116590039	NC	Testing
132_PB	0.928223443	0.071776557	NC	Testing
73_PB	0.88455354	0.11544646	NC	Testing
86_PB	0.714712436	0.285287564	NC	Testing
65_PB	0.883409961	0.116590039	NC	Testing
65_PB	0.883409961	0.116590039	NC	Testing
103_PB	0.256604729	0.743395271	CAS	Testing
1_PB	0.17733486	0.82266514	CAS	Testing
20_PB	0.172158286	0.827841714	CAS	Testing
31_PB	0.35729304	0.64270696	CAS	Testing
35_PB	0.108324509	0.891675491	CAS	Testing
37_PB	0.05291564	0.94708436	CAS	Testing
47_PB	0.304777112	0.695222888	CAS	Testing
49_PB	0.550905567	0.449094433	CAS	Testing
57_PB	0.244062327	0.755937673	CAS	Testing
58_PB	0.185131785	0.814868215	CAS	Testing
63_PB	0.238220502	0.761779498	CAS	Testing
9_PB	0.183001915	0.816998085	CAS	Testing

101_PB	0.240205933	0.759794067	ASCVD	Testing
119_PB	0.575077645	0.424922355	NC	Testing
15_PB	0.325047379	0.674952621	ASCVD	Testing
21_PB	0.039445887	0.960554113	ASCVD	Testing
25_PB	0.271595238	0.728404762	ASCVD	Testing
34_PB	0.103035159	0.896964841	ASCVD	Testing
70_PB	0.051600649	0.948399351	ASCVD	Testing
12_PB	0.132122822	0.867877178	ASCVD	Testing
128_PB	0.238734293	0.761265707	CAS	Testing
117_PB	0.514416916	0.485583084	NC	Testing

Table S12. Sample usages in construction of the DPP models and evaluation of model net benefit.

DPP model built with clinical features				
**Sample ID**	**Health probability**	**Disease Risk**	**Sample group**	**Dataset**
10_PB	0.847492063	0.152507937	CAS	Training
121_PB	0.925672619	0.074327381	CAS	Training
9_PB	0.827660714	0.172339286	CAS	Training
103_PB	0.70025	0.29975	CAS	Training
28_PB	0.670593254	0.329406746	CAS	Training
35_PB	0.398926587	0.601073413	CAS	Training
20_PB	0.618119048	0.381880952	CAS	Training
128_PB	0.691793651	0.308206349	CAS	Training
29_PB	0.848541667	0.151458333	CAS	Training
32_PB	0.577609127	0.422390873	CAS	Training
57_PB	0.75475	0.24525	CAS	Training
3_PB	0.937599206	0.062400794	CAS	Training
46_PB	0.852220238	0.147779762	CAS	Training
63_PB	0.812454365	0.187545635	CAS	Training
116_PB	0.851734127	0.148265873	CAS	Training
126_PB	0.831744048	0.168255952	CAS	Training
104_PB	0.700253968	0.299746032	CAS	Training
58_PB	0.718714286	0.281285714	CAS	Training
30_PB	0.755492063	0.244507937	CAS	Training
52_PB	0.669513889	0.330486111	CAS	Training
83_PB	0.886027778	0.113972222	CAS	Training
124_PB	0.695416667	0.304583333	CAS	Training
47_PB	0.88228373	0.11771627	CAS	Training
87_PB	0.561388889	0.438611111	CAS	Training
49_PB	0.899305556	0.100694444	CAS	Training
118_PB	0.256730159	0.743269841	ASCVD	Training
81_PB	0.068551587	0.931448413	ASCVD	Training
36_PB	0.252281746	0.747718254	ASCVD	Training
22_PB	0.202781746	0.797218254	ASCVD	Training
69_PB	0.499753968	0.500246032	ASCVD	Training
25_PB	0.575214286	0.424785714	ASCVD	Training

62_PB	0.157446429	0.842553571	ASCVD	Training
21_PB	0.294166667	0.705833333	ASCVD	Training
111_PB	0.404	0.596	ASCVD	Training
14_PB	0.030218254	0.969781746	ASCVD	Training
114_PB	0.397496032	0.602503968	ASCVD	Training
11_PB	0.150934524	0.849065476	ASCVD	Training
4_PB	0.38822619	0.61177381	ASCVD	Training
91_PB	0.260263889	0.739736111	ASCVD	Training
84_PB	0.444484127	0.555515873	ASCVD	Training
59_PB	0.43940873	0.56059127	ASCVD	Training
54_PB	0.419130952	0.580869048	ASCVD	Training
101_PB	0.294172619	0.705827381	ASCVD	Training
99_PB	0.324335317	0.675664683	ASCVD	Training
6_PB	0.426170635	0.573829365	ASCVD	Training
15_PB	0.374650794	0.625349206	ASCVD	Training
127_PB	0.324853175	0.675146825	ASCVD	Training
71_PB	0.226142857	0.773857143	ASCVD	Training
72_PB	0.244767857	0.755232143	ASCVD	Training
12_PB	0.125571429	0.874428571	ASCVD	Training
102_PB	0.735047619	0.264952381	CAS	Testing
115_PB	0.561998016	0.438001984	CAS	Testing
120_PB	0.820664683	0.179335317	CAS	Testing
19_PB	0.497728175	0.502271825	CAS	Testing
90_PB	0.612948413	0.387051587	CAS	Testing
23_PB	0.730130952	0.269869048	CAS	Testing
31_PB	0.906553571	0.093446429	CAS	Testing
48_PB	0.835801587	0.164198413	CAS	Testing
37_PB	0.549515873	0.450484127	CAS	Testing
78_PB	0.526501984	0.473498016	CAS	Testing
1_PB	0.571819444	0.428180556	CAS	Testing
77_PB	0.251470238	0.748529762	CAS	Testing
79_PB	0.760444444	0.239555556	CAS	Testing
13_PB	0.502460317	0.497539683	ASCVD	Testing
112_PB	0.473640873	0.526359127	ASCVD	Testing
133_PB	0.637051587	0.362948413	ASCVD	Testing

34_PB	0.188922619	0.811077381	ASCVD	Testing
129_PB	0.120154762	0.879845238	ASCVD	Testing
70_PB	0.149335317	0.850664683	ASCVD	Testing
88_PB	0.079482143	0.920517857	ASCVD	Testing
**DPP model built with immune features**				
**Sample ID**	**Health probability**	**Disease Risk**	**Sample group**	**Dataset**
10_PB	0.738869819	0.261130181	CAS	Training
121_PB	0.601029498	0.398970502	CAS	Training
9_PB	0.708778271	0.291221729	CAS	Training
103_PB	0.537731241	0.462268759	CAS	Training
28_PB	0.721955628	0.278044372	CAS	Training
35_PB	0.691055556	0.308944444	CAS	Training
20_PB	0.745615079	0.254384921	CAS	Training
128_PB	0.421201792	0.578798208	CAS	Training
29_PB	0.480265873	0.519734127	CAS	Training
32_PB	0.718753968	0.281246032	CAS	Training
57_PB	0.498180869	0.501819131	CAS	Training
3_PB	0.739277778	0.260722222	CAS	Training
46_PB	0.589813492	0.410186508	CAS	Training
63_PB	0.631534043	0.368465957	CAS	Training
116_PB	0.711846959	0.288153041	CAS	Training
126_PB	0.739828776	0.260171224	CAS	Training
104_PB	0.649859585	0.350140415	CAS	Training
58_PB	0.763290765	0.236709235	CAS	Training
30_PB	0.60343895	0.39656105	CAS	Training
52_PB	0.616458647	0.383541353	CAS	Training
83_PB	0.604238866	0.395761134	CAS	Training
124_PB	0.505575758	0.494424242	CAS	Training
47_PB	0.755568231	0.244431769	CAS	Training
87_PB	0.817998329	0.182001671	CAS	Training
49_PB	0.528480797	0.471519203	CAS	Training
118_PB	0.230156926	0.769843074	ASCVD	Training
81_PB	0.225646825	0.774353175	ASCVD	Training
36_PB	0.24784632	0.75215368	ASCVD	Training

22_PB	0.169544144	0.830455856	ASCVD	Training
69_PB	0.253384199	0.746615801	ASCVD	Training
25_PB	0.313489177	0.686510823	ASCVD	Training
62_PB	0.430558802	0.569441198	ASCVD	Training
21_PB	0.171270924	0.828729076	ASCVD	Training
111_PB	0.21731615	0.78268385	ASCVD	Training
14_PB	0.510912254	0.489087746	ASCVD	Training
114_PB	0.243454545	0.756545455	ASCVD	Training
11_PB	0.405880508	0.594119492	ASCVD	Training
4_PB	0.435164552	0.564835448	ASCVD	Training
91_PB	0.367506133	0.632493867	ASCVD	Training
84_PB	0.362561377	0.637438623	ASCVD	Training
59_PB	0.279404762	0.720595238	ASCVD	Training
54_PB	0.248352586	0.751647414	ASCVD	Training
101_PB	0.187165945	0.812834055	ASCVD	Training
99_PB	0.295747608	0.704252392	ASCVD	Training
6_PB	0.478587795	0.521412205	ASCVD	Training
15_PB	0.547033467	0.452966533	ASCVD	Training
127_PB	0.245968254	0.754031746	ASCVD	Training
71_PB	0.411187146	0.588812854	ASCVD	Training
72_PB	0.4594614	0.5405386	ASCVD	Training
12_PB	0.293773221	0.706226779	ASCVD	Training
102_PB	0.517534993	0.482465007	CAS	Testing
115_PB	0.775579365	0.224420635	CAS	Testing
120_PB	0.706936508	0.293063492	CAS	Testing
19_PB	0.698310295	0.301689705	CAS	Testing
90_PB	0.647410584	0.352589416	CAS	Testing
23_PB	0.699190526	0.300809474	CAS	Testing
31_PB	0.620325758	0.379674242	CAS	Testing
48_PB	0.754942774	0.245057226	CAS	Testing
37_PB	0.433699495	0.566300505	CAS	Testing
78_PB	0.835440476	0.164559524	CAS	Testing
1_PB	0.732081218	0.267918782	CAS	Testing
77_PB	0.294358225	0.705641775	CAS	Testing
79_PB	0.501972716	0.498027284	CAS	Testing

13_PB	0.769833333	0.230166667	ASCVD	Testing
112_PB	0.256581218	0.743418782	ASCVD	Testing
133_PB	0.15412013	0.84587987	ASCVD	Testing
34_PB	0.667275663	0.332724337	ASCVD	Testing
129_PB	0.099142857	0.900857143	ASCVD	Testing
70_PB	0.259134921	0.740865079	ASCVD	Testing
88_PB	0.333777827	0.666222173	ASCVD	Testing
**DPP model built with combined features**				
**Sample ID**	**Health probability**	**Disease Risk**	**Sample group**	**Dataset**
10_PB	0.891314815	0.108685185	CAS	Training
121_PB	0.651846561	0.348153439	CAS	Training
9_PB	0.713738095	0.286261905	CAS	Training
103_PB	0.841767196	0.158232804	CAS	Training
28_PB	0.946298942	0.053701058	CAS	Training
35_PB	0.320917989	0.679082011	CAS	Training
20_PB	0.57610582	0.42389418	CAS	Training
128_PB	0.562917989	0.437082011	CAS	Training
29_PB	0.857410053	0.142589947	CAS	Training
32_PB	0.789714286	0.210285714	CAS	Training
57_PB	0.538931217	0.461068783	CAS	Training
3_PB	0.893600529	0.106399471	CAS	Training
46_PB	0.622772487	0.377227513	CAS	Training
63_PB	0.551071429	0.448928571	CAS	Training
116_PB	0.547298942	0.452701058	CAS	Training
126_PB	0.508534392	0.491465608	CAS	Training
104_PB	0.790153439	0.209846561	CAS	Training
58_PB	0.78352381	0.21647619	CAS	Training
30_PB	0.670936508	0.329063492	CAS	Training
52_PB	0.838645503	0.161354497	CAS	Training
83_PB	0.471277778	0.528722222	CAS	Training
124_PB	0.617462963	0.382537037	CAS	Training
47_PB	0.504113757	0.495886243	CAS	Training
87_PB	0.746029101	0.253970899	CAS	Training
49_PB	0.875100529	0.124899471	CAS	Training

118_PB	0.314404762	0.685595238	ASCVD	Training
81_PB	0.148248677	0.851751323	ASCVD	Training
36_PB	0.333087302	0.666912698	ASCVD	Training
22_PB	0.17568254	0.82431746	ASCVD	Training
69_PB	0.278304233	0.721695767	ASCVD	Training
25_PB	0.186037037	0.813962963	ASCVD	Training
62_PB	0.248984127	0.751015873	ASCVD	Training
21_PB	0.203743386	0.796256614	ASCVD	Training
111_PB	0.199452381	0.800547619	ASCVD	Training
14_PB	0.382568783	0.617431217	ASCVD	Training
114_PB	0.274097884	0.725902116	ASCVD	Training
11_PB	0.248325397	0.751674603	ASCVD	Training
4_PB	0.385587302	0.614412698	ASCVD	Training
91_PB	0.372835979	0.627164021	ASCVD	Training
84_PB	0.27107672	0.72892328	ASCVD	Training
59_PB	0.455018519	0.544981481	ASCVD	Training
54_PB	0.338269841	0.661730159	ASCVD	Training
101_PB	0.330777778	0.669222222	ASCVD	Training
99_PB	0.28797619	0.71202381	ASCVD	Training
6_PB	0.215126984	0.784873016	ASCVD	Training
15_PB	0.484722222	0.515277778	ASCVD	Training
127_PB	0.445537037	0.554462963	ASCVD	Training
71_PB	0.342074074	0.657925926	ASCVD	Training
72_PB	0.299037037	0.700962963	ASCVD	Training
12_PB	0.17434127	0.82565873	ASCVD	Training
102_PB	0.931838624	0.068161376	CAS	Testing
115_PB	0.498994709	0.501005291	CAS	Testing
120_PB	0.817701058	0.182298942	CAS	Testing
19_PB	0.572952381	0.427047619	CAS	Testing
90_PB	0.862928571	0.137071429	CAS	Testing
23_PB	0.557542328	0.442457672	CAS	Testing
31_PB	0.519309524	0.480690476	CAS	Testing
48_PB	0.629068783	0.370931217	CAS	Testing
37_PB	0.750148148	0.249851852	CAS	Testing
78_PB	0.575333333	0.424666667	CAS	Testing

1_PB	0.74792328	0.25207672	CAS	Testing
77_PB	0.617587302	0.382412698	CAS	Testing
79_PB	0.352015873	0.647984127	CAS	Testing
13_PB	0.22307672	0.77692328	ASCVD	Testing
112_PB	0.424769841	0.575230159	ASCVD	Testing
133_PB	0.531571429	0.468428571	ASCVD	Testing
34_PB	0.304497354	0.695502646	ASCVD	Testing
129_PB	0.420417989	0.579582011	ASCVD	Testing
70_PB	0.634637566	0.365362434	ASCVD	Testing
88_PB	0.143441799	0.856558201	ASCVD	Testing
